# Supplementary material for: Challenging the “old boys club” in academia: Gender and geographic representation in editorial boards of journals publishing in environmental sciences and public health
Source: PLOS Glob Public Health. 2022 Jun 21;2(6):e0000541. doi: 10.1371/journal.pgph.0000541 (PMC10021803; doi:10.1371/journal.pgph.0000541)
Supplement: S1 Fig — (DOCX) [file pgph.0000541.s001.docx]

## S1 Fig. Simple linear regression analyses by journal category.


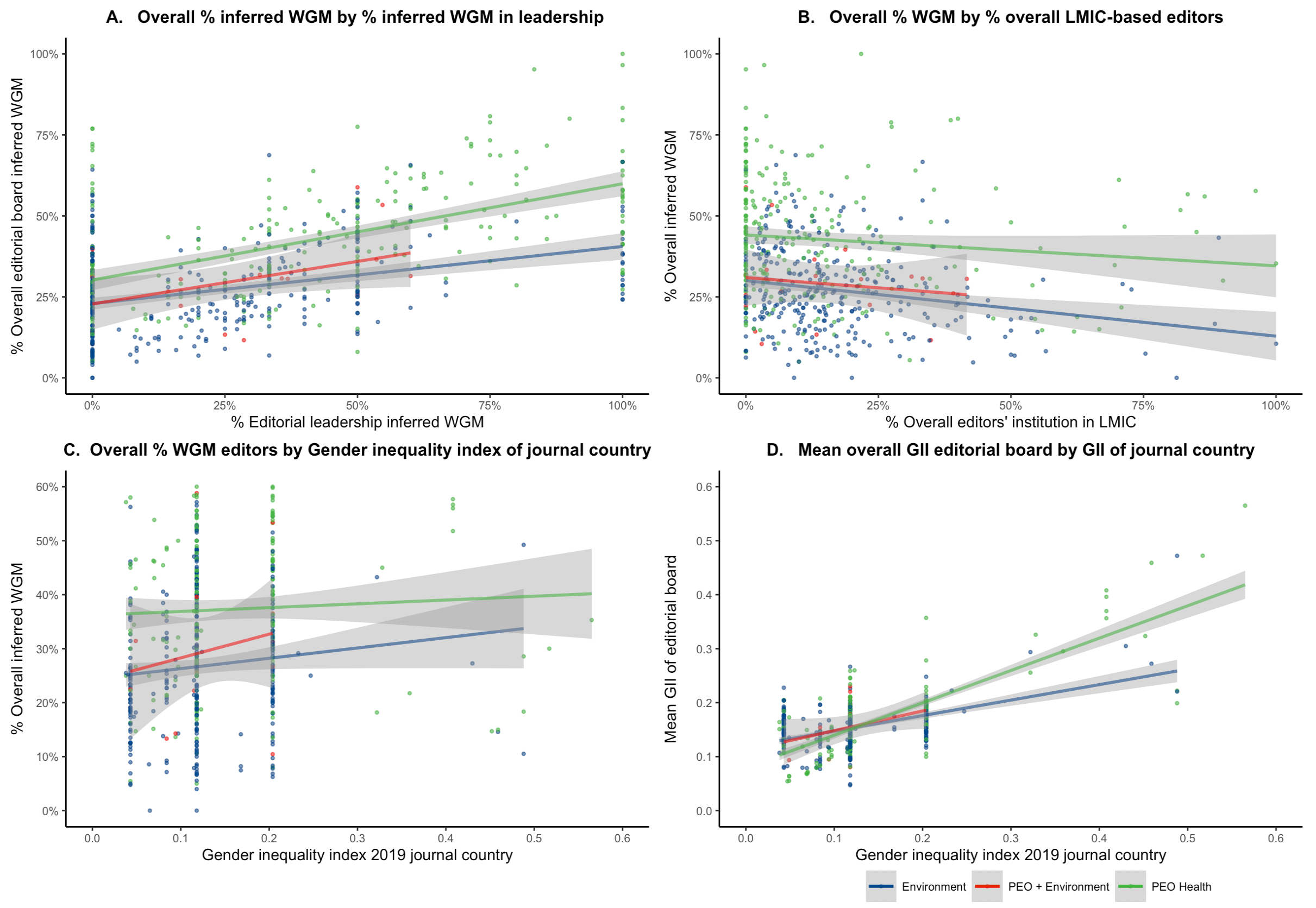


**S1 Fig. Simple linear regression analyses by journal category**. Simple linear regression (unweighted) with 95% confidence intervals; (CI) are depicted in the shaded grey regions.

**A.** Overall % inferred WGM by % inferred WGM in editorial leadership.

**B.** Overall % WGM by % overall LMIC-based editors.

**C.** Overall % inferred WGM by gender inequality index (2019) of the journal country.

**D.** Mean GII of overall editorial board by gender inequality index 2019 of journal country.
