# Supplementary material for: Challenging the “old boys club” in academia: Gender and geographic representation in editorial boards of journals publishing in environmental sciences and public health
Source: PLOS Glob Public Health. 2022 Jun 21;2(6):e0000541. doi: 10.1371/journal.pgph.0000541 (PMC10021803; doi:10.1371/journal.pgph.0000541)
Supplement: S1 Table — (DOCX) [file pgph.0000541.s002.docx]

## Supplement Table 1: Excluded journals with reason for exclusion

| **Journal** | **Category** | **Reason for exclusion** |
| --- | --- | --- |
| *Aerospace Medicine and Human Performance* | PEO Health | No information on full editorial board available |
| *BMC International Health and Human Rights* | PEO Health | Journal merged with *BMC Public Health* |
| *Chemical Speciation and Bioavailability* | Environmental Sciences | Journal renamed to *Environmental Pollutants and Bioavailability* |
| *Ecology Law Quarterly* | Environmental Studies | Student publication |
| *Environment* | Environmental Sciences, Environmental Studies | No information on full editorial board available |
| *Gefahrstoffe Reinhaltung Der Luft* | Environmental Sciences | No information on full editorial board available |
| *Harvard Environmental Law Review* | Environmental Studies | Student publication |
| *International Journal of Occupational and Environmental Health* | PEO Health | Journal discontinued in 2018 |
| *International Perspectives on Sexual and Reproductive Health* | PEO Health | Journal discontinued in 2020 |
| *Journal of Coastal Research* | Environmental Sciences | No information on full editorial board available |
| *Journal of Environmental* | PEO Health, Environmental Sciences | No information on full editorial board available |
| *Natural Resources Journal* | Environmental Studies | Student publication |
| *WHO Technical Report Series* | PEO Health | No information on full editorial board available |

**PEO:** public, environmental and occupational
