# Supplementary material for: Challenging the “old boys club” in academia: Gender and geographic representation in editorial boards of journals publishing in environmental sciences and public health
Source: PLOS Glob Public Health. 2022 Jun 21;2(6):e0000541. doi: 10.1371/journal.pgph.0000541 (PMC10021803; doi:10.1371/journal.pgph.0000541)
Supplement: S2 Table — (DOCX) [file pgph.0000541.s003.docx]

## Supplement Table 2: Journals excluded due to incomplete inferred gender data

| **Journal** | **Category** | **Reason for exclusion** |
| --- | --- | --- |
| *Aquatic Ecosystem Health & Management* | Environmental Sciences | Journals >10% incomplete data on gender |
| *Arid Land Research and Management* | Environmental Sciences | Journals >10% incomplete data on gender |
| *Biomedical and Environmental Sciences* | PEO Health, Environmental Sciences | Journals >10% incomplete data on gender |
| *Carpathian Journal of Earth and Environmental Sciences* | Environmental Sciences | Journals >10% incomplete data on gender |
| *Central European Journal of Public Health* | PEO Health | Journals >10% incomplete data on gender |
| *Chinese Geographical Science* | Environmental Sciences | Journals >10% incomplete data on gender |
| *International Journal of Environmental Health Research* | PEO Health, Environmental Sciences | Journals >10% incomplete data on gender |
| *Iranian Journal of Public Health* | PEO Health | Journals >10% incomplete data on gender |
| *Journal of Environmental Biology* | Environmental Sciences | Journals >10% incomplete data on gender |
| *Journal of Environmental Management* | Environmental Sciences | Journals >10% incomplete data on gender |
| *Journal of Environmental Protection and Ecology* | Environmental Sciences | Journals >10% incomplete data on gender |
| *Journal of Hazardous Materials* | Environmental Sciences | Journals >10% incomplete data on gender |
| *Journal of Hydro-environment Research* | Environmental Sciences | Journals >10% incomplete data on gender |
| *Journal of Material Cycles and Waste Management* | Environmental Sciences | Journals >10% incomplete data on gender |
| *Journal of Mountain Science* | Environmental Sciences | Journals >10% incomplete data on gender |
| *Journal of Radiological Protection* | PEO Health, Environmental Sciences | Journals >10% incomplete data on gender |
| *Journal of Safety Research* | PEO Health | Journals >10% incomplete data on gender |
| *Journal of Tropical Medicine* | PEO Health | Journals >10% incomplete data on gender |
| *Occupational Medicine-Oxford* | PEO Health | Journals >10% incomplete data on gender |
| *Progress in Planning* | Environmental Studies | Journals >10% incomplete data on gender |
| *Radiation Protection Dosimetry* | PEO Health, Environmental Sciences | Journals >10% incomplete data on gender |
| *Tropical Doctor* | PEO Health | Journals >10% incomplete data on gender |
| *Water Air and Soil Pollution* | Environmental Sciences | Journals >10% incomplete data on gender |

**PEO:** public, environmental and occupational
