# Supplementary material for: Challenging the “old boys club” in academia: Gender and geographic representation in editorial boards of journals publishing in environmental sciences and public health
Source: PLOS Glob Public Health. 2022 Jun 21;2(6):e0000541. doi: 10.1371/journal.pgph.0000541 (PMC10021803; doi:10.1371/journal.pgph.0000541)
Supplement: S3 Table — (DOCX) [file pgph.0000541.s004.docx]

## Supplement Table 3: Characteristics of journals categorized as Public, Environmental and Occupational Health Journals following the Journal Citation Reports (JCR)

| **Journal Title** | **IF** | **H** | **Country** | **Publisher** | **Coverage** | **Total** | **%**  **(N) Inferred women and gender minority** | | | | | | **%**  **(N) UN region of editors’ institutions** | | | | | | **%**  **(N) Income group of editors' institution** | | | | | |
| --- | --- | --- | --- | --- | --- | --- | --- | --- | --- | --- | --- | --- | --- | --- | --- | --- | --- | --- | --- | --- | --- | --- | --- | --- |
|  |  |  |  |  |  |  | **EiC** | **EL** | **EB** | **AB** | **EC** | **Tot** | **Unkn** | **AF** | **AP** | **EE** | **LAC** | **WEO** | **Unkn** | **HIC** | **UMIC** | **LMIC** | **LIC** | **Unkn** |
| Accident Analysis & Prevention | 3.655 | 152 | United Kingdom | Elsevier Ltd. | 1969-2020 | 87 | 0%  (0) | 17.6%  (3) | 17.6%  (3) | 15.7%  (11) | NA | 16%  (14) | 0%  (0) | NA | 11.5%  (10) | NA | 2.3%  (2) | 86.2%  (75) | NA | 90.8%  (79) | 9.2%  (8) | NA | NA | NA |
| Administration and Policy in Mental Health and Mental Health Services Research | 2.313 | 68 | United States | Springer New York | 1988-2020 | 43 | 0%  (0) | 50%  (5) | 46.5%  (20) | NA | NA | 46%  (20) | 0%  (0) | NA | 2.3%  (1) | NA | NA | 97.7%  (42) | NA | 100%  (43) | NA | NA | NA | NA |
| African Journal of Reproductive Health | 0.711 | 41 | Nigeria | Women's Health and Action Research Centre | 1997-1998, 2000-2020 | 37 | 0%  (0) | 0%  (0) | 25%  (1) | 29%  (9) | NA | 28%  (10) | 5%  (2) | 54.1%  (20) | NA | NA | NA | 45.9%  (17) | NA | 45.9%  (17) | 16.2%  (6) | 37.8%  (14) | NA | NA |
| AIDS and Behavior | 3.147 | 106 | United States | Springer New York | 1997-2020 | 105 | 0%  (0) | 40%  (4) | 45.2%  (47) | NA | NA | 46%  (47) | 1%  (1) | 11.4%  (12) | 1.9%  (2) | NA | 2.9%  (3) | 83.8%  (88) | NA | 84.8%  (89) | 11.4%  (12) | 1%  (1) | 2.9%  (3) | NA |
| AIDS Care-Psychological and Socio-Medical Aspects of AIDS/HIV | 1.894 | 97 | United Kingdom | Routledge | 1989-2020 | 51 | 100%  (1) | 75%  (3) | 43.1%  (22) | NA | NA | 44%  (22) | 0%  (0) | 5.9%  (3) | 2%  (1) | NA | NA | 92.2%  (47) | NA | 94.1%  (48) | 5.9%  (3) | NA | NA | NA |
| AIDS Education and Prevention | 1.524 | 73 | United States | Guilford Publications | 1989-2020 | 45 | 0%  (0) | 0%  (0) | 26.7%  (12) | NA | NA | 26%  (12) | 0%  (0) | 2.2%  (1) | 4.4%  (2) | NA | NA | 93.3%  (42) | NA | 93.3%  (42) | 4.4%  (2) | 2.2%  (1) | NA | NA |
| AIDS Patient Care and STDS | 3.795 | 85 | United States | Mary Ann Liebert Inc. | 1996-2020 | 33 | 0%  (0) | 20%  (1) | 36.4%  (12) | NA | NA | 36%  (12) | 0%  (0) | 3%  (1) | 18.2%  (6) | NA | 3%  (1) | 75.8%  (25) | NA | 84.8%  (28) | 12.1%  (4) | 3%  (1) | NA | NA |
| AJAR-African Journal of AIDS Research | 0.888 | 28 | United Kingdom | Taylor and Francis Ltd. | 2003-2020 | 26 | 0%  (0) | 50%  (5) | 48%  (12) | NA | NA | 48%  (12) | 4%  (1) | 50%  (13) | NA | NA | 3.8%  (1) | 46.2%  (12) | NA | 50%  (13) | 30.8%  (8) | 15.4%  (4) | 3.8%  (1) | NA |
| American Journal of Community Psychology | 1.509 | 112 | United States | Wiley-Blackwell | 1973-2020 | 87 | 100%  (1) | 57.1%  (4) | 57.5%  (50) | NA | NA | 58%  (50) | 0%  (0) | NA | 1.1%  (1) | NA | 2.3%  (2) | 96.6%  (84) | NA | 98.9%  (86) | 1.1%  (1) | NA | NA | NA |
| American Journal of Epidemiology | 4.526 | 256 | United Kingdom | Oxford University Press | 1921-2020 | 57 | 0%  (0) | 50%  (21) | 52.6%  (30) | NA | NA | 52%  (30) | 0%  (0) | NA | 1.8%  (1) | NA | NA | 98.2%  (56) | NA | 98.2%  (56) | NA | 1.8%  (1) | NA | NA |
| American Journal of Health Behavior | 1.224 | 68 | United States | PNG Publications | 1996-2020 | 67 | 0%  (0) | 20%  (2) | 46.3%  (31) | NA | NA | 46%  (31) | 0%  (0) | 1.5%  (1) | 3%  (2) | NA | 1.5%  (1) | 94%  (63) | NA | 97%  (65) | 3%  (2) | NA | NA | NA |
| American Journal of Health Promotion | 2.232 | 91 | United States | SAGE Publications Inc. | 1986-2020 | 137 | 0%  (0) | 60%  (3) | 53.3%  (73) | NA | NA | 54%  (73) | 0%  (0) | NA | 1.5%  (2) | NA | 0.7%  (1) | 97.8%  (134) | NA | 98.5%  (135) | 0.7%  (1) | 0.7%  (1) | NA | NA |
| American Journal of Industrial Medicine | 1.739 | 104 | United States | Wiley-Liss Inc. | 1980-2020 | 65 | 0%  (0) | 14.3%  (2) | 29%  (18) | 50%  (1) | NA | 30%  (19) | 2%  (1) | 4.6%  (3) | 3.1%  (2) | NA | 1.5%  (1) | 90.8%  (59) | NA | 93.8%  (61) | 6.2%  (4) | NA | NA | NA |
| American Journal of Infection Control | 2.294 | 107 | United States | Mosby Inc. | 1980-2020 | 51 | 100%  (1) | 81.8%  (9) | 64.7%  (33) | NA | NA | 64%  (33) | 0%  (0) | NA | 2%  (1) | NA | 3.9%  (2) | 94.1%  (48) | NA | 96.1%  (49) | 3.9%  (2) | NA | NA | NA |
| American Journal of Mens Health | 1.605 | 34 | United States | SAGE Publications Inc. | 2007-2020 | 26 | 0%  (0) | 50%  (1) | 8.3%  (2) | 0%  (0) | NA | 8%  (2) | 4%  (1) | NA | NA | NA | NA | 100%  (26) | NA | 100%  (26) | NA | NA | NA | NA |
| American Journal of Preventive Medicine | 4.42 | 216 | United States | Elsevier Inc. | 1985-2020 | 26 | 0%  (0) | 20%  (1) | 26.9%  (7) | NA | NA | 26%  (7) | 0%  (0) | NA | 3.8%  (1) | NA | NA | 96.2%  (25) | NA | 96.2%  (25) | NA | 3.8%  (1) | NA | NA |
| American Journal of Public Health | 6.464 | 264 | United States | American Public Health Association Inc. | 1949-1963, 1971-2020 | 40 | 0%  (0) | 34.8%  (8) | 47.5%  (19) | NA | 0%  (0) | 48%  (19) | 0%  (0) | NA | NA | NA | 2.5%  (1) | 97.5%  (39) | NA | 97.5%  (39) | 2.5%  (1) | NA | NA | NA |
| American Journal of Tropical Medicine and Hygiene | 2.126 | 151 | United States | American Society of Tropical Medicine and Hygiene | 1945-2020 | 29 | 0%  (0) | 40%  (6) | 27.6%  (8) | NA | NA | 28%  (8) | 0%  (0) | NA | NA | NA | 6.9%  (2) | 93.1%  (27) | NA | 93.1%  (27) | 6.9%  (2) | NA | NA | NA |
| Anales del Sistema Sanitario De Navarra | 0.829 | 23 | Spain | Gobierno de Navarra | 1999-2020 | 13 | 0%  (0) | 0%  (0) | 53.8%  (7) | NA | NA | 54%  (7) | 0%  (0) | NA | NA | NA | NA | 100%  (13) | NA | 100%  (13) | NA | NA | NA | NA |
| Analytic Methods in Accident Research | 9.179 | 35 | United Kingdom | Elsevier BV | 2014-2020 | 40 | 0%  (0) | 0%  (0) | 0%  (0) | 6.1%  (2) | NA | 4%  (2) | 0%  (0) | NA | 12.5%  (5) | NA | 2.5%  (1) | 85%  (34) | NA | 90%  (36) | 10%  (4) | NA | NA | NA |
| Annali dell'Istituto Superiore di Sanita | 1.37 | 42 | Italy | Istituto Superiore di Sanita | 1965-2020 | 22 | 0%  (0) | 0%  (0) | 22.2%  (2) | 38.5%  (5) | NA | 32%  (7) | 0%  (0) | NA | NA | 4.5%  (1) | NA | 95.5%  (21) | NA | 100%  (22) | NA | NA | NA | NA |
| Annals of Epidemiology | 2.573 | 122 | United States | Elsevier Inc. | 1990-2020 | 30 | 0%  (0) | 33.3%  (5) | 30%  (9) | NA | NA | 30%  (9) | 0%  (0) | NA | 10%  (3) | NA | NA | 90%  (27) | NA | 100%  (30) | NA | NA | NA | NA |
| Annals of Global Health | 1.192 | 66 | United States | Levy Library Press | 2014-2020 | 47 | 0%  (0) | 0%  (0) | 29.8%  (14) | NA | NA | 30%  (14) | 0%  (0) | 6.4%  (3) | 8.5%  (4) | NA | 4.3%  (2) | 80.9%  (38) | NA | 85.1%  (40) | 8.5%  (4) | 4.3%  (2) | 2.1%  (1) | NA |
| Annals of Human Biology | 1.535 | 57 | United Kingdom | Informa Healthcare | 1974-2020 | 37 | 66.7%  (2) | 66.7%  (2) | 48.6%  (18) | NA | NA | 48%  (18) | 0%  (0) | 2.7%  (1) | 8.1%  (3) | 5.4%  (2) | 2.7%  (1) | 81.1%  (30) | NA | 83.8%  (31) | 13.5%  (5) | 2.7%  (1) | NA | NA |
| Annals of Work Exposures and Health | 1.96 | 77 | United Kingdom | Oxford University Press | 2017-2020 | 17 | 100%  (1) | 100%  (1) | 41.2%  (7) | NA | NA | 42%  (7) | 0%  (0) | NA | NA | NA | NA | 94.1%  (16) | 5.9%  (1) | 94.1%  (16) | NA | NA | NA | 5.9%  (1) |
| Annual Review of Public Health | 16.463 | 144 | United States | Annual Reviews Inc. | 1980-2020 | 7 | 0%  (0) | 0%  (0) | 28.6%  (2) | NA | NA | 28%  (2) | 0%  (0) | NA | NA | NA | NA | 100%  (7) | NA | 100%  (7) | NA | NA | NA | NA |
| Anthropology & Medicine | 1.125 | 31 | United Kingdom | Routledge | 1997-2020 | 65 | 0%  (0) | 42.9%  (3) | 41.5%  (27) | NA | NA | 42%  (27) | 0%  (0) | NA | 6.2%  (4) | NA | 1.5%  (1) | 92.3%  (60) | NA | 92.3%  (60) | 3.1%  (2) | 4.6%  (3) | NA | NA |
| Antimicrobial Resistance and Infection Control | 3.594 | 37 | United Kingdom | BioMed Central Ltd. | 2012-2020 | 55 | 100%  (1) | 48%  (12) | 43.6%  (24) | NA | NA | 44%  (24) | 0%  (0) | 12.7%  (7) | 12.7%  (7) | 1.8%  (1) | 3.6%  (2) | 69.1%  (38) | NA | 74.5%  (41) | 16.4%  (9) | 9.1%  (5) | NA | NA |
| Archives des Maladies Professionnelles et de l Environnement | 0.325 | 10 | France | Elsevier Masson | 2004-2020 | 15 | 0%  (0) | 44.4%  (4) | 26.7%  (4) | NA | NA | 26%  (4) | 0%  (0) | 13.3%  (2) | NA | NA | NA | 86.7%  (13) | NA | 86.7%  (13) | NA | 13.3%  (2) | NA | NA |
| Archives of Public Health | 1.774 | 28 | United Kingdom | BioMed Central Ltd. | 1996-2006, 2008-2020 | 35 | 0%  (0) | 54.5%  (6) | 40%  (14) | NA | NA | 40%  (14) | 0%  (0) | 2.9%  (1) | 14.3%  (5) | NA | NA | 82.9%  (29) | NA | 82.9%  (29) | 2.9%  (1) | 11.4%  (4) | 2.9%  (1) | NA |
| Arhiv za Higijenu Rada i Toksikologiju-Archives of Industrial Hygiene and Toxicology | 1.727 | 32 | Croatia | Institute for Medical Research and Occupational Health | 1953-2020 | 20 | 100%  (1) | 100%  (1) | 70%  (14) | NA | NA | 70%  (14) | 0%  (0) | NA | NA | 100%  (20) | NA | NA | NA | 100%  (20) | NA | NA | NA | NA |
| Arts & Health | 1.15 | 15 | United Kingdom | Taylor and Francis Ltd. | 2012-2020 | 30 | 100%  (2) | 100%  (4) | 66.7%  (20) | NA | NA | 66%  (20) | 0%  (0) | NA | 3.3%  (1) | NA | 3.3%  (1) | 93.3%  (28) | NA | 100%  (30) | NA | NA | NA | NA |
| Asia-Pacific Journal of Public Health | 1.255 | 37 | United States | SAGE Publications Inc. | 1987-1992, 1994-1996, 1998-2020 | 23 | 0%  (0) | 0%  (0) | 46.2%  (6) | 20%  (2) | NA | 34%  (8) | 0%  (0) | 4.3%  (1) | 82.6%  (19) | NA | NA | 13%  (3) | NA | 30.4%  (7) | 56.5%  (13) | 13%  (3) | NA | NA |
| Asian Pacific Journal of Tropical Medicine | 1.94 | 52 | India | Wolters Kluwer Medknow Publications | 2010-2020 | 60 | 25%  (1) | 14.3%  (1) | 19%  (11) | 0%  (0) | NA | 18%  (11) | 0%  (0) | 8.3%  (5) | 38.3%  (23) | 3.3%  (2) | 8.3%  (5) | 41.7%  (25) | NA | 53.3%  (32) | 28.3%  (17) | 18.3%  (11) | NA | NA |
| Australian and New Zealand Journal of Public Health | 2.079 | 76 | United States | Wiley-Blackwell | 1996-2020 | 28 | 0%  (0) | 87.5%  (7) | 50%  (14) | NA | NA | 50%  (14) | 0%  (0) | NA | NA | NA | NA | 100%  (28) | NA | 100%  (28) | NA | NA | NA | NA |
| Australian Journal of Primary Health | 0.975 | 30 | Australia | CSIRO | 2001-2020 | 21 | 100%  (1) | 71.4%  (5) | 71.4%  (15) | NA | NA | 72%  (15) | 0%  (0) | NA | 4.8%  (1) | NA | NA | 95.2%  (20) | NA | 95.2%  (20) | 4.8%  (1) | NA | NA | NA |
| Australian Journal of Rural Health | 1.464 | 49 | United Kingdom | Wiley-Blackwell Publishing Ltd | 1992-2020 | 29 | 0%  (0) | 50%  (7) | 33.3%  (9) | 50%  (1) | NA | 34%  (10) | 0%  (0) | NA | NA | NA | NA | 100%  (29) | NA | 100%  (29) | NA | NA | NA | NA |
| BMC Public Health | 2.521 | 143 | United Kingdom | BioMed Central Ltd. | 2001-2020 | 736 | NA | 30.8%  (4) | 39.8%  (292) | 50%  (1) | NA | 40%  (293) | 0%  (0) | 8.8%  (65) | 21.7%  (160) | 2.2%  (16) | 2.4%  (18) | 64.8%  (477) | NA | 72.1%  (531) | 13%  (96) | 12.8%  (94) | 2%  (15) | NA |
| BMC Womens Health | 1.544 | 47 | United Kingdom | BioMed Central Ltd. | 2001-2020 | 106 | 100%  (1) | 57.1%  (4) | 47.6%  (50) | 100%  (1) | NA | 48%  (51) | 0%  (0) | 14.2%  (15) | 18.9%  (20) | 2.8%  (3) | 2.8%  (3) | 61.3%  (65) | NA | 65.1%  (69) | 17.9%  (19) | 16%  (17) | 0.9%  (1) | NA |
| BMJ Global Health | 4.28 | 32 | United Kingdom | BMJ Publishing Group | 2016-2020 | 37 | 0%  (0) | 0%  (0) | 26.7%  (4) | 50%  (11) | NA | 40%  (15) | 0%  (0) | 13.5%  (5) | 18.9%  (7) | NA | 2.7%  (1) | 64.9%  (24) | NA | 64.9%  (24) | 5.4%  (2) | 27%  (10) | 2.7%  (1) | NA |
| Bulletin of the World Health Organization | 6.96 | 168 | Switzerland | World Health Organization | 1945, 1949-2020 | 56 | 100%  (1) | 100%  (1) | 31.2%  (5) | 55%  (22) | NA | 48%  (27) | 0%  (0) | 1.8%  (1) | 14.3%  (8) | NA | NA | 83.9%  (47) | NA | 87.5%  (49) | 3.6%  (2) | 8.9%  (5) | NA | NA |
| Bundesgesundheitsblatt-Gesundheitsforschung-Gesundheitsschutz | 1.059 | 62 | Germany | Springer Verlag | 1999-2020 | 16 | NA | NA | 31.2%  (5) | NA | NA | 32%  (5) | 0%  (0) | NA | NA | NA | NA | 100%  (16) | NA | 100%  (16) | NA | NA | NA | NA |
| Cadernos de Saude Publica | 1.408 | 77 | Brazil | Fundacao Oswaldo Cruz | 1998-2020 | 30 | 100%  (3) | 100%  (3) | 56.7%  (17) | NA | NA | 56%  (17) | 0%  (0) | NA | NA | NA | 83.3%  (25) | 16.7%  (5) | NA | 16.7%  (5) | 83.3%  (25) | NA | NA | NA |
| Canadian Journal of Public Health – Revue Canadienne De Sante Publique | 1.638 | 72 | Canada | Canadian Public Health Association | 1945-2020 | 47 | 100%  (1) | 41.7%  (5) | 63.8%  (30) | NA | NA | 64%  (30) | 0%  (0) | NA | NA | NA | NA | 100%  (47) | NA | 100%  (47) | NA | NA | NA | NA |
| Cancer Causes & Control | 2.375 | 134 | Netherlands | Springer Netherlands | 1990-2020 | 61 | 100%  (1) | 85.7%  (30) | 71.7%  (43) | NA | NA | 72%  (43) | 2%  (1) | NA | 4.9%  (3) | NA | NA | 95.1%  (58) | NA | 100%  (61) | NA | NA | NA | NA |
| Cancer Epidemiology | 2.179 | 75 | Netherlands | Elsevier BV | 2009-2020 | 27 | 100%  (1) | 60%  (3) | 61.5%  (16) | NA | NA | 62%  (16) | 4%  (1) | NA | 11.1%  (3) | NA | 3.7%  (1) | 85.2%  (23) | NA | 92.6%  (25) | 7.4%  (2) | NA | NA | NA |
| Cancer Epidemiology Biomarkers & Prevention | 4.344 | 192 | United States | American Association for Cancer Research Inc. | 1983, 1986, 1991-2020 | 164 | 100%  (1) | 56.7%  (17) | 48.1%  (78) | NA | NA | 48%  (78) | 1%  (2) | 0.6%  (1) | 1.8%  (3) | NA | 0.6%  (1) | 97%  (159) | NA | 98.2%  (161) | 1.2%  (2) | 0.6%  (1) | NA | NA |
| Central European Journal of Public Health | 0.653 | 33 | Czech Republic | Czech National Institute of Public Health | 1993-2020 | 30 | NA | NA | 29.2%  (7) | NA | NA | 30%  (7) | 20%  (6) | NA | NA | 63.3%  (19) | NA | 36.7%  (11) | NA | 96.7%  (29) | 3.3%  (1) | NA | NA | NA |
| Children’s Health Care | 0.574 | 38 | United States | Routledge | 1980-2020 | 62 | 0%  (0) | 33.3%  (1) | 45.2%  (28) | NA | NA | 46%  (28) | 0%  (0) | NA | NA | NA | NA | 100%  (62) | NA | 100%  (62) | NA | NA | NA | NA |
| Ciencia & Saude Coletiva | 1.019 | 46 | Brazil | Associacao Brasileira de Pos - Graduacao em Saude Coletiva | 2006-2020 | 112 | 33.3%  (1) | 73.9%  (17) | 51.8%  (58) | NA | NA | 52%  (58) | 0%  (0) | 0.9%  (1) | NA | NA | 81.2%  (91) | 17.9%  (20) | NA | 17.9%  (20) | 81.2%  (91) | NA | NA | 0.9%  (1) |
| Clinical Epidemiology | 2.942 | 58 | New Zealand | Dove Medical Press Ltd. | 2010-2020 | 21 | 0%  (0) | 40%  (2) | 33.3%  (7) | NA | NA | 34%  (7) | 0%  (0) | NA | 9.5%  (2) | 9.5%  (2) | NA | 81%  (17) | NA | 81%  (17) | 19%  (4) | NA | NA | NA |
| Community Dentistry and Oral Epidemiology | 2.135 | 101 | Denmark | Blackwell Munksgaard | 1973-2020 | 7 | 0%  (0) | 0%  (0) | 57.1%  (4) | NA | NA | 58%  (4) | 0%  (0) | NA | NA | NA | 14.3%  (1) | 85.7%  (6) | NA | 85.7%  (6) | 14.3%  (1) | NA | NA | NA |
| Community Mental Health Journal | 1.292 | 66 | United States | Springer Netherlands | 1965-2020 | 29 | 100%  (1) | 100%  (5) | 100%  (5) | 29.2%  (7) | NA | 42%  (12) | 0%  (0) | NA | NA | NA | NA | 100%  (29) | NA | 100%  (29) | NA | NA | NA | NA |
| Conflict and Health | 2 | 30 | United Kingdom | BioMed Central Ltd. | 2010-2020 | 34 | 0%  (0) | 57.1%  (4) | 38.2%  (13) | NA | NA | 38%  (13) | 0%  (0) | 2.9%  (1) | NA | NA | NA | 97.1%  (33) | NA | 97.1%  (33) | 2.9%  (1) | NA | NA | NA |
| Critical Public Health | 2.525 | 46 | United Kingdom | Routledge | 1990-1995, 1997-2020 | 48 | 100%  (2) | 50%  (3) | 37.5%  (18) | NA | NA | 38%  (18) | 0%  (0) | 6.2%  (3) | 4.2%  (2) | NA | 2.1%  (1) | 87.5%  (42) | NA | 93.8%  (45) | 4.2%  (2) | 2.1%  (1) | NA | NA |
| Disability and Health Journal | 2.056 | 36 | United States | Elsevier Inc. | 2008-2020 | 30 | 100%  (2) | 100%  (2) | 66.7%  (20) | NA | NA | 66%  (20) | 0%  (0) | NA | NA | NA | NA | 100%  (30) | NA | 100%  (30) | NA | NA | NA | NA |
| Disaster Medicine and Public Health Preparedness | 0.977 | 41 | United Kingdom | Cambridge University Press | 2007-2020 | 77 | 0%  (0) | 34.6%  (9) | 21.1%  (16) | NA | NA | 22%  (16) | 1%  (1) | NA | 6.5%  (5) | 1.3%  (1) | NA | 92.2%  (71) | NA | 94.8%  (73) | 2.6%  (2) | 2.6%  (2) | NA | NA |
| Diving and Hyperbaric Medicine | 1.5 | 20 | Australia | South Pacific Underwater Medicine Society | 2006-2020 | 15 | 0%  (0) | 0%  (0) | 26.7%  (4) | NA | NA | 26%  (4) | 0%  (0) | NA | NA | 6.7%  (1) | NA | 93.3%  (14) | NA | 100%  (15) | NA | NA | NA | NA |
| Drug Safety | 3.442 | 124 | United Kingdom | Adis International Ltd | 1990-2020 | 49 | 0%  (0) | 0%  (0) | 10.6%  (5) | NA | NA | 10%  (5) | 4%  (2) | 2%  (1) | 6.1%  (3) | NA | NA | 91.8%  (45) | NA | 93.9%  (46) | 2%  (1) | 4.1%  (2) | NA | NA |
| Eastern Mediterranean Health Journal | 0.678 | 47 | Switzerland | World Health Organization | 1996-2020 | 22 | 0%  (0) | 0%  (0) | 22.2%  (2) | 8.3%  (1) | NA | 14%  (3) | 5%  (1) | 31.8%  (7) | 36.4%  (8) | NA | 4.5%  (1) | 22.7%  (5) | 4.5%  (1) | 36.4%  (8) | 9.1%  (2) | 45.5%  (10) | 4.5%  (1) | 4.5%  (1) |
| Economics & Human Biology | 1.876 | 57 | Netherlands | Elsevier | 2003-2020 | 37 | 66.7%  (2) | 42.9%  (3) | 16.2%  (6) | NA | NA | 16%  (6) | 0%  (0) | NA | 2.7%  (1) | NA | NA | 97.3%  (36) | NA | 100%  (37) | NA | NA | NA | NA |
| Environmental Health and Preventive Medicine | 2.71 | 40 | Japan | Springer Japan | 1996-2020 | 45 | 0%  (0) | 0%  (0) | 13.6%  (6) | NA | NA | 14%  (6) | 2%  (1) | NA | 73.3%  (33) | 2.2%  (1) | NA | 24.4%  (11) | NA | 73.3%  (33) | 20%  (9) | 6.7%  (3) | NA | NA |
| Epidemiologia & Prevenzione | 1.071 | 30 | Italy | Inferenze Scarl | 1987-2020 | 54 | NA | 59.3%  (16) | 46.3%  (25) | NA | NA | 46%  (25) | 0%  (0) | NA | NA | NA | NA | 100%  (54) | NA | 100%  (54) | NA | NA | NA | NA |
| Epidemiologic Reviews | 8.16 | 106 | United Kingdom | Oxford University Press | 1979-2019 | 15 | 0%  (0) | 0%  (0) | 26.7%  (4) | NA | NA | 26%  (4) | 0%  (0) | NA | NA | NA | NA | 100%  (15) | NA | 100%  (15) | NA | NA | NA | NA |
| Epidemiology | 5.071 | 173 | United States | Lippincott Williams and Wilkins Ltd. | 1990-2020 | 36 | 0%  (0) | 33.3%  (2) | 33.3%  (12) | NA | NA | 34%  (12) | 0%  (0) | NA | 2.8%  (1) | NA | NA | 97.2%  (35) | NA | 97.2%  (35) | 2.8%  (1) | NA | NA | NA |
| Epidemiology and Infection | 2.152 | 109 | United Kingdom | Cambridge University Press | 1970, 1987-2020 | 49 | 0%  (0) | 33.3%  (5) | 28.6%  (14) | NA | NA | 28%  (14) | 0%  (0) | 4.1%  (2) | 6.1%  (3) | NA | 6.1%  (3) | 83.7%  (41) | NA | 83.7%  (41) | 8.2%  (4) | 6.1%  (3) | 2%  (1) | NA |
| Ethiopian Journal of Health Development | 0.469 | 19 | Ethiopia | Ethiopian Public Health Association | 2010-2019 | 10 | NA | NA | 30%  (3) | NA | NA | 30%  (3) | 0%  (0) | 90%  (9) | NA | NA | NA | 10%  (1) | NA | 10%  (1) | NA | NA | 90%  (9) | NA |
| Ethnicity & Disease | 1.228 | 67 | United States | ISHIB | 1991-1994, 1996-2020 | 27 | 0%  (0) | 33.3%  (4) | 44.4%  (12) | NA | NA | 44%  (12) | 0%  (0) | NA | NA | NA | NA | 100%  (27) | NA | 100%  (27) | NA | NA | NA | NA |
| Ethnicity & Health | 2.554 | 57 | United Kingdom | Routledge | 1996-2020 | 51 | 100%  (1) | 76.9%  (20) | 68.6%  (35) | NA | NA | 68%  (35) | 0%  (0) | 3.9%  (2) | 5.9%  (3) | NA | 3.9%  (2) | 86.3%  (44) | NA | 88.2%  (45) | 9.8%  (5) | 2%  (1) | NA | NA |
| European Journal of Contraception and Reproductive Health Care | 1.575 | 46 | United Kingdom | Informa Healthcare | 1996-2020 | 22 | 0%  (0) | 10%  (1) | 18.2%  (4) | NA | NA | 18%  (4) | 0%  (0) | NA | 4.5%  (1) | NA | 9.1%  (2) | 86.4%  (19) | NA | 86.4%  (19) | 13.6%  (3) | NA | NA | NA |
| European Journal of Epidemiology | 7.135 | 111 | Netherlands | Springer Netherlands | 1985-2001, 2003-2020 | 63 | 0%  (0) | 41.7%  (10) | 22.2%  (14) | NA | NA | 22%  (14) | 0%  (0) | NA | 1.6%  (1) | NA | NA | 98.4%  (62) | NA | 98.4%  (62) | NA | 1.6%  (1) | NA | NA |
| European Journal of Public Health | 2.391 | 91 | United Kingdom | Oxford University Press | 1991-2020 | 28 | 0%  (0) | 25%  (1) | 32.1%  (9) | NA | NA | 32%  (9) | 0%  (0) | NA | NA | 14.3%  (4) | NA | 85.7%  (24) | NA | 96.4%  (27) | 3.6%  (1) | NA | NA | NA |
| Evolution Medicine and Public Health | 4.222 | 22 | United Kingdom | Oxford University Press | 2013-2020 | 54 | 0%  (0) | 20%  (1) | 18.5%  (10) | NA | NA | 18%  (10) | 0%  (0) | NA | 1.9%  (1) | 1.9%  (1) | NA | 96.3%  (52) | NA | 100%  (54) | NA | NA | NA | NA |
| Families Systems & Health | 1.289 | 47 | United States | American Psychological Association Inc. | 1996-2020 | 40 | 100%  (3) | 81.8%  (9) | 55%  (22) | NA | NA | 56%  (22) | 0%  (0) | NA | NA | NA | NA | 100%  (40) | NA | 100%  (40) | NA | NA | NA | NA |
| Family & Community Health | 0.897 | 46 | United States | Lippincott Williams and Wilkins Ltd. | 1978-2020 | 26 | 100%  (1) | 75%  (3) | 80.8%  (21) | NA | NA | 80%  (21) | 0%  (0) | NA | 3.8%  (1) | NA | NA | 96.2%  (25) | NA | 96.2%  (25) | 3.8%  (1) | NA | NA | NA |
| Fluoride | 1 | 46 | New Zealand | International Society for Fluoride Research | 1973-2020 | 16 | 0%  (0) | 0%  (0) | 18.8%  (3) | NA | NA | 18%  (3) | 0%  (0) | NA | 62.5%  (10) | 6.2%  (1) | 6.2%  (1) | 25%  (4) | NA | 37.5%  (6) | 25%  (4) | 37.5%  (6) | NA | NA |
| Frontiers in Public Health | 2.483 | 41 | Switzerland | Frontiers Media S.A. | 2013-2020 | 20 | 0%  (0) | 0%  (0) | 35%  (7) | NA | NA | 36%  (7) | 0%  (0) | NA | 10%  (2) | NA | NA | 90%  (18) | NA | 100%  (20) | NA | NA | NA | NA |
| Gaceta Sanitaria | 1.564 | 42 | Spain | Ediciones Doyma, S.L. | 1987-2020 | 13 | 0%  (0) | 33.3%  (1) | 46.2%  (6) | NA | NA | 46%  (6) | 0%  (0) | NA | NA | NA | 30.8%  (4) | 69.2%  (9) | NA | 76.9%  (10) | 23.1%  (3) | NA | NA | NA |
| Games for Health Journal | 1.859 | 31 | United States | Mary Ann Liebert Inc. | 2012-2020 | 56 | 100%  (1) | 85.7%  (6) | 42.9%  (24) | NA | NA | 42%  (24) | 0%  (0) | 1.8%  (1) | 14.3%  (8) | NA | 1.8%  (1) | 82.1%  (46) | NA | 91.1%  (51) | 8.9%  (5) | NA | NA | NA |
| Geospatial Health | 1.078 | 36 | Italy | University of Naples Federico II | 2006-2020 | 44 | 0%  (0) | 28.6%  (2) | 20.5%  (9) | NA | NA | 20%  (9) | 0%  (0) | 2.3%  (1) | 15.9%  (7) | 4.5%  (2) | 2.3%  (1) | 75%  (33) | NA | 77.3%  (34) | 18.2%  (8) | 4.5%  (2) | NA | NA |
| Gesundheitswesen | 0.796 | 43 | Germany | Georg Thieme Verlag | 1992-2020 | 61 | NA | 7.7%  (1) | 21.3%  (13) | NA | NA | 22%  (13) | 0%  (0) | NA | NA | NA | NA | 100%  (61) | NA | 100%  (61) | NA | NA | NA | NA |
| Global Health Action | 2.162 | 47 | United Kingdom | Taylor and Francis Ltd. | 2009-2020 | 16 | 0%  (0) | 75%  (3) | 68.8%  (11) | NA | NA | 68%  (11) | 0%  (0) | 6.2%  (1) | 6.2%  (1) | NA | NA | 87.5%  (14) | NA | 87.5%  (14) | NA | 6.2%  (1) | 6.2%  (1) | NA |
| Global Health Promotion | 1.188 | 31 | United Kingdom | SAGE Publications Ltd | 1993, 2006, 2008-2020 | 9 | 100%  (1) | 100%  (1) | 55.6%  (5) | NA | NA | 56%  (5) | 0%  (0) | 11.1%  (1) | 11.1%  (1) | NA | 11.1%  (1) | 66.7%  (6) | NA | 77.8%  (7) | 11.1%  (1) | 11.1%  (1) | NA | NA |
| Global Health-Science and Practice | 2.352 | 28 | United States | NA | 2013-2020 | 28 | 0%  (0) | 50%  (7) | 42.9%  (12) | NA | NA | 42%  (12) | 0%  (0) | 10.7%  (3) | 10.7%  (3) | NA | NA | 78.6%  (22) | NA | 78.6%  (22) | 3.6%  (1) | 10.7%  (3) | 7.1%  (2) | NA |
| Global Public Health | 1.791 | 43 | United Kingdom | Routledge | 2006-2020 | 57 | 0%  (0) | 0%  (0) | 50%  (6) | 37.8%  (17) | NA | 40%  (23) | 0%  (0) | 8.8%  (5) | 14%  (8) | NA | 19.3%  (11) | 57.9%  (33) | NA | 59.6%  (34) | 24.6%  (14) | 15.8%  (9) | NA | NA |
| Globalization and Health | 2.525 | 53 | United Kingdom | BioMed Central Ltd. | 2005-2020 | 46 | 0%  (0) | 60%  (12) | 52.2%  (24) | NA | NA | 52%  (24) | 0%  (0) | 6.5%  (3) | 4.3%  (2) | 2.2%  (1) | 2.2%  (1) | 84.8%  (39) | NA | 87%  (40) | 8.7%  (4) | 4.3%  (2) | NA | NA |
| Health | 1.449 | 53 | United Kingdom | SAGE Publications Ltd | 1946-1948, 1973-1976, 1997-2020 | 30 | 0%  (0) | 50%  (3) | 46.7%  (14) | NA | NA | 46%  (14) | 0%  (0) | NA | NA | NA | NA | 100%  (30) | NA | 100%  (30) | NA | NA | NA | NA |
| Health & Place | 3.29 | 109 | United Kingdom | Elsevier Ltd. | 1995-2020 | 52 | 0%  (0) | 50%  (7) | 50%  (26) | NA | NA | 50%  (26) | 0%  (0) | NA | 3.8%  (2) | 1.9%  (1) | NA | 94.2%  (49) | NA | 100%  (52) | NA | NA | NA | NA |
| Health & Social Care in the Community | 2.05 | 68 | United Kingdom | Wiley-Blackwell Publishing Ltd | 1993-2020 | 27 | 100%  (1) | 60%  (3) | 65.4%  (17) | NA | NA | 66%  (17) | 4%  (1) | NA | 11.1%  (3) | NA | NA | 88.9%  (24) | NA | 100%  (27) | NA | NA | NA | NA |
| Health and Human Rights | 1.172 | 30 | United States | Harvard School of Public Health | 1994-2001, 2003-2004, 2006, 2008-2020 | 55 | 0%  (0) | 50%  (4) | 36.4%  (20) | NA | NA | 36%  (20) | 0%  (0) | 1.8%  (1) | NA | NA | 5.5%  (3) | 92.7%  (51) | NA | 92.7%  (51) | 5.5%  (3) | NA | 1.8%  (1) | NA |
| Health Care for Women International | 0.97 | 52 | United Kingdom | Taylor and Francis Ltd. | 1984-2020 | 23 | 100%  (1) | 100%  (1) | 100%  (1) | 100%  (22) | NA | 100%  (23) | 0%  (0) | 8.7%  (2) | 17.4%  (4) | NA | NA | 73.9%  (17) | NA | 78.3%  (18) | 13%  (3) | 8.7%  (2) | NA | NA |
| Health Education & Behavior | 1.927 | 92 | United States | SAGE Publications Inc. | 1957-1970, 1972-1978, 1980-2020 | 27 | 0%  (0) | 33.3%  (2) | 55.6%  (15) | NA | NA | 56%  (15) | 0%  (0) | NA | 3.7%  (1) | NA | 3.7%  (1) | 92.6%  (25) | NA | 92.6%  (25) | 7.4%  (2) | NA | NA | NA |
| Health Education Journal | 0.938 | 34 | United Kingdom | SAGE Publications Ltd | 1943-2020 | 32 | 0%  (0) | 80%  (4) | 62.5%  (20) | NA | NA | 62%  (20) | 0%  (0) | 3.1%  (1) | NA | NA | NA | 96.9%  (31) | NA | 96.9%  (31) | 3.1%  (1) | NA | NA | NA |
| Health Education Research | 1.108 | 103 | United Kingdom | Oxford University Press | 1986-2020 | 26 | 0%  (0) | 33.3%  (1) | 52%  (13) | NA | NA | 52%  (13) | 4%  (1) | 7.7%  (2) | 15.4%  (4) | NA | NA | 76.9%  (20) | NA | 84.6%  (22) | 7.7%  (2) | 7.7%  (2) | NA | NA |
| Health Expectations | 3.008 | 74 | United Kingdom | Wiley-Blackwell Publishing Ltd | 1999-2020 | 44 | 100%  (1) | 80%  (4) | 69.8%  (30) | NA | 60%  (3) | 70%  (30) | 2%  (1) | 2.3%  (1) | 4.5%  (2) | 2.3%  (1) | NA | 90.9%  (40) | NA | 93.2%  (41) | 4.5%  (2) | 2.3%  (1) | NA | NA |
| Health Promotion and Chronic Disease Prevention in Canada-Research Policy and Practice | 1.869 | 19 | Canada | Public Health Agency of Canada | 2015-2020 | 20 | 0%  (0) | 12.5%  (1) | 31.6%  (6) | NA | NA | 32%  (6) | 5%  (1) | NA | NA | NA | NA | 100%  (20) | NA | 100%  (20) | NA | NA | NA | NA |
| Health Promotion International | 1.98 | 84 | United Kingdom | Oxford University Press | 1986-2020 | 21 | 100%  (1) | 62.5%  (5) | 61.9%  (13) | NA | NA | 62%  (13) | 0%  (0) | 4.8%  (1) | 9.5%  (2) | NA | 4.8%  (1) | 81%  (17) | NA | 81%  (17) | 9.5%  (2) | 9.5%  (2) | NA | NA |
| Health Promotion Journal of Australia | 1.476 | 32 | Australia | John Wiley &amp; Sons Inc. | 2005-2020 | 48 | 0%  (0) | 75%  (3) | 78.9%  (15) | 69.2%  (18) | NA | 74%  (33) | 6%  (3) | NA | 4.2%  (2) | NA | NA | 95.8%  (46) | NA | 97.9%  (47) | 2.1%  (1) | NA | NA | NA |
| Health Reports | 3.358 | 60 | Canada | Statistics Canada | 1989-2020 | 11 | 0%  (0) | 50%  (2) | 45.5%  (5) | NA | NA | 46%  (5) | 0%  (0) | NA | NA | NA | NA | 100%  (11) | NA | 100%  (11) | NA | NA | NA | NA |
| Health Risk & Society | 1.047 | 49 | United Kingdom | Routledge | 1999-2020 | 31 | 0%  (0) | 0%  (0) | 31.6%  (6) | 33.3%  (4) | NA | 32%  (10) | 0%  (0) | 3.2%  (1) | NA | 3.2%  (1) | NA | 93.5%  (29) | NA | 96.8%  (30) | NA | 3.2%  (1) | NA | NA |
| Health Security | 1.297 | 37 | United States | Mary Ann Liebert Inc. | 2015-2020 | 72 | 0%  (0) | 69.2%  (18) | 38.9%  (28) | NA | NA | 38%  (28) | 0%  (0) | 2.8%  (2) | 9.7%  (7) | NA | NA | 87.5%  (63) | NA | 95.8%  (69) | NA | 4.2%  (3) | NA | NA |
| Health Systems & Reform | 1.98 | 17 | United States | Taylor and Francis Ltd. | 2015-2020 | 64 | 0%  (0) | 37.5%  (3) | 34.4%  (22) | NA | NA | 34%  (22) | 0%  (0) | 12.5%  (8) | 14.1%  (9) | NA | 4.7%  (3) | 68.8%  (44) | NA | 75%  (48) | 15.6%  (10) | 9.4%  (6) | NA | NA |
| Herd-Health Environments Research & Design Journal | 1.724 | 27 | United States | SAGE Publications Inc. | 2007-2020 | 31 | 100%  (2) | 100%  (2) | 68.4%  (13) | 63.6%  (7) | NA | 66%  (20) | 3%  (1) | NA | NA | NA | NA | 100%  (31) | NA | 100%  (31) | NA | NA | NA | NA |
| High Altitude Medicine & Biology | 1.495 | 52 | United States | Mary Ann Liebert Inc. | 2000-2020 | 60 | 0%  (0) | 0%  (0) | 17.2%  (10) | NA | NA | 18%  (10) | 3%  (2) | NA | 10%  (6) | 1.7%  (1) | 10%  (6) | 78.3%  (47) | NA | 83.3%  (50) | 8.3%  (5) | 8.3%  (5) | NA | NA |
| Indoor Air | 4.739 | 99 | Denmark | Blackwell Munksgaard | 1991-2020 | 48 | 0%  (0) | 28.6%  (4) | 25%  (12) | NA | NA | 24%  (12) | 0%  (0) | NA | 29.2%  (14) | NA | NA | 70.8%  (34) | NA | 87.5%  (42) | 12.5%  (6) | NA | NA | NA |
| Indoor and Built Environment | 1.9 | 44 | United Kingdom | SAGE Publications Ltd | 1992-2020 | 54 | 0%  (0) | 31.6%  (6) | 26.9%  (14) | NA | NA | 26%  (14) | 4%  (2) | NA | 44.4%  (24) | NA | NA | 55.6%  (30) | NA | 77.8%  (42) | 22.2%  (12) | NA | NA | NA |
| Infection Control and Hospital Epidemiology | 2.938 | 138 | United Kingdom | Cambridge University Press | 1988-2020 | 67 | 100%  (1) | 100%  (1) | 50%  (3) | 25%  (15) | NA | 28%  (18) | 1%  (1) | NA | 3%  (2) | NA | NA | 97%  (65) | NA | 98.5%  (66) | 1.5%  (1) | NA | NA | NA |
| Injury Prevention | 2.191 | 83 | United Kingdom | BMJ Publishing Group | 1995-2020 | 45 | 0%  (0) | 42.9%  (3) | 44.4%  (20) | NA | NA | 44%  (20) | 0%  (0) | 6.7%  (3) | 11.1%  (5) | NA | 2.2%  (1) | 80%  (36) | NA | 84.4%  (38) | 11.1%  (5) | 4.4%  (2) | NA | NA |
| International Archives of Occupational and Environmental Health | 1.935 | 88 | Germany | Springer Verlag | 1975-2020 | 28 | 0%  (0) | 0%  (0) | 50%  (3) | 13.6%  (3) | NA | 22%  (6) | 0%  (0) | 3.6%  (1) | 14.3%  (4) | NA | NA | 82.1%  (23) | NA | 96.4%  (27) | 3.6%  (1) | NA | NA | NA |
| International Health | 1.664 | 31 | United Kingdom | Oxford University Press | 2009-2020 | 40 | 0%  (0) | 0%  (0) | 30%  (6) | 25%  (5) | NA | 28%  (11) | 0%  (0) | 15%  (6) | 15%  (6) | 2.5%  (1) | NA | 67.5%  (27) | NA | 72.5%  (29) | 15%  (6) | 10%  (4) | 2.5%  (1) | NA |
| International Journal for Equity in Health | 2.595 | 55 | United Kingdom | BioMed Central Ltd. | 2002-2020 | 44 | 50%  (1) | 50%  (7) | 31.8%  (14) | NA | NA | 32%  (14) | 0%  (0) | 9.1%  (4) | 6.8%  (3) | 2.3%  (1) | 9.1%  (4) | 72.7%  (32) | NA | 75%  (33) | 20.5%  (9) | 2.3%  (1) | 2.3%  (1) | NA |
| International Journal of Circumpolar Health | 1.217 | 43 | United Kingdom | Taylor and Francis Ltd. | 1997-2020 | 9 | 100%  (1) | 66.7%  (2) | 55.6%  (5) | NA | NA | 56%  (5) | 0%  (0) | NA | NA | 11.1%  (1) | NA | 88.9%  (8) | NA | 88.9%  (8) | 11.1%  (1) | NA | NA | NA |
| International Journal of Epidemiology | 7.707 | 208 | United Kingdom | Oxford University Press | 1972-2020 | 56 | 0%  (0) | 80%  (4) | 28.6%  (16) | NA | NA | 28%  (16) | 0%  (0) | 7.1%  (4) | 14.3%  (8) | NA | NA | 78.6%  (44) | NA | 78.6%  (44) | 12.5%  (7) | 8.9%  (5) | NA | NA |
| International Journal of Health Geographics | 3.239 | 76 | United Kingdom | BioMed Central Ltd. | 2002-2020 | 30 | 0%  (0) | 0%  (0) | 13.3%  (4) | NA | NA | 14%  (4) | 0%  (0) | 3.3%  (1) | 10%  (3) | NA | 3.3%  (1) | 83.3%  (25) | NA | 83.3%  (25) | 13.3%  (4) | 3.3%  (1) | NA | NA |
| International Journal of Health Planning and Management | 1.367 | 41 | United Kingdom | John Wiley and Sons Ltd | 1985-2020 | 33 | 0%  (0) | 30%  (3) | 34.4%  (11) | NA | NA | 34%  (11) | 3%  (1) | 3%  (1) | 15.2%  (5) | 12.1%  (4) | 6.1%  (2) | 63.6%  (21) | NA | 60.6%  (20) | 30.3%  (10) | 6.1%  (2) | 3%  (1) | NA |
| International Journal of Hygiene and Environmental Health | 4.801 | 92 | Germany | Urban und Fischer Verlag Jena | 2000-2020 | 79 | 50%  (1) | 33.3%  (3) | 48.7%  (38) | NA | NA | 48%  (38) | 1%  (1) | 1.3%  (1) | 10.1%  (8) | NA | 1.3%  (1) | 87.3%  (69) | NA | 92.4%  (73) | 6.3%  (5) | 1.3%  (1) | NA | NA |
| International Journal of Injury Control and Safety Promotion | 1.342 | 35 | United Kingdom | Taylor and Francis Ltd. | 2005-2020 | 25 | 50%  (1) | 50%  (1) | 28%  (7) | NA | NA | 28%  (7) | 0%  (0) | 12%  (3) | 20%  (5) | NA | 8%  (2) | 60%  (15) | NA | 64%  (16) | 12%  (3) | 20%  (5) | 4%  (1) | NA |
| International Journal of Mental Health Promotion | 0.567 | 8 | United Kingdom | Taylor and Francis Ltd. | 2015-2020 | 23 | 100%  (1) | 100%  (1) | 33.3%  (7) | NA | NA | 34%  (7) | 9%  (2) | NA | 39.1%  (9) | 4.3%  (1) | NA | 56.5%  (13) | NA | 56.5%  (13) | 43.5%  (10) | NA | NA | NA |
| International Journal of Occupational Medicine and Environmental Health | 1.541 | 46 | Poland | Nofer Institute of Occupational Medicine | 1994-2020 | 46 | NA | NA | 37%  (17) | NA | NA | 36%  (17) | 0%  (0) | 2.2%  (1) | 10.9%  (5) | 21.7%  (10) | NA | 65.2%  (30) | NA | 89.1%  (41) | 8.7%  (4) | 2.2%  (1) | NA | NA |
| International Journal of Occupational Safety and Ergonomics | 1.601 | 34 | United Kingdom | Taylor and Francis Ltd. | 1995-2020 | 58 | 100%  (1) | 61.5%  (8) | 34.5%  (20) | NA | NA | 34%  (20) | 0%  (0) | NA | 8.6%  (5) | 51.7%  (30) | NA | 39.7%  (23) | NA | 96.6%  (56) | 1.7%  (1) | 1.7%  (1) | NA | NA |
| International Journal of Public Health | 2.419 | 65 | Switzerland | Birkhauser Verlag Basel | 1997, 2007-2020 | 53 | 33.3%  (1) | 36.4%  (4) | 40.4%  (21) | NA | NA | 40%  (21) | 2%  (1) | 7.5%  (4) | 5.7%  (3) | 5.7%  (3) | 5.7%  (3) | 75.5%  (40) | NA | 81.1%  (43) | 7.5%  (4) | 7.5%  (4) | 3.8%  (2) | NA |
| International Journal of Qualitative Studies on Health and Well-Being | 1.368 | 31 | United Kingdom | Taylor and Francis Ltd. | 2006-2020 | 25 | 100%  (1) | 100%  (4) | 64%  (16) | NA | NA | 64%  (16) | 0%  (0) | 16%  (4) | 12%  (3) | NA | 4%  (1) | 68%  (17) | NA | 68%  (17) | 28%  (7) | 4%  (1) | NA | NA |
| International Journal of Sexual Health | 1.191 | 38 | United States | Routledge | 2007-2020 | 61 | 0%  (0) | 50%  (1) | 50%  (1) | 44.8%  (26) | NA | 44%  (27) | 2%  (1) | NA | 4.9%  (3) | 1.6%  (1) | 4.9%  (3) | 88.5%  (54) | NA | 91.8%  (56) | 4.9%  (3) | 3.3%  (2) | NA | NA |
| International Journal of Technology Assessment in Health Care | 1.494 | 69 | United Kingdom | Cambridge University Press | 1970, 1978, 1985-2020 | 66 | 100%  (1) | 58.8%  (20) | 37.9%  (25) | NA | NA | 38%  (25) | 0%  (0) | 1.5%  (1) | 12.1%  (8) | 1.5%  (1) | 3%  (2) | 81.8%  (54) | NA | 86.4%  (57) | 6.1%  (4) | 7.6%  (5) | NA | NA |
| International Journal of Transgender Health | 3.369 | 37 | United States | Haworth Press Inc. | 1997-2002, 2005-2019 | 107 | 0%  (0) | 44.4%  (8) | 50.5%  (54) | NA | NA | 50%  (54) | 0%  (0) | NA | NA | NA | 3.7%  (4) | 96.3%  (103) | NA | 95.3%  (102) | 4.7%  (5) | NA | NA | NA |
| Iranian Journal of Public Health | 1.291 | 39 | Iran | Iranian Journal of Public Health | 1973-1980, 1987, 2004-2020 | 37 | 0%  (0) | 33.3%  (1) | 20%  (3) | 5.6%  (1) | NA | 12%  (4) | 11%  (4) | 2.7%  (1) | 64.9%  (24) | 2.7%  (1) | NA | 29.7%  (11) | NA | 45.9%  (17) | 2.7%  (1) | 51.4%  (19) | NA | NA |
| Israel Journal of Health Policy Research | 1.741 | 18 | United Kingdom | BioMed Central Ltd. | 2012-2020 | 36 | 0%  (0) | 0%  (0) | 27.8%  (10) | NA | NA | 28%  (10) | 0%  (0) | NA | NA | NA | NA | 100%  (36) | NA | 100%  (36) | NA | NA | NA | NA |
| Journal of Adolescent Health | 3.945 | 161 | United States | Elsevier USA | 1991-2020 | 97 | 100%  (1) | 80%  (4) | 59.8%  (58) | NA | NA | 60%  (58) | 0%  (0) | 5.2%  (5) | 3.1%  (3) | NA | NA | 91.8%  (89) | NA | 92.8%  (90) | 3.1%  (3) | 4.1%  (4) | NA | NA |
| Journal of Agromedicine | 1.221 | 30 | United States | Taylor and Francis Ltd. | 1994-2020 | 26 | 0%  (0) | 50%  (1) | 53.8%  (14) | NA | NA | 54%  (14) | 0%  (0) | NA | NA | NA | NA | 100%  (26) | NA | 100%  (26) | NA | NA | NA | NA |
| Journal of American College Health | 1.71 | 95 | United States | Routledge | 1982-2020 | 31 | 100%  (1) | 50%  (1) | 54.8%  (17) | NA | NA | 54%  (17) | 0%  (0) | NA | NA | NA | NA | 100%  (31) | NA | 100%  (31) | NA | NA | NA | NA |
| Journal of Arthropod-Borne Diseases | 1.11 | 21 | Iran | Tehran University of Medical Sciences | 2012-2020 | 21 | 0%  (0) | 0%  (0) | 9.1%  (1) | 22.2%  (2) | NA | 16%  (3) | 5%  (1) | NA | 61.9%  (13) | 4.8%  (1) | 4.8%  (1) | 28.6%  (6) | NA | 33.3%  (7) | 9.5%  (2) | 57.1%  (12) | NA | NA |
| Journal of Behavioral Health Services & Research | 1.59 | 55 | United States | Springer New York | 1996-2020 | 26 | 0%  (0) | 66.7%  (2) | 46.2%  (12) | NA | NA | 46%  (12) | 0%  (0) | NA | NA | NA | NA | 100%  (26) | NA | 100%  (26) | NA | NA | NA | NA |
| Journal of Cancer Education | 1.576 | 43 | United States | Springer Publishing Company | 1986-2020 | 81 | 100%  (1) | 40.9%  (9) | 58%  (47) | NA | NA | 58%  (47) | 0%  (0) | NA | NA | NA | NA | 100%  (81) | NA | 100%  (81) | NA | NA | NA | NA |
| Journal of Clinical Epidemiology | 4.952 | 212 | United States | Elsevier USA | 1988-2020 | 43 | 0%  (0) | 25%  (2) | 28.2%  (11) | NA | NA | 28%  (11) | 9%  (4) | 2.3%  (1) | 16.3%  (7) | NA | 7%  (3) | 74.4%  (32) | NA | 79.1%  (34) | 9.3%  (4) | 9.3%  (4) | 2.3%  (1) | NA |
| Journal of Community Health | 1.516 | 63 | Netherlands | Springer Netherlands | 1975-2020 | 20 | 0%  (0) | 0%  (0) | 45%  (9) | NA | NA | 44%  (9) | 0%  (0) | NA | NA | NA | NA | 100%  (20) | NA | 100%  (20) | NA | NA | NA | NA |
| Journal of Community Psychology | 1.106 | 25 | United States | SAGE Publications Inc. | 1994-2020 | 95 | 0%  (0) | 30%  (3) | 46.2%  (43) | NA | 78.6%  (22) | 46%  (43) | 2%  (2) | 4.2%  (4) | 9.5%  (9) | NA | 1.1%  (1) | 85.3%  (81) | NA | 85.3%  (81) | 9.5%  (9) | 4.2%  (4) | NA | 1.1%  (1) |
| Journal of Correctional Health Care | 1.058 | 25 | United States | SAGE Publications Inc. | 1994-2020 | 19 | 0%  (0) | 0%  (0) | 31.6%  (6) | NA | NA | 32%  (6) | 0%  (0) | NA | NA | NA | NA | 100%  (19) | NA | 100%  (19) | NA | NA | NA | NA |
| Journal of Developmental Origins of Health and Disease | 2.456 | 34 | United Kingdom | Cambridge University Press | 2010-2020 | 59 | 0%  (0) | 52.2%  (12) | 40.7%  (22) | 20%  (1) | NA | 38%  (23) | 0%  (0) | 1.7%  (1) | 6.8%  (4) | NA | 10.2%  (6) | 81.4%  (48) | NA | 88.1%  (52) | 10.2%  (6) | NA | 1.7%  (1) | NA |
| Journal of Epidemiology | 3.691 | 74 | Japan | Elsevier BV | 1991-2020 | 48 | 0%  (0) | 0%  (0) | 29.8%  (14) | NA | NA | 30%  (14) | 2%  (1) | NA | 97.9%  (47) | NA | NA | 2.1%  (1) | NA | 95.8%  (46) | 4.2%  (2) | NA | NA | NA |
| Journal of Epidemiology and Community Health | 3.342 | 170 | United Kingdom | BMJ Publishing Group | 1978-2020 | 45 | 0%  (0) | 0%  (0) | 28.6%  (6) | 16.7%  (4) | NA | 22%  (10) | 0%  (0) | 2.2%  (1) | 8.9%  (4) | NA | 6.7%  (3) | 82.2%  (37) | NA | 84.4%  (38) | 13.3%  (6) | 2.2%  (1) | NA | NA |
| Journal of Epidemiology and Global Health | 2.2 | 22 | France | Atlantis Press International | 2011-2020 | 37 | 0%  (0) | 8.3%  (1) | 14.7%  (5) | NA | NA | 14%  (5) | 8%  (3) | 2.7%  (1) | 43.2%  (16) | NA | NA | 54.1%  (20) | NA | 97.3%  (36) | NA | 2.7%  (1) | NA | NA |
| Journal of Global Health | 2.899 | 34 | United Kingdom | Edinburgh University Global Health Society | 2011-2020 | 60 | 33.3%  (1) | 26.3%  (5) | 36.7%  (22) | NA | NA | 36%  (22) | 0%  (0) | 5%  (3) | 15%  (9) | 6.7%  (4) | 5%  (3) | 68.3%  (41) | NA | 76.7%  (46) | 11.7%  (7) | 11.7%  (7) | NA | NA |
| Journal of Health and Social Behavior | 2.418 | 127 | United States | American Sociological Association | 1967-2020 | 51 | 100%  (1) | 55.6%  (5) | 64.7%  (33) | NA | NA | 64%  (33) | 0%  (0) | NA | NA | NA | NA | 100%  (51) | NA | 100%  (51) | NA | NA | NA | NA |
| Journal of Health Care for The Poor and Underserved | 1.08 | 59 | United States | Johns Hopkins University Press | 1990-2020 | 31 | 100%  (1) | 100%  (1) | 32.3%  (10) | NA | NA | 32%  (10) | 0%  (0) | NA | NA | NA | NA | 100%  (31) | NA | 100%  (31) | NA | NA | NA | NA |
| Journal of Hospital Infection | 3.271 | 118 | United Kingdom | W.B. Saunders Ltd | 1980-2020 | 113 | 25%  (1) | 14.3%  (1) | 43.4%  (46) | NA | NA | 44%  (46) | 6%  (7) | 0.9%  (1) | 11.5%  (13) | 2.7%  (3) | 0.9%  (1) | 84.1%  (95) | NA | 88.5%  (100) | 8.8%  (10) | 2.7%  (3) | NA | NA |
| Journal of Immigrant and Minority Health | 1.425 | 57 | United States | Springer New York | 2006-2020 | 22 | 100%  (1) | 57.1%  (4) | 54.5%  (12) | NA | NA | 54%  (12) | 0%  (0) | NA | NA | 4.5%  (1) | NA | 95.5%  (21) | NA | 95.5%  (21) | 4.5%  (1) | NA | NA | NA |
| Journal of Infection and Public Health | 2.447 | 35 | Netherlands | Elsevier BV | 2008-2020 | 49 | 100%  (1) | 40%  (4) | 36.8%  (14) | 45.5%  (5) | NA | 38%  (19) | 0%  (0) | 6.1%  (3) | 49%  (24) | NA | 4.1%  (2) | 40.8%  (20) | NA | 85.7%  (42) | 10.2%  (5) | 4.1%  (2) | NA | NA |
| Journal of Medical Screening | 1.897 | 64 | United Kingdom | SAGE Publications Ltd | 1994-2020 | 31 | 0%  (0) | 16.7%  (1) | 29%  (9) | NA | NA | 30%  (9) | 0%  (0) | NA | 6.5%  (2) | NA | NA | 93.5%  (29) | NA | 96.8%  (30) | NA | 3.2%  (1) | NA | NA |
| Journal of Men’s Health | 0.25 | 33 | Netherlands | Elsevier | 2008-2020 | 55 | 0%  (0) | 0%  (0) | 5.5%  (3) | NA | NA | 6%  (3) | 0%  (0) | NA | 49.1%  (27) | 5.5%  (3) | 1.8%  (1) | 43.6%  (24) | NA | 69.1%  (38) | 27.3%  (15) | 3.6%  (2) | NA | NA |
| Journal of Nepal Medical Association | 0.287 | 19 | Nepal | Nepal Medical Association | 1975, 1988, 1993-1994, 1996, 2005-2020 | 34 | 0%  (0) | 0%  (0) | 14.7%  (5) | NA | NA | 14%  (5) | 0%  (0) | NA | 55.9%  (19) | NA | NA | 44.1%  (15) | NA | 44.1%  (15) | NA | 55.9%  (19) | NA | NA |
| Journal of Occupational and Environmental Medicine | 1.642 | 110 | United States | Lippincott Williams and Wilkins Ltd. | 1959, 1963-1970, 1976-1978, 1980, 1988, 1990-1991, 1995-2020 | 21 | 0%  (0) | 0%  (0) | 25%  (5) | NA | NA | 24%  (5) | 5%  (1) | NA | 9.5%  (2) | NA | NA | 90.5%  (19) | NA | 95.2%  (20) | 4.8%  (1) | NA | NA | NA |
| Journal of Occupational Health | 2.289 | 59 | Japan | Japan Society for Occupational Health | 1996-2020 | 91 | 0%  (0) | 28.6%  (20) | 24.2%  (22) | NA | NA | 24%  (22) | 0%  (0) | NA | 82.4%  (75) | NA | 1.1%  (1) | 16.5%  (15) | NA | 93.4%  (85) | 4.4%  (4) | 2.2%  (2) | NA | NA |
| Journal of Occupational Health Psychology | 7.329 | 119 | United States | Educational Publishing Foundation | 1996-2020 | 101 | 100%  (1) | 85.7%  (6) | 49.5%  (50) | NA | NA | 50%  (50) | 0%  (0) | NA | 3%  (3) | NA | 2%  (2) | 95%  (96) | NA | 97%  (98) | 3%  (3) | NA | NA | NA |
| Journal of Occupational Medicine and Toxicology | 2.592 | 39 | United Kingdom | BioMed Central Ltd. | 2006-2020 | 46 | 0%  (0) | 10%  (1) | 19.6%  (9) | NA | NA | 20%  (9) | 0%  (0) | 2.2%  (1) | 8.7%  (4) | 8.7%  (4) | NA | 80.4%  (37) | NA | 95.7%  (44) | 2.2%  (1) | 2.2%  (1) | NA | NA |
| Journal of Palliative Care | 1.2 | 50 | United States | SAGE Publications Inc. | 1985-2015, 2017-2020 | 29 | 0%  (0) | 50%  (3) | 55.2%  (16) | NA | NA | 56%  (16) | 0%  (0) | NA | 6.9%  (2) | NA | NA | 93.1%  (27) | NA | 96.6%  (28) | 3.4%  (1) | NA | NA | NA |
| Journal of Physical Activity & Health | 1.993 | 73 | United States | Human Kinetics Publishers Inc. | 2004-2020 | 27 | 0%  (0) | 57.1%  (4) | 40.7%  (11) | NA | NA | 40%  (11) | 0%  (0) | 3.7%  (1) | 7.4%  (2) | NA | 7.4%  (2) | 81.5%  (22) | NA | 85.2%  (23) | 7.4%  (2) | 7.4%  (2) | NA | NA |
| Journal of Primary Prevention | 1.722 | 53 | United States | Kluwer Academic/Human Sciences Press Inc. | 1981-2020 | 48 | 0%  (0) | 77.8%  (7) | 50%  (24) | NA | NA | 50%  (24) | 0%  (0) | NA | NA | NA | NA | 100%  (48) | NA | 100%  (48) | NA | NA | NA | NA |
| Journal of Public Health | 1.806 | 82 | United Kingdom | Oxford University Press | 1980-2020 | 9 | 50%  (1) | 50%  (1) | 44.4%  (4) | NA | NA | 44%  (4) | 0%  (0) | NA | 11.1%  (1) | NA | NA | 88.9%  (8) | NA | 88.9%  (8) | NA | 11.1%  (1) | NA | NA |
| Journal of Public Health Dentistry | 1.743 | 63 | United States | Wiley-Blackwell | 1941-2020 | 12 | 0%  (0) | 0%  (0) | 58.3%  (7) | NA | NA | 58%  (7) | 0%  (0) | NA | NA | NA | NA | 100%  (12) | NA | 100%  (12) | NA | NA | NA | NA |
| Journal of Public Health Management and Practice | 1.49 | 50 | United States | Lippincott Williams and Wilkins Ltd. | 1995-2020 | 47 | 0%  (0) | 0%  (0) | 38.3%  (18) | NA | NA | 38%  (18) | 0%  (0) | NA | NA | NA | NA | 100%  (47) | NA | 100%  (47) | NA | NA | NA | NA |
| Journal of Public Health Policy | 1.5 | 50 | United Kingdom | Palgrave Macmillan Ltd. | 1980-2020 | 34 | 100%  (1) | 100%  (3) | 50%  (17) | NA | NA | 50%  (17) | 0%  (0) | 8.8%  (3) | 2.9%  (1) | NA | 5.9%  (2) | 82.4%  (28) | NA | 82.4%  (28) | 11.8%  (4) | 2.9%  (1) | 2.9%  (1) | NA |
| Journal of Racial and Ethnic Health Disparities | 1.661 | 21 | United States | Springer Nature | 2015-2020 | 41 | 0%  (0) | 20%  (1) | 40%  (16) | NA | 66.7%  (2) | 40%  (16) | 2%  (1) | NA | NA | NA | NA | 100%  (41) | NA | 100%  (41) | NA | NA | NA | NA |
| Journal of Religion & Health | 1.162 | 40 | United States | Kluwer Academic/Human Sciences Press Inc. | 1961-2020 | 33 | 0%  (0) | 0%  (0) | 24.2%  (8) | NA | NA | 24%  (8) | 0%  (0) | NA | NA | NA | NA | 100%  (33) | NA | 100%  (33) | NA | NA | NA | NA |
| Journal of Rural Health | 2.667 | 57 | United States | Wiley-Blackwell | 1985-2020 | 18 | 0%  (0) | 0%  (0) | 72.2%  (13) | NA | NA | 72%  (13) | 0%  (0) | NA | NA | NA | NA | 100%  (18) | NA | 100%  (18) | NA | NA | NA | NA |
| Journal of Safety Research | 2.861 | 85 | United Kingdom | Elsevier Ltd. | 1969-1980, 1982-2020 | 107 | 0%  (0) | 0%  (0) | 37.3%  (31) | NA | NA | 38%  (31) | 22%  (24) | NA | 3.7%  (4) | 0.9%  (1) | NA | 95.3%  (102) | NA | 97.2%  (104) | 0.9%  (1) | 1.9%  (2) | NA | NA |
| Journal of School Health | 1.673 | 86 | United States | Wiley-Blackwell | 1930-2020 | 20 | 0%  (0) | 0%  (0) | 50%  (1) | 61.1%  (11) | NA | 60%  (12) | 0%  (0) | 5%  (1) | NA | NA | NA | 85%  (17) | 10%  (2) | 85%  (17) | NA | 5%  (1) | NA | 10%  (2) |
| Journal of Transport & Health | 2.418 | 30 | Netherlands | Elsevier BV | 2013-2020 | 35 | 0%  (0) | 37.5%  (3) | 45.7%  (16) | NA | NA | 46%  (16) | 0%  (0) | NA | 5.7%  (2) | NA | 2.9%  (1) | 91.4%  (32) | NA | 97.1%  (34) | NA | 2.9%  (1) | NA | NA |
| Journal of Travel Medicine | 7.089 | 59 | United Kingdom | Oxford University Press | 1994-2020 | 87 | 100%  (1) | 28%  (7) | 22.7%  (17) | 50%  (4) | NA | 26%  (21) | 5%  (4) | 3.4%  (3) | 13.8%  (12) | NA | 2.3%  (2) | 80.5%  (70) | NA | 86.2%  (75) | 9.2%  (8) | 3.4%  (3) | NA | 1.1%  (1) |
| Journal of Tropical Medicine | 1.233 | 30 | Egypt | Hindawi Limited | 2010-2020 | 20 | NA | NA | 11.1%  (2) | NA | NA | 12%  (2) | 10%  (2) | 20%  (4) | 45%  (9) | NA | 5%  (1) | 30%  (6) | NA | 30%  (6) | 20%  (4) | 45%  (9) | 5%  (1) | NA |
| Journal of Urban Health-Bulletin of The New York Academy of Medicine | 2.356 | 92 | Germany | Springer Science + Business Media | 1988, 1998-2020 | 25 | 0%  (0) | 75%  (3) | 36%  (9) | NA | NA | 36%  (9) | 0%  (0) | 4%  (1) | 12%  (3) | NA | 4%  (1) | 80%  (20) | NA | 80%  (20) | 12%  (3) | 8%  (2) | NA | NA |
| Journal of Women's Health | 2.104 | 98 | United States | Mary Ann Liebert Inc. | 1997-2020 | 69 | 100%  (1) | 70.6%  (12) | 73.9%  (51) | NA | NA | 74%  (51) | 0%  (0) | NA | 1.4%  (1) | NA | NA | 98.6%  (68) | NA | 100%  (69) | NA | NA | NA | NA |
| Lancet Global Health | 21.597 | 88 | United Kingdom | Elsevier BV | 2013-2020 | 31 | 100%  (1) | 100%  (3) | 100%  (3) | 53.6%  (15) | NA | 58%  (18) | 0%  (0) | 19.4%  (6) | 12.9%  (4) | NA | 6.5%  (2) | 61.3%  (19) | NA | 64.5%  (20) | 12.9%  (4) | 16.1%  (5) | 6.5%  (2) | NA |
| Lancet Public Health | 16.292 | 45 | United Kingdom | Elsevier Ltd. | 2016-2020 | 24 | 100%  (1) | 50%  (1) | 50%  (1) | 54.5%  (12) | NA | 54%  (13) | 0%  (0) | 4.2%  (1) | 12.5%  (3) | NA | 4.2%  (1) | 79.2%  (19) | NA | 79.2%  (19) | 16.7%  (4) | 4.2%  (1) | NA | NA |
| LGBT Health | 3.272 | 32 | United States | Mary Ann Liebert Inc. | 2014-2020 | 49 | 0%  (0) | 28.6%  (2) | 42.9%  (21) | NA | NA | 42%  (21) | 0%  (0) | NA | 2%  (1) | NA | 2%  (1) | 95.9%  (47) | NA | 98%  (48) | 2%  (1) | NA | NA | NA |
| Longitudinal and Life Course Studies | 1 | 14 | United Kingdom | Society for Longitudinal and Life Course Studies | 2014-2020 | 36 | NA | NA | NA | 41.7%  (15) | NA | 42%  (15) | 0%  (0) | NA | NA | NA | NA | 100%  (36) | NA | 100%  (36) | NA | NA | NA | NA |
| Malawi Medical Journal | 0.598 | 18 | Malawi | Malawi Medical Journal | 1991-1993, 2008-2020 | 17 | 0%  (0) | 0%  (0) | 35.3%  (6) | NA | NA | 36%  (6) | 0%  (0) | 100%  (17) | NA | NA | NA | NA | NA | NA | NA | NA | 100%  (17) | NA |
| Maternal and Child Health Journal | 1.89 | 85 | United States | Springer GmbH &amp; Co, Auslieferungs-Gesellschaf | 1997-2020 | 70 | 0%  (0) | 0%  (0) | 71.4%  (50) | NA | NA | 72%  (50) | 0%  (0) | 8.6%  (6) | 7.1%  (5) | NA | 1.4%  (1) | 82.9%  (58) | NA | 85.7%  (60) | 4.3%  (3) | 7.1%  (5) | 2.9%  (2) | NA |
| Medical Anthropology Quarterly | 1.35 | 55 | United States | Wiley-Blackwell | 1983-2020 | 37 | 0%  (0) | 0%  (0) | 70.3%  (26) | NA | NA | 70%  (26) | 0%  (0) | NA | 2.7%  (1) | NA | NA | 97.3%  (36) | NA | 100%  (37) | NA | NA | NA | NA |
| Medical Care | 3.21 | 178 | United States | Lippincott Williams and Wilkins Ltd. | 1963-2020 | 28 | 50%  (1) | 26.3%  (5) | 39.3%  (11) | NA | NA | 40%  (11) | 0%  (0) | NA | NA | NA | NA | 100%  (28) | NA | 100%  (28) | NA | NA | NA | NA |
| Medicc Review | 0.644 | 21 | United States | MEDICC Medical Education Cooperation with Cuba | 2009-2020 | 49 | 0%  (0) | 66.7%  (2) | 30.6%  (15) | NA | NA | 30%  (15) | 0%  (0) | 4.1%  (2) | 4.1%  (2) | NA | 26.5%  (13) | 65.3%  (32) | NA | 65.3%  (32) | 28.6%  (14) | 6.1%  (3) | NA | NA |
| Medicina Del Lavoro | 0.978 | 25 | Italy | Mattioli 1885 S.p.A. | 1947-1963, 1965-2020 | 67 | 0%  (0) | 50%  (1) | 25%  (5) | 10.9%  (5) | NA | 16%  (10) | 1%  (1) | NA | 1.5%  (1) | NA | NA | 98.5%  (66) | NA | 98.5%  (66) | NA | 1.5%  (1) | NA | NA |
| Medycyna Pracy | 0.768 | 18 | Poland | Nofer Institute of Occupational Medicine | 1953-1955, 1961-1963, 1965-1967, 1973-2020 | 12 | 62.5%  (5) | 62.5%  (5) | 58.3%  (7) | NA | NA | 58%  (7) | 0%  (0) | NA | NA | 100%  (12) | NA | NA | NA | 100%  (12) | NA | NA | NA | NA |
| MMWR-Morbidity and Mortality Weekly Report | 13.606 | 100 | United States | Centers for Disease Control and Prevention  (CDC) | 2002-2020 | 17 | 0%  (0) | 0%  (0) | 35.3%  (6) | NA | NA | 36%  (6) | 0%  (0) | NA | NA | NA | NA | 100%  (17) | NA | 100%  (17) | NA | NA | NA | NA |
| Neuroepidemiology | 2.186 | 87 | Switzerland | S. Karger AG | 1982-2020 | 57 | 0%  (0) | 20%  (1) | 24.6%  (14) | NA | NA | 24%  (14) | 0%  (0) | NA | 19.3%  (11) | NA | 1.8%  (1) | 78.9%  (45) | NA | 87.7%  (50) | 5.3%  (3) | 7%  (4) | NA | NA |
| Nicotine & Tobacco Research | 4.079 | 113 | United Kingdom | Oxford University Press | 1999-2020 | 65 | 0%  (0) | 46.2%  (6) | 47.7%  (31) | NA | NA | 48%  (31) | 0%  (0) | NA | 1.5%  (1) | NA | NA | 98.5%  (64) | NA | 98.5%  (64) | NA | 1.5%  (1) | NA | NA |
| Noise & Health | 0.969 | 48 | India | Wolters Kluwer Medknow Publications | 2002-2020 | 28 | 0%  (0) | 0%  (0) | 28.6%  (8) | NA | NA | 28%  (8) | 0%  (0) | NA | 14.3%  (4) | 25%  (7) | 3.6%  (1) | 57.1%  (16) | NA | 67.9%  (19) | 21.4%  (6) | 10.7%  (3) | NA | NA |
| Occupational and Environmental Medicine | 3.824 | 141 | United Kingdom | BMJ Publishing Group | 1994-2020 | 25 | 0%  (0) | 33.3%  (5) | 32%  (8) | NA | NA | 32%  (8) | 0%  (0) | NA | 12%  (3) | NA | 4%  (1) | 84%  (21) | NA | 96%  (24) | 4%  (1) | NA | NA | NA |
| Occupational Medicine-Oxford | 1.569 | 85 | United Kingdom | Oxford University Press | 1948-1949, 1951-2020 | 27 | 0%  (0) | 0%  (0) | 36.4%  (4) | 16.7%  (2) | NA | 26%  (6) | 15%  (4) | 3.7%  (1) | 11.1%  (3) | NA | NA | 85.2%  (23) | NA | 88.9%  (24) | 11.1%  (3) | NA | NA | NA |
| One Health | 4.694 | 22 | Netherlands | Elsevier BV | 2015-2020 | 39 | 50%  (1) | 50%  (1) | 33.3%  (13) | NA | NA | 34%  (13) | 0%  (0) | 5.1%  (2) | 20.5%  (8) | NA | 2.6%  (1) | 71.8%  (28) | NA | 74.4%  (29) | 17.9%  (7) | 7.7%  (3) | NA | NA |
| Paediatric and Perinatal Epidemiology | 2.917 | 88 | United Kingdom | Wiley-Blackwell Publishing Ltd | 1987-2020 | 25 | 0%  (0) | 71.4%  (5) | 60%  (15) | NA | NA | 60%  (15) | 0%  (0) | NA | 4%  (1) | NA | 4%  (1) | 92%  (23) | NA | 92%  (23) | 8%  (2) | NA | NA | NA |
| Palliative Medicine | 3.739 | 106 | United Kingdom | SAGE Publications Ltd | 1987-2020 | 46 | 100%  (1) | 100%  (1) | 62.5%  (10) | 50%  (15) | NA | 54%  (25) | 0%  (0) | 2.2%  (1) | 8.7%  (4) | NA | NA | 89.1%  (41) | NA | 93.5%  (43) | 2.2%  (1) | 4.3%  (2) | NA | NA |
| Pathogens and Global Health | 2.42 | 71 | United Kingdom | Maney Publishing | 2012-2020 | 27 | 0%  (0) | 0%  (0) | 25.9%  (7) | NA | NA | 26%  (7) | 0%  (0) | NA | 14.8%  (4) | NA | 3.7%  (1) | 81.5%  (22) | NA | 88.9%  (24) | 7.4%  (2) | 3.7%  (1) | NA | NA |
| Patient Education and Counselling | 2.607 | 139 | Ireland | Elsevier Ireland Ltd | 1983-2020 | 39 | 0%  (0) | 57.1%  (8) | 63.2%  (24) | NA | NA | 64%  (24) | 3%  (1) | 2.6%  (1) | NA | NA | NA | 97.4%  (38) | NA | 97.4%  (38) | 2.6%  (1) | NA | NA | NA |
| Perspectives In Public Health | 4.073 | 32 | United Kingdom | SAGE Publications Ltd | 1951, 2009-2020 | 17 | NA | 0%  (0) | 60%  (6) | 42.9%  (3) | NA | 52%  (9) | 0%  (0) | NA | 5.9%  (1) | NA | NA | 94.1%  (16) | NA | 94.1%  (16) | 5.9%  (1) | NA | NA | NA |
| Pharmacoepidemiology and Drug Safety | 2.918 | 96 | United Kingdom | John Wiley and Sons Ltd | 1992-2020 | 83 | 0%  (0) | 47.5%  (19) | 40.5%  (32) | NA | NA | 40%  (32) | 5%  (4) | 1.2%  (1) | 21.7%  (18) | NA | NA | 77.1%  (64) | NA | 98.8%  (82) | 1.2%  (1) | NA | NA | NA |
| Population Health Metrics | 3.328 | 54 | United Kingdom | BioMed Central Ltd. | 2003-2020 | 32 | 0%  (0) | 27.3%  (3) | 25%  (8) | NA | NA | 24%  (8) | 0%  (0) | 6.2%  (2) | 15.6%  (5) | 3.1%  (1) | NA | 75%  (24) | NA | 81.2%  (26) | 12.5%  (4) | 6.2%  (2) | NA | NA |
| Prehospital Emergency Care | 2.29 | 63 | United Kingdom | Taylor and Francis Ltd. | 1997-2020 | 77 | 0%  (0) | 28.6%  (2) | 16.9%  (13) | NA | NA | 16%  (13) | 0%  (0) | NA | 1.3%  (1) | NA | NA | 98.7%  (76) | NA | 100%  (77) | NA | NA | NA | NA |
| Preventing Chronic Disease | 2.144 | 77 | United States | Centers for Disease Control and Prevention  (CDC) | 2004-2020 | 60 | 0%  (0) | 65.8%  (25) | 63.3%  (38) | NA | NA | 64%  (38) | 0%  (0) | NA | NA | NA | NA | 100%  (60) | NA | 100%  (60) | NA | NA | NA | NA |
| Prevention Science | 3.103 | 85 | United States | Springer New York | 2000-2020 | 55 | 100%  (1) | 42.9%  (3) | 54.5%  (30) | NA | 82.4%  (14) | 54%  (30) | 0%  (0) | NA | NA | NA | NA | 100%  (55) | NA | 100%  (55) | NA | NA | NA | NA |
| Preventive Medicine | 3.788 | 169 | United States | Academic Press Inc. | 1946, 1972-2020 | 46 | 0%  (0) | 33.3%  (2) | 34.8%  (16) | NA | NA | 34%  (16) | 0%  (0) | NA | NA | NA | NA | 100%  (46) | NA | 100%  (46) | NA | NA | NA | NA |
| Progress In Community Health Partnerships-Research Education and Action | 0.844 | 24 | United States | Johns Hopkins University Press | 2007-2020 | 13 | 0%  (0) | 0%  (0) | 76.9%  (10) | NA | NA | 76%  (10) | 0%  (0) | NA | NA | NA | NA | 100%  (13) | NA | 100%  (13) | NA | NA | NA | NA |
| Psychiatric Services | 2.539 | 145 | United States | American Psychiatric Association | 1995-2020 | 20 | 100%  (1) | 100%  (1) | 45%  (9) | NA | NA | 44%  (9) | 0%  (0) | NA | NA | NA | NA | 100%  (20) | NA | 100%  (20) | NA | NA | NA | NA |
| Psychology & Health | 2.528 | 90 | United Kingdom | Routledge | 1987-2020 | 75 | 50%  (1) | 53.8%  (7) | 54.7%  (41) | NA | NA | 54%  (41) | 0%  (0) | NA | 5.3%  (4) | NA | NA | 94.7%  (71) | NA | 98.7%  (74) | 1.3%  (1) | NA | NA | NA |
| Psychology Health & Medicine | 1.706 | 90 | United Kingdom | Routledge | 1987-2020 | 37 | 100%  (1) | 100%  (2) | 32.4%  (12) | NA | NA | 32%  (12) | 0%  (0) | 10.8%  (4) | 2.7%  (1) | NA | 2.7%  (1) | 83.8%  (31) | NA | 86.5%  (32) | 13.5%  (5) | NA | NA | NA |
| Public Health | 1.774 | 75 | Netherlands | Elsevier | 1888-1913, 1915-2020 | 30 | 50%  (1) | 42.9%  (3) | 30%  (9) | NA | NA | 30%  (9) | 0%  (0) | NA | 6.7%  (2) | NA | NA | 93.3%  (28) | NA | 96.7%  (29) | 3.3%  (1) | NA | NA | NA |
| Public Health Ethics | 1.176 | 22 | United Kingdom | Oxford University Press | 2009-2020 | 47 | 0%  (0) | 20%  (1) | 42.6%  (20) | NA | NA | 42%  (20) | 0%  (0) | 6.4%  (3) | 4.3%  (2) | NA | 2.1%  (1) | 87.2%  (41) | NA | 87.2%  (41) | 8.5%  (4) | 4.3%  (2) | NA | NA |
| Public Health Genomics | 1.518 | 51 | Switzerland | S. Karger AG | 2008-2020 | 37 | 50%  (1) | 33.3%  (1) | 33.3%  (12) | NA | NA | 34%  (12) | 3%  (1) | NA | 5.4%  (2) | 5.4%  (2) | 2.7%  (1) | 86.5%  (32) | NA | 91.9%  (34) | 5.4%  (2) | 2.7%  (1) | NA | NA |
| Public Health Nursing | 1.13 | 55 | United Kingdom | Wiley-Blackwell Publishing Ltd | 1945-1952, 1984-2020 | 18 | 100%  (1) | 100%  (2) | 83.3%  (15) | NA | NA | 84%  (15) | 0%  (0) | NA | NA | NA | NA | 100%  (18) | NA | 100%  (18) | NA | NA | NA | NA |
| Public Health Nutrition | 3.182 | 137 | United Kingdom | Cambridge University Press | 1998-2020 | 73 | 100%  (1) | 75%  (6) | 78.9%  (56) | NA | NA | 78%  (56) | 3%  (2) | 12.3%  (9) | 9.6%  (7) | NA | 11%  (8) | 67.1%  (49) | NA | 72.6%  (53) | 21.9%  (16) | 4.1%  (3) | 1.4%  (1) | NA |
| Public Health Reports | 1.764 | 92 | United States | SAGE Publications Inc. | 1945-1970, 1974-2020 | 37 | 100%  (1) | 33.3%  (2) | 40.5%  (15) | NA | NA | 40%  (15) | 0%  (0) | NA | NA | NA | NA | 100%  (37) | NA | 100%  (37) | NA | NA | NA | NA |
| Puerto Rico Health Sciences Journal | 0.435 | 25 | Puerto Rico | University of Puerto Rico Medical Sciences Campus | 1985-2020 | 27 | 100%  (1) | 100%  (1) | 53.8%  (7) | 35.7%  (5) | NA | 44%  (12) | 0%  (0) | NA | NA | NA | NA | 100%  (27) | NA | 100%  (27) | NA | NA | NA | NA |
| Quality of Life Research | 2.773 | 145 | Netherlands | Springer Netherlands | 1992-2020 | 61 | 50%  (1) | 50%  (1) | 44.4%  (8) | 27.9%  (12) | NA | 32%  (20) | 0%  (0) | NA | 4.9%  (3) | 3.3%  (2) | 1.6%  (1) | 90.2%  (55) | NA | 96.7%  (59) | 3.3%  (2) | NA | NA | NA |
| Reproductive Health | 2.177 | 52 | United Kingdom | BioMed Central Ltd. | 2004-2020 | 53 | 0%  (0) | 63%  (17) | 56%  (28) | 100%  (3) | NA | 58%  (31) | 0%  (0) | 18.9%  (10) | 18.9%  (10) | NA | 9.4%  (5) | 52.8%  (28) | NA | 52.8%  (28) | 22.6%  (12) | 20.8%  (11) | 3.8%  (2) | NA |
| Reproductive Health Matters | 1.662 | 0 | United Kingdom | Taylor and Francis Ltd. | 1993-2018 | 55 | 100%  (1) | 90%  (18) | 80%  (44) | NA | NA | 80%  (44) | 0%  (0) | 14.5%  (8) | 14.5%  (8) | 7.3%  (4) | 10.9%  (6) | 52.7%  (29) | NA | 60%  (33) | 16.4%  (9) | 20%  (11) | 3.6%  (2) | NA |
| Research In Social & Administrative Pharmacy | 2.844 | 47 | United States | Elsevier Inc. | 2005-2020 | 54 | 0%  (0) | 62.5%  (5) | 63%  (34) | NA | NA | 62%  (34) | 0%  (0) | NA | 9.3%  (5) | 1.9%  (1) | NA | 88.9%  (48) | NA | 92.6%  (50) | 7.4%  (4) | NA | NA | NA |
| Revista de Saude Publica | 1.748 | 77 | Brazil | University of Sao Paolo | 1967-2020 | 26 | 100%  (1) | 100%  (1) | 57.7%  (15) | NA | NA | 58%  (15) | 0%  (0) | NA | NA | NA | 96.2%  (25) | 3.8%  (1) | NA | 3.8%  (1) | 96.2%  (25) | NA | NA | NA |
| Revista Espanola De Salud Publica | 0.746 | 35 | Spain | Ministerio de Sanidad y Consumo | 1995-2020 | 65 | 100%  (1) | 80%  (8) | 43.1%  (28) | NA | NA | 44%  (28) | 0%  (0) | NA | NA | NA | NA | 100%  (65) | NA | 100%  (65) | NA | NA | NA | NA |
| Revista Panamericana de Salud Publica-Pan American Journal of Public Health | 0.703 | 57 | United States | Pan American Health Organization | 1997-2020 | 10 | 0%  (0) | 50%  (1) | 40%  (4) | NA | NA | 40%  (4) | 0%  (0) | NA | NA | NA | 70%  (7) | 30%  (3) | NA | 40%  (4) | 50%  (5) | NA | NA | 10%  (1) |
| Revue D Epidemiologie Et De Sante Publique | 1.398 | 39 | France | Elsevier Masson | 1976-2020 | 34 | 100%  (2) | 100%  (2) | 41.2%  (14) | NA | NA | 42%  (14) | 0%  (0) | 8.8%  (3) | NA | NA | NA | 91.2%  (31) | NA | 91.2%  (31) | NA | NA | 8.8%  (3) | NA |
| Risk Analysis | 3.137 | 130 | United Kingdom | Wiley-Blackwell Publishing Ltd | 1981-2020 | 53 | 0%  (0) | 25%  (3) | 30.2%  (16) | NA | NA | 30%  (16) | 0%  (0) | NA | NA | NA | NA | 100%  (53) | NA | 100%  (53) | NA | NA | NA | NA |
| Rural and Remote Health | 1.147 | 40 | Australia | James Cook University | 2005-2020 | 16 | 0%  (0) | 44.4%  (4) | 50%  (8) | NA | NA | 50%  (8) | 0%  (0) | 6.2%  (1) | 12.5%  (2) | NA | 6.2%  (1) | 75%  (12) | NA | 87.5%  (14) | 6.2%  (1) | 6.2%  (1) | NA | NA |
| Safety and Health At Work | 1.945 | 30 | South Korea | Elsevier BV | 2010-2020 | 78 | 50%  (1) | 14.3%  (2) | 25%  (19) | NA | NA | 24%  (19) | 3%  (2) | 1.3%  (1) | 56.4%  (44) | NA | 3.8%  (3) | 38.5%  (30) | NA | 91%  (71) | 6.4%  (5) | 2.6%  (2) | NA | NA |
| Sahara J-Journal of Social Aspects of HIV-AIDS | 1.3 | 29 | United Kingdom | Taylor and Francis Ltd. | 2004-2020 | 54 | 100%  (1) | 33.3%  (1) | 61.1%  (33) | NA | NA | 62%  (33) | 0%  (0) | 70.4%  (38) | NA | NA | NA | 29.6%  (16) | NA | 29.6%  (16) | 53.7%  (29) | 9.3%  (5) | 7.4%  (4) | NA |
| Salud Colectiva | 0.489 | 12 | Argentina | Universidad Nacional de Lanus | 2008-2020 | 40 | 0%  (0) | 42.9%  (9) | 45%  (18) | NA | NA | 44%  (18) | 0%  (0) | NA | NA | NA | 85%  (34) | 15%  (6) | NA | 15%  (6) | 85%  (34) | NA | NA | NA |
| Salud Publica De Mexico | 1.647 | 54 | Mexico | Instituto Nacional de Salud Publica | 1961-2020 | 33 | 0%  (0) | 33.3%  (2) | 18.2%  (6) | NA | NA | 18%  (6) | 0%  (0) | NA | NA | NA | 57.6%  (19) | 42.4%  (14) | NA | 45.5%  (15) | 54.5%  (18) | NA | NA | NA |
| Sante Publique | 0.248 | 17 | France | Societe Francaise de Sante Publique | 1947, 1973-1979, 1993, 1996-2020 | 43 | 0%  (0) | 75%  (3) | 46.5%  (20) | NA | NA | 46%  (20) | 0%  (0) | 16.3%  (7) | NA | NA | NA | 83.7%  (36) | NA | 83.7%  (36) | NA | 9.3%  (4) | 7%  (3) | NA |
| Saude e Sociedade | 0.322 | 20 | Brazil | University of Sao Paolo | 2008-2020 | 52 | 100%  (1) | 100%  (1) | 66.7%  (18) | 43.5%  (10) | NA | 56%  (28) | 4%  (2) | 1.9%  (1) | NA | NA | 84.6%  (44) | 13.5%  (7) | NA | 13.5%  (7) | 84.6%  (44) | NA | 1.9%  (1) | NA |
| Scandinavian Journal of Public Health | 2.183 | 82 | United Kingdom | SAGE Publications Ltd | 1973-1982, 1984-1986, 1988-2020 | 51 | 0%  (0) | 45.5%  (5) | 49%  (25) | NA | NA | 50%  (25) | 0%  (0) | NA | NA | NA | NA | 100%  (51) | NA | 100%  (51) | NA | NA | NA | NA |
| Scandinavian Journal of Work Environment & Health | 4.119 | 103 | Finland | Finnish Institute of Occupational Health | 1975-2020 | 90 | 0%  (0) | 0%  (0) | 29.4%  (5) | 35.6%  (26) | NA | 34%  (31) | 0%  (0) | NA | 2.2%  (2) | NA | NA | 97.8%  (88) | NA | 100%  (90) | NA | NA | NA | NA |
| Sex Education-Sexuality Society and Learning | 1.48 | 34 | United Kingdom | Routledge | 2005-2020 | 40 | 0%  (0) | 50%  (1) | 77.5%  (31) | NA | NA | 78%  (31) | 0%  (0) | 10%  (4) | 10%  (4) | NA | 5%  (2) | 75%  (30) | NA | 72.5%  (29) | 17.5%  (7) | 10%  (4) | NA | NA |
| Sexual & Reproductive Healthcare | 1.34 | 27 | Netherlands | Elsevier | 2010-2020 | 29 | 100%  (1) | 100%  (4) | 100%  (9) | 95%  (19) | NA | 96%  (28) | 0%  (0) | NA | 3.4%  (1) | NA | NA | 96.6%  (28) | NA | 96.6%  (28) | NA | 3.4%  (1) | NA | NA |
| Sexual and Reproductive Health Matters | Not Available | 57 | United Kingdom | Taylor and Francis Ltd. | 2019-2020 | 44 | 100%  (1) | 100%  (10) | 79.5%  (35) | NA | NA | 80%  (35) | 0%  (0) | 15.9%  (7) | 13.6%  (6) | 6.8%  (3) | 11.4%  (5) | 52.3%  (23) | NA | 61.4%  (27) | 15.9%  (7) | 15.9%  (7) | 6.8%  (3) | NA |
| Sexual Health | 1.584 | 43 | Australia | CSIRO | 2004-2020 | 54 | 0%  (0) | 0%  (0) | 32.1%  (17) | NA | NA | 32%  (17) | 2%  (1) | 1.9%  (1) | 25.9%  (14) | NA | NA | 72.2%  (39) | NA | 77.8%  (42) | 14.8%  (8) | 7.4%  (4) | NA | NA |
| Social Science & Medicine | 3.616 | 243 | United Kingdom | Elsevier Ltd. | 1967-2020 | 21 | 0%  (0) | 36.4%  (4) | 52.4%  (11) | NA | NA | 52%  (11) | 0%  (0) | NA | NA | NA | NA | 100%  (21) | NA | 100%  (21) | NA | NA | NA | NA |
| Social Work In Public Health | 0.607 | 28 | United States | Routledge | 2007-2020 | 16 | 100%  (1) | 100%  (1) | 62.5%  (10) | NA | NA | 62%  (10) | 0%  (0) | NA | NA | NA | NA | 100%  (16) | NA | 100%  (16) | NA | NA | NA | NA |
| Sociology of Health & Illness | 2.317 | 97 | United Kingdom | Wiley-Blackwell Publishing Ltd | 1979-2020 | 18 | 100%  (2) | 71.4%  (5) | 72.2%  (13) | NA | NA | 72%  (13) | 0%  (0) | NA | NA | NA | NA | 100%  (18) | NA | 100%  (18) | NA | NA | NA | NA |
| Statistics In Medicine | 1.783 | 183 | United Kingdom | John Wiley and Sons Ltd | 1982-2020 | 62 | 25%  (1) | 25%  (1) | 32.3%  (20) | NA | NA | 32%  (20) | 0%  (0) | NA | 3.2%  (2) | NA | NA | 96.8%  (60) | NA | 98.4%  (61) | 1.6%  (1) | NA | NA | NA |
| Studies In Family Planning | 2.667 | 68 | United Kingdom | Wiley-Blackwell Publishing Ltd | 1970-2020 | 26 | 0%  (0) | 0%  (0) | 75%  (12) | 50%  (5) | NA | 66%  (17) | 0%  (0) | 15.4%  (4) | 7.7%  (2) | NA | NA | 76.9%  (20) | NA | 76.9%  (20) | 7.7%  (2) | 15.4%  (4) | NA | NA |
| The Science of Diabetes Self-Management and Care | 2.087 | 74 | United States | SAGE Publications Inc. | 1979-2020 | 13 | 0%  (0) | 0%  (0) | 50%  (1) | 81.8%  (9) | NA | 76%  (10) | 0%  (0) | NA | NA | NA | NA | 100%  (13) | NA | 100%  (13) | NA | NA | NA | NA |
| The Southeast Asian Journal of Tropical Medicine and Public Health | 0.245 | 51 | Thailand | Southeast Asian Ministers of Education Organisation | 1971-2019 | 24 | 0%  (0) | 0%  (0) | 16.7%  (2) | 27.3%  (3) | NA | 22%  (5) | 4%  (1) | NA | 79.2%  (19) | NA | NA | 20.8%  (5) | NA | 29.2%  (7) | 50%  (12) | 20.8%  (5) | NA | NA |
| Tobacco Control | 6.726 | 122 | United Kingdom | BMJ Publishing Group | 1996-2020 | 60 | 100%  (1) | 73.3%  (11) | 51.2%  (21) | 36.8%  (7) | NA | 46%  (28) | 0%  (0) | 6.7%  (4) | 13.3%  (8) | NA | 6.7%  (4) | 73.3%  (44) | NA | 76.7%  (46) | 18.3%  (11) | 5%  (3) | NA | NA |
| Tobacco Induced Diseases | 1.434 | 27 | United Kingdom | BioMed Central Ltd. | 2009-2020 | 41 | 0%  (0) | 16.7%  (1) | 25%  (10) | NA | NA | 24%  (10) | 2%  (1) | 7.3%  (3) | 24.4%  (10) | 7.3%  (3) | NA | 61%  (25) | NA | 85.4%  (35) | 12.2%  (5) | 2.4%  (1) | NA | NA |
| Toxicology and Industrial Health | 1.708 | 57 | United Kingdom | SAGE Publications Ltd | 1985-2020 | 23 | 0%  (0) | 12.5%  (1) | 17.4%  (4) | NA | NA | 18%  (4) | 0%  (0) | NA | 13%  (3) | 4.3%  (1) | NA | 82.6%  (19) | NA | 87%  (20) | 13%  (3) | NA | NA | NA |
| Traffic Injury Prevention | 1.38 | 51 | United Kingdom | Taylor and Francis Ltd. | 2002-2020 | 15 | 0%  (0) | 0%  (0) | 33.3%  (5) | NA | NA | 34%  (5) | 0%  (0) | NA | NA | NA | NA | 100%  (15) | NA | 100%  (15) | NA | NA | NA | NA |
| Transactions of The Royal Society of Tropical Medicine and Hygiene | 1.868 | 105 | United Kingdom | Oxford University Press | 1907-2020 | 48 | 0%  (0) | 31.8%  (7) | 34.1%  (14) | 28.6%  (2) | NA | 34%  (16) | 0%  (0) | NA | 25%  (12) | 2.1%  (1) | 6.2%  (3) | 66.7%  (32) | NA | 72.9%  (35) | 12.5%  (6) | 12.5%  (6) | NA | 2.1%  (1) |
| Translational Behavioral Medicine | 2.864 | 39 | United States | Springer Publishing Company | 2011-2020 | 43 | 100%  (1) | 55.6%  (10) | 60.5%  (26) | NA | NA | 60%  (26) | 0%  (0) | NA | NA | NA | NA | 100%  (43) | NA | 100%  (43) | NA | NA | NA | NA |
| Travel Medicine and Infectious Disease | 4.589 | 48 | United States | Elsevier USA | 2003-2020 | 65 | 100%  (1) | 33.3%  (3) | 34.4%  (22) | NA | NA | 34%  (22) | 2%  (1) | 1.5%  (1) | 6.2%  (4) | 1.5%  (1) | 6.2%  (4) | 84.6%  (55) | NA | 84.6%  (55) | 13.8%  (9) | 1.5%  (1) | NA | NA |
| Tropical Doctor | 0.523 | 34 | United Kingdom | SAGE Publications Ltd | 1971-2020 | 7 | 0%  (0) | 25%  (1) | 50%  (3) | NA | NA | 50%  (3) | 14%  (1) | 42.9%  (3) | 14.3%  (1) | NA | NA | 42.9%  (3) | NA | 42.9%  (3) | NA | 28.6%  (2) | 28.6%  (2) | NA |
| Tropical Medicine and International Health | 2.308 | 114 | United Kingdom | Wiley-Blackwell Publishing Ltd | 1996-2020 | 50 | NA | 33.3%  (2) | 20%  (10) | NA | NA | 20%  (10) | 0%  (0) | 8%  (4) | 14%  (7) | NA | 4%  (2) | 74%  (37) | NA | 78%  (39) | 10%  (5) | 12%  (6) | NA | NA |
| Vector-Borne and Zoonotic Diseases | 2.249 | 73 | United States | Mary Ann Liebert Inc. | 2001-2020 | 37 | 0%  (0) | 0%  (0) | 13.5%  (5) | NA | NA | 14%  (5) | 0%  (0) | NA | 13.5%  (5) | 2.7%  (1) | 2.7%  (1) | 81.1%  (30) | NA | 89.2%  (33) | 10.8%  (4) | NA | NA | NA |
| Wilderness and Environmental Medicine | 1.426 | 43 | United States | Elsevier Ltd. | 1995-2020 | 11 | 0%  (0) | 0%  (0) | 18.2%  (2) | NA | NA | 18%  (2) | 0%  (0) | NA | 18.2%  (2) | NA | NA | 81.8%  (9) | NA | 81.8%  (9) | NA | 18.2%  (2) | NA | NA |
| Women & Health | 1.095 | 59 | United States | Routledge | 1975-2020 | 15 | 100%  (1) | 66.7%  (2) | 46.7%  (7) | NA | NA | 46%  (7) | 0%  (0) | NA | NA | NA | 80%  (12) | 20%  (3) | NA | 26.7%  (4) | 66.7%  (10) | NA | NA | 6.7%  (1) |
| Women’s Health Issues | 2.355 | 58 | United States | Elsevier USA | 1990-2020 | 21 | 100%  (1) | 83.3%  (5) | 95.2%  (20) | NA | NA | 96%  (20) | 0%  (0) | NA | NA | NA | NA | 100%  (21) | NA | 100%  (21) | NA | NA | NA | NA |
| WORK-A Journal of Prevention Assessment & Rehabilitation | 1.132 | 50 | Netherlands | IOS Press | NA | 50 | 100%  (1) | 100%  (1) | 58%  (29) | NA | NA | 58%  (29) | 0%  (0) | 4%  (2) | 6%  (3) | 2%  (1) | 6%  (3) | 82%  (41) | NA | 90%  (45) | 4%  (2) | 6%  (3) | NA | NA |
| Zdravstveno Varstvo | 1.097 | 10 | Germany | De Gruyter Open Ltd. | 2010-2020 | 31 | 0%  (0) | 0%  (0) | 48.4%  (15) | NA | NA | 48%  (15) | 0%  (0) | NA | NA | 61.3%  (19) | NA | 38.7%  (12) | NA | 100%  (31) | NA | NA | NA | NA |

**IF:** impact factor, **EiC**: editors-in-chief, **EL**: editorial leadership, **EB**: editorial board, **AB**: advisory board, **EC:** early career/young researchers, **Unkn:** unknown; **AF:** Africa, **AP:** Asia and Pacific,  **EE:** Eastern Europe, **LAC:** Latin America and the Caribbean, **WEO:** Western Europe and Other, **HIC:** high-income countries, **UMIC:** upper-middle-income countries, **LMIC:** lower-middle-income countries, **LIC:** low income countries
