# Supplementary material for: Challenging the “old boys club” in academia: Gender and geographic representation in editorial boards of journals publishing in environmental sciences and public health
Source: PLOS Glob Public Health. 2022 Jun 21;2(6):e0000541. doi: 10.1371/journal.pgph.0000541 (PMC10021803; doi:10.1371/journal.pgph.0000541)
Supplement: S4 Table — (DOCX) [file pgph.0000541.s005.docx]

## Supplement Table 4: Characteristics of journals categorised as Environmental Sciences and Environmental Studies following the JCR

| **Journal Title** | **IF** | **H** | **Country** | **Publisher** | **Coverage** | **Total** | **%**  **(N) Inferred women and gender minority** | | | | | | **%**  **(N) UN region of editors’ institutions** | | | | | | **%**  **(N) Income group of editors' institution** | | | | | |
| --- | --- | --- | --- | --- | --- | --- | --- | --- | --- | --- | --- | --- | --- | --- | --- | --- | --- | --- | --- | --- | --- | --- | --- | --- |
|  |  |  |  |  |  |  | **EiC** | **EL** | **EB** | **AB** | **EC** | **Tot** | **Unkn** | **AF** | **AP** | **EE** | **LAC** | **WEO** | **Unkn** | **HIC** | **UMIC** | **LMIC** | **LIC** | **Unkn** |
| Advances In Climate Change Research | 3.967 | 25 | China | Science Press | 2010-2020 | 100 | 0%  (0) | 0%  (0) | 14.1%  (14) | NA | 28.6%  (4) | 14%  (14) | 1%  (1) | NA | 60%  (60) | 1%  (1) | NA | 39%  (39) | NA | 45%  (45) | 53%  (53) | 2%  (2) | NA | NA |
| Aerobiologia | 2.708 | 49 | Netherlands | Springer Netherlands | 1985-2020 | 39 | 100%  (1) | 47.4%  (9) | 46.2%  (18) | NA | NA | 46%  (18) | 0%  (0) | NA | 7.7%  (3) | 10.3%  (4) | 2.6%  (1) | 79.5%  (31) | NA | 97.4%  (38) | 2.6%  (1) | NA | NA | NA |
| Aerosol Science and Technology | 2.34 | 109 | United Kingdom | Taylor and Francis Ltd. | 1982-2020 | 30 | NA | NA | 23.1%  (3) | 23.5%  (4) | NA | 24%  (7) | 0%  (0) | NA | 13.3%  (4) | NA | NA | 86.7%  (26) | NA | 93.3%  (28) | 6.7%  (2) | NA | NA | NA |
| African Journal of Range & Forage Science | 1.31 | 28 | United Kingdom | Taylor and Francis Ltd. | 1993-2020 | 38 | 0%  (0) | 0%  (0) | 34.6%  (9) | 16.7%  (2) | NA | 28%  (11) | 0%  (0) | 68.4%  (26) | 2.6%  (1) | NA | NA | 28.9%  (11) | NA | 28.9%  (11) | 63.2%  (24) | 2.6%  (1) | 5.3%  (2) | NA |
| Agriculture Ecosystems & Environment | 4.241 | 174 | Netherlands | Elsevier Ltd. | 1983-2020 | 57 | 0%  (0) | 0%  (0) | 27.3%  (3) | 26.2%  (11) | NA | 26%  (14) | 7%  (4) | 3.5%  (2) | 17.5%  (10) | NA | 7%  (4) | 71.9%  (41) | NA | 75.4%  (43) | 19.3%  (11) | 5.3%  (3) | NA | NA |
| Air Quality Atmosphere and Health | 2.87 | 45 | Netherlands | Springer Netherlands | 2008-2020 | 40 | 0%  (0) | 33.3%  (6) | 20.5%  (8) | NA | NA | 20%  (8) | 2%  (1) | NA | 32.5%  (13) | 2.5%  (1) | 2.5%  (1) | 62.5%  (25) | NA | 80%  (32) | 20%  (8) | NA | NA | NA |
| Ambio | 4.778 | 127 | Netherlands | Allen Press Inc. | 1973-2020 | 57 | 0%  (0) | 0%  (0) | 42.9%  (9) | 47.2%  (17) | NA | 46%  (26) | 0%  (0) | 5.3%  (3) | 14%  (8) | 1.8%  (1) | 7%  (4) | 71.9%  (41) | NA | 75.4%  (43) | 14%  (8) | 10.5%  (6) | NA | NA |
| Annali Di Botanica | 0.412 | 18 | Italy | Department of Environmental Biology, University La Sapienza of Rome | 1996-2000, 2002-2008, 2011-2020 | 37 | 0%  (0) | 60%  (3) | 21.6%  (8) | NA | NA | 22%  (8) | 0%  (0) | NA | NA | 2.7%  (1) | NA | 97.3%  (36) | NA | 100%  (37) | NA | NA | NA | NA |
| Annals of Regional Science | 1.75 | 62 | Germany | Springer Verlag | 1967-2020 | 38 | 33.3%  (1) | 33.3%  (1) | 36.8%  (14) | NA | NA | 36%  (14) | 0%  (0) | NA | 26.3%  (10) | NA | NA | 73.7%  (28) | NA | 97.4%  (37) | 2.6%  (1) | NA | NA | NA |
| Annual Review of Environment and Resources | 8.065 | 115 | United States | Annual Reviews Inc. | 2003-2019 | 9 | 0%  (0) | 0%  (0) | 33.3%  (3) | NA | NA | 34%  (3) | 0%  (0) | NA | 22.2%  (2) | 11.1%  (1) | NA | 66.7%  (6) | NA | 77.8%  (7) | 22.2%  (2) | NA | NA | NA |
| Annual Review of Resource Economics | 2.745 | 35 | United States | Annual Reviews Inc. | 2010-2019 | 10 | 0%  (0) | 0%  (0) | 20%  (2) | NA | NA | 20%  (2) | 0%  (0) | NA | NA | NA | NA | 100%  (10) | NA | 100%  (10) | NA | NA | NA | NA |
| Antarctic Science | 1.417 | 68 | United Kingdom | Cambridge University Press | 1986-1987, 1989-2020 | 20 | NA | NA | 0%  (0) | 23.5%  (4) | NA | 20%  (4) | 0%  (0) | 10%  (2) | NA | NA | 5%  (1) | 85%  (17) | NA | 85%  (17) | 15%  (3) | NA | NA | NA |
| Anthropocene | 3.39 | 33 | United Kingdom | Elsevier BV | 2013-2020 | 52 | 100%  (1) | 33.3%  (2) | 34.6%  (18) | NA | NA | 34%  (18) | 0%  (0) | 3.8%  (2) | 15.4%  (8) | 1.9%  (1) | 5.8%  (3) | 73.1%  (38) | NA | 80.8%  (42) | 17.3%  (9) | 1.9%  (1) | NA | NA |
| Anthropocene Review | 2.971 | 25 | United States | SAGE Publications Inc. | 2014-2020 | 61 | 0%  (0) | 0%  (0) | 30%  (3) | 25.5%  (13) | NA | 26%  (16) | 0%  (0) | 8.2%  (5) | 3.3%  (2) | NA | 4.9%  (3) | 83.6%  (51) | NA | 83.6%  (51) | 9.8%  (6) | 3.3%  (2) | 3.3%  (2) | NA |
| Applied Catalysis A-General | 5.006 | 214 | Netherlands | Elsevier | 1991-2020 | 22 | 0%  (0) | 11.1%  (1) | 19%  (4) | NA | NA | 20%  (4) | 5%  (1) | NA | 27.3%  (6) | 13.6%  (3) | 9.1%  (2) | 50%  (11) | NA | 77.3%  (17) | 22.7%  (5) | NA | NA | NA |
| Applied Ecology and Environmental Research | 0.712 | 32 | Hungary | Corvinus University of Budapest | 2003, 2005-2020 | 74 | 0%  (0) | 25%  (1) | 29.2%  (21) | NA | NA | 30%  (21) | 3%  (2) | 6.8%  (5) | 16.2%  (12) | 51.4%  (38) | 2.7%  (2) | 23%  (17) | NA | 66.2%  (49) | 23%  (17) | 9.5%  (7) | 1.4%  (1) | NA |
| Applied Spatial Analysis and Policy | 1.778 | 22 | Netherlands | Springer Netherlands | 2009-2020 | 29 | 33.3%  (1) | 33.3%  (1) | 28.6%  (2) | 0%  (0) | NA | 6%  (2) | 0%  (0) | NA | 20.7%  (6) | NA | 3.4%  (1) | 75.9%  (22) | NA | 82.8%  (24) | 17.2%  (5) | NA | NA | NA |
| Aquatic Conservation-Marine and Freshwater Ecosystems | 2.572 | 77 | United Kingdom | John Wiley and Sons Ltd | 1991-2020 | 45 | 0%  (0) | 33.3%  (2) | 15.9%  (7) | NA | NA | 16%  (7) | 2%  (1) | 4.4%  (2) | 11.1%  (5) | 2.2%  (1) | 6.7%  (3) | 75.6%  (34) | NA | 80%  (36) | 13.3%  (6) | 6.7%  (3) | NA | NA |
| Aquatic Ecosystem Health & Management | 0.761 | 41 | United Kingdom | Taylor and Francis Ltd. | 1998-2020 | 47 | 0%  (0) | 0%  (0) | 29.4%  (5) | 18.2%  (2) | NA | 24%  (7) | 40%  (19) | NA | 17%  (8) | 6.4%  (3) | 2.1%  (1) | 74.5%  (35) | NA | 83%  (39) | 14.9%  (7) | 2.1%  (1) | NA | NA |
| Aquatic Sciences | 2.402 | 73 | Switzerland | Birkhauser Verlag Basel | 1982, 1989-2020 | 27 | 0%  (0) | 0%  (0) | 26.9%  (7) | NA | NA | 26%  (7) | 4%  (1) | NA | 11.1%  (3) | 3.7%  (1) | NA | 85.2%  (23) | NA | 92.6%  (25) | 7.4%  (2) | NA | NA | NA |
| Archives of Environmental Contamination and Toxicology | 2.4 | 109 | United States | Springer New York | 1973-2020 | 30 | 0%  (0) | 0%  (0) | 31%  (9) | NA | NA | 32%  (9) | 3%  (1) | 3.3%  (1) | 16.7%  (5) | NA | 10%  (3) | 70%  (21) | NA | 73.3%  (22) | 23.3%  (7) | 3.3%  (1) | NA | NA |
| Archives of Environmental Protection | 1.775 | 21 | Poland | Polish Academy of Sciences | 2007-2020 | 30 | 100%  (1) | 100%  (1) | 23.1%  (6) | 33.3%  (1) | NA | 24%  (7) | 3%  (1) | NA | 10%  (3) | 53.3%  (16) | NA | 36.7%  (11) | NA | 96.7%  (29) | 3.3%  (1) | NA | NA | NA |
| Arctic | 1.54 | 59 | Canada | Arctic Institute of North America | 1973, 1975, 1977-1981, 1983-1984, 1986, 1988-2020 | 13 | 100%  (1) | 100%  (1) | 38.5%  (5) | NA | NA | 38%  (5) | 0%  (0) | NA | NA | NA | NA | 100%  (13) | NA | 100%  (13) | NA | NA | NA | NA |
| Arctic Antarctic and Alpine Research | 1.784 | 75 | United States | Institute of Arctic and Alpine Research | 1996-2020 | 22 | 50%  (1) | 50%  (1) | 28.6%  (6) | NA | NA | 28%  (6) | 5%  (1) | NA | 13.6%  (3) | NA | NA | 86.4%  (19) | NA | 86.4%  (19) | 13.6%  (3) | NA | NA | NA |
| Arctic Science | 1.64 | 4 | Canada | Canadian Science Publishing  (Ottawa, Canada) | 2019-2020 | 22 | 100%  (2) | 100%  (2) | 31.8%  (7) | NA | NA | 32%  (7) | 0%  (0) | NA | NA | NA | NA | 100%  (22) | NA | 100%  (22) | NA | NA | NA | NA |
| Arid Land Research and Management | 1.148 | 36 | United Kingdom | Taylor and Francis Ltd. | 2001-2020 | 46 | 0%  (0) | 0%  (0) | 0%  (0) | NA | NA | 0%  (0) | 20%  (9) | 10.9%  (5) | 4.3%  (2) | 4.3%  (2) | 2.2%  (1) | 78.3%  (36) | NA | 87%  (40) | 2.2%  (1) | 8.7%  (4) | 2.2%  (1) | NA |
| Atmospheric Chemistry and Physics | 5.414 | 206 | Germany | European Geosciences Union | 2001-2020 | 162 | 0%  (0) | 25%  (1) | 20.5%  (31) | 0%  (0) | NA | 20%  (31) | 4%  (7) | NA | 7.4%  (12) | NA | 0.6%  (1) | 92%  (149) | NA | 95.1%  (154) | 3.7%  (6) | 1.2%  (2) | NA | NA |
| Atmospheric Environment | 4.039 | 240 | United Kingdom | Elsevier Ltd. | 1968-1989, 1994-2021 | 37 | 0%  (0) | 0%  (0) | 27.3%  (3) | 29.2%  (7) | NA | 28%  (10) | 5%  (2) | NA | 37.8%  (14) | NA | NA | 62.2%  (23) | NA | 81.1%  (30) | 13.5%  (5) | 5.4%  (2) | NA | NA |
| Atmospheric Pollution Research | 3.527 | 45 | Netherlands | Turkish National Committee for Air Pollution Research  (TUNCAP) | 2010-2020 | 45 | 0%  (0) | 0%  (0) | 16.3%  (7) | NA | NA | 16%  (7) | 4%  (2) | NA | 13.3%  (6) | NA | 2.2%  (1) | 84.4%  (38) | NA | 64.4%  (29) | 35.6%  (16) | NA | NA | NA |
| Australasian Journal of Environmental Management | 1.157 | 25 | United Kingdom | Taylor and Francis Ltd. | 1996-2020 | 29 | 50%  (1) | 50%  (1) | 50%  (4) | 35%  (7) | NA | 40%  (11) | 3%  (1) | 3.4%  (1) | 3.4%  (1) | NA | NA | 93.1%  (27) | NA | 93.1%  (27) | 6.9%  (2) | NA | NA | NA |
| Biodiversity and Conservation | 2.935 | 131 | Netherlands | Springer Netherlands | 1992-2020 | 57 | 0%  (0) | 20%  (1) | 12.5%  (7) | NA | NA | 12%  (7) | 2%  (1) | 1.8%  (1) | 28.1%  (16) | NA | 7%  (4) | 63.2%  (36) | NA | 68.4%  (39) | 21.1%  (12) | 10.5%  (6) | NA | NA |
| Bioenergy Research | 2.195 | 58 | United States | Springer New York | 2009-2020 | 35 | 0%  (0) | 0%  (0) | 22.9%  (8) | NA | NA | 22%  (8) | 0%  (0) | NA | 5.7%  (2) | NA | 8.6%  (3) | 85.7%  (30) | NA | 88.6%  (31) | 11.4%  (4) | NA | NA | NA |
| Biogeochemistry | 4.161 | 146 | Netherlands | Springer Netherlands | 1984-2020 | 59 | 100%  (1) | 38.9%  (14) | 39.3%  (22) | NA | NA | 40%  (22) | 5%  (3) | NA | 3.4%  (2) | NA | NA | 96.6%  (57) | NA | 96.6%  (57) | 3.4%  (2) | NA | NA | NA |
| Biological Conservation | 4.711 | 199 | Netherlands | Elsevier BV | 1968-2020 | 42 | 0%  (0) | 54.5%  (6) | 35.7%  (15) | NA | NA | 36%  (15) | 0%  (0) | NA | 16.7%  (7) | 4.8%  (2) | 4.8%  (2) | 73.8%  (31) | NA | 83.3%  (35) | 14.3%  (6) | 2.4%  (1) | NA | NA |
| Biology and Environment-Proceedings of The Royal Irish Academy | 0.654 | 27 | Ireland | Royal Irish Academy | 1993, 1995-2020 | 13 | 0%  (0) | 0%  (0) | 23.1%  (3) | NA | NA | 24%  (3) | 0%  (0) | NA | NA | NA | NA | 100%  (13) | NA | 100%  (13) | NA | NA | NA | NA |
| Bioremediation Journal | 1.724 | 35 | United Kingdom | Taylor and Francis Ltd. | 1997-2020 | 33 | 100%  (1) | 40%  (2) | 18.8%  (6) | NA | NA | 18%  (6) | 3%  (1) | NA | 3%  (1) | 3%  (1) | 3%  (1) | 81.8%  (27) | 9.1%  (3) | 87.9%  (29) | 3%  (1) | NA | NA | 9.1%  (3) |
| Biotechnologie Agronomie Societe et Environnement | 1.319 | 36 | Belgium | Les Presses Agronomiques de Gembloux | 1997-2020 | 32 | 0%  (0) | 0%  (0) | 44.4%  (4) | 60.9%  (14) | NA | 56%  (18) | 0%  (0) | NA | 3.1%  (1) | NA | 12.5%  (4) | 81.2%  (26) | 3.1%  (1) | 81.2%  (26) | 12.5%  (4) | 3.1%  (1) | NA | 3.1%  (1) |
| Boreal Environment Research | 1.1 | 56 | Finland | Finnish Environment Institute | 1996-2020 | 23 | 0%  (0) | 27.3%  (3) | 17.4%  (4) | NA | NA | 18%  (4) | 0%  (0) | NA | NA | 4.3%  (1) | NA | 95.7%  (22) | NA | 100%  (23) | NA | NA | NA | NA |
| Bulletin of Environmental Contamination and Toxicology | 1.657 | 70 | United States | Springer New York | 1966-2020 | 195 | NA  (0) | 20.6%  (7) | 30.2%  (58) | NA | NA | 30%  (58) | 2%  (3) | 2.1%  (4) | 21%  (41) | 2.1%  (4) | 6.2%  (12) | 68.7%  (134) | NA | 72.3%  (141) | 24.6%  (48) | 3.1%  (6) | NA | NA |
| Business Strategy and The Environment | 5.483 | 105 | United Kingdom | John Wiley and Sons Ltd | 1992-2020 | 89 | 0%  (0) | 0%  (0) | 27%  (24) | NA | NA | 26%  (24) | 0%  (0) | NA | 14.6%  (13) | NA | 2.2%  (2) | 83.1%  (74) | NA | 86.5%  (77) | 11.2%  (10) | 2.2%  (2) | NA | NA |
| Carbon Balance and Management | 4.067 | 34 | United Kingdom | BioMed Central Ltd. | 2006-2020 | 30 | NA | NA | 10%  (3) | NA | NA | 10%  (3) | 0%  (0) | NA | 16.7%  (5) | 10%  (3) | NA | 73.3%  (22) | NA | 76.7%  (23) | 20%  (6) | 3.3%  (1) | NA | NA |
| Carbon Management | 1.667 | 34 | United Kingdom | Taylor and Francis Ltd. | 2010-2020 | 45 | 0%  (0) | 0%  (0) | 14.3%  (6) | NA | NA | 14%  (6) | 7%  (3) | NA | 6.7%  (3) | NA | 6.7%  (3) | 86.7%  (39) | NA | 88.9%  (40) | 6.7%  (3) | 4.4%  (2) | NA | NA |
| Carpathian Journal of Earth and Environmental Sciences | 1.307 | 23 | Romania | North University of Baia Mare | 2008-2020 | 49 | 0%  (0) | 16.7%  (1) | 13.5%  (5) | NA | NA | 14%  (5) | 24%  (12) | 2%  (1) | NA | 87.8%  (43) | NA | 10.2%  (5) | NA | 32.7%  (16) | 65.3%  (32) | 2%  (1) | NA | NA |
| Chemistry and Ecology | 1.4 | 37 | United Kingdom | Taylor and Francis Ltd. | 1982-1984, 1986-2001, 2003-2020 | 18 | 0%  (0) | 0%  (0) | 5.6%  (1) | NA | NA | 6%  (1) | 0%  (0) | 5.6%  (1) | 11.1%  (2) | NA | 16.7%  (3) | 66.7%  (12) | NA | 66.7%  (12) | 27.8%  (5) | 5.6%  (1) | NA | NA |
| Chemosphere | 5.778 | 248 | United Kingdom | Elsevier Ltd. | 1972-2021 | 104 | 33.3%  (1) | 33.3%  (11) | 28%  (28) | NA | NA | 28%  (28) | 4%  (4) | 1%  (1) | 38.5%  (40) | 3.8%  (4) | 2.9%  (3) | 53.8%  (56) | NA | 71.2%  (74) | 26.9%  (28) | 1.9%  (2) | NA | NA |
| Chinese Geographical Science | 1.854 | 36 | China | Science Press | 1991-2020 | 47 | 0%  (0) | 0%  (0) | 5%  (2) | 0%  (0) | NA | 4%  (2) | 11%  (5) | NA | 57.4%  (27) | 2.1%  (1) | NA | 40.4%  (19) | NA | 42.6%  (20) | 57.4%  (27) | NA | NA | NA |
| Clean Technologies and Environmental Policy | 2.429 | 55 | Germany | Springer Verlag | 2003-2020 | 47 | 0%  (0) | 20%  (1) | 31.8%  (14) | NA | NA | 32%  (14) | 6%  (3) | 2.1%  (1) | 23.4%  (11) | 10.6%  (5) | 8.5%  (4) | 55.3%  (26) | NA | 83%  (39) | 12.8%  (6) | 4.3%  (2) | NA | NA |
| Clean-Soil Air Water | 1.603 | 66 | Germany | Wiley-VCH Verlag | 2007-2020 | 35 | NA  (0) | NA  (0) | 33.3%  (4) | 13.6%  (3) | NA | 20%  (7) | 3%  (1) | NA | 20%  (7) | 5.7%  (2) | NA | 74.3%  (26) | NA | 80%  (28) | 11.4%  (4) | 8.6%  (3) | NA | NA |
| Climate and Development | 2.311 | 35 | United Kingdom | Taylor and Francis Ltd. | 2009-2020 | 35 | 50%  (1) | 36.4%  (4) | 40%  (14) | NA | NA | 40%  (14) | 0%  (0) | 11.4%  (4) | 11.4%  (4) | NA | 11.4%  (4) | 65.7%  (23) | NA | 71.4%  (25) | 11.4%  (4) | 14.3%  (5) | 2.9%  (1) | NA |
| Climate Change Economics | 1.271 | 26 | Singapore | World Scientific Publishing Co. Pte Ltd | 2010-2020 | 11 | 0%  (0) | 0%  (0) | 0%  (0) | NA | NA | 0%  (0) | 0%  (0) | 9.1%  (1) | 9.1%  (1) | NA | NA | 81.8%  (9) | NA | 90.9%  (10) | 9.1%  (1) | NA | NA | NA |
| Climate Policy | 4.011 | 66 | United Kingdom | Taylor and Francis Ltd. | 2001-2020 | 53 | 0%  (0) | 33.3%  (1) | 37.5%  (3) | 33.3%  (15) | NA | 34%  (18) | 0%  (0) | 7.5%  (4) | 28.3%  (15) | 7.5%  (4) | 9.4%  (5) | 47.2%  (25) | NA | 62.3%  (33) | 24.5%  (13) | 13.2%  (7) | NA | NA |
| Climate Research | 2.023 | 106 | Germany | Inter-Research | 1990-2020 | 36 | 0%  (0) | 0%  (0) | 8.8%  (3) | NA | NA | 8%  (3) | 6%  (2) | NA | 11.1%  (4) | 2.8%  (1) | 2.8%  (1) | 83.3%  (30) | NA | 91.7%  (33) | 8.3%  (3) | NA | NA | NA |
| Climate Risk Management | 4.904 | 30 | Netherlands | Elsevier BV | 2014-2020 | 26 | 25%  (1) | 25%  (1) | 25%  (1) | 27.3%  (6) | NA | 26%  (7) | 0%  (0) | 3.8%  (1) | 15.4%  (4) | NA | 3.8%  (1) | 76.9%  (20) | NA | 76.9%  (20) | 7.7%  (2) | 11.5%  (3) | 3.8%  (1) | NA |
| Climatic Change | 4.134 | 188 | Netherlands | Springer Netherlands | 1977-2020 | 91 | 0%  (0) | 0%  (0) | 38.5%  (35) | NA | NA | 38%  (35) | 0%  (0) | 2.2%  (2) | 12.1%  (11) | NA | 3.3%  (3) | 82.4%  (75) | NA | 86.8%  (79) | 9.9%  (9) | 2.2%  (2) | 1.1%  (1) | NA |
| Coastal Management | 1.547 | 49 | United Kingdom | Taylor and Francis Ltd. | 1987-2020 | 33 | 0%  (0) | 20%  (1) | 27.3%  (9) | NA | NA | 28%  (9) | 0%  (0) | NA | 6.1%  (2) | NA | 9.1%  (3) | 84.8%  (28) | NA | 87.9%  (29) | 6.1%  (2) | 6.1%  (2) | NA | NA |
| Computers Environment and Urban Systems | 4.655 | 92 | United Kingdom | Elsevier Ltd. | 1980-1986, 1988-2020 | 60 | 0%  (0) | 60%  (3) | 48.3%  (29) | NA | NA | 48%  (29) | 0%  (0) | NA | 8.3%  (5) | NA | 3.3%  (2) | 88.3%  (53) | NA | 91.7%  (55) | 8.3%  (5) | NA | NA | NA |
| Conservation & Society | 1.904 | 35 | India | Wolters Kluwer Medknow Publications | 2007-2020 | 65 | 0%  (0) | 50%  (4) | 57.4%  (27) | 27.8%  (5) | NA | 50%  (32) | 0%  (0) | 7.7%  (5) | 21.5%  (14) | 1.5%  (1) | 3.1%  (2) | 66.2%  (43) | NA | 70.8%  (46) | 12.3%  (8) | 16.9%  (11) | NA | NA |
| Conservation Biology | 5.405 | 222 | United Kingdom | Wiley-Blackwell Publishing Ltd | 1987-2020 | 96 | 0%  (0) | 40%  (4) | 41.5%  (39) | NA | NA | 42%  (39) | 2%  (2) | 4.2%  (4) | 15.6%  (15) | 1%  (1) | 5.2%  (5) | 74%  (71) | NA | 77.1%  (74) | 16.7%  (16) | 4.2%  (4) | 1%  (1) | 1%  (1) |
| Conservation Physiology | 2.57 | 37 | United States | Oxford University Press | 2013-2020 | 25 | 0%  (0) | 0%  (0) | 48%  (12) | NA | NA | 48%  (12) | 0%  (0) | 4%  (1) | NA | NA | 4%  (1) | 92%  (23) | NA | 92%  (23) | 8%  (2) | NA | NA | NA |
| Corporate Social Responsibility and Environmental Management | 4.542 | 73 | United Kingdom | John Wiley and Sons Ltd | 2003-2020 | 58 | 0%  (0) | 0%  (0) | 32.8%  (19) | NA | NA | 32%  (19) | 0%  (0) | NA | 25.9%  (15) | NA | NA | 74.1%  (43) | NA | 86.2%  (50) | 10.3%  (6) | 3.4%  (2) | NA | NA |
| Critical Reviews In Environmental Science and Technology | 8.302 | 107 | United Kingdom | Taylor and Francis Ltd. | 1993-2020 | 47 | 20%  (1) | 5%  (1) | 14.9%  (7) | NA | NA | 14%  (7) | 0%  (0) | NA | 27.7%  (13) | NA | NA | 72.3%  (34) | NA | 85.1%  (40) | 14.9%  (7) | NA | NA | NA |
| Cultural Geographies | 2.238 | 57 | United Kingdom | SAGE Publications Ltd | 1994-1998, 2002-2020 | 63 | 100%  (1) | 100%  (1) | 48.6%  (17) | 25%  (7) | NA | 38%  (24) | 0%  (0) | NA | 1.6%  (1) | 1.6%  (1) | NA | 96.8%  (61) | NA | 98.4%  (62) | 1.6%  (1) | NA | NA | NA |
| Current Opinion In Environmental Sustainability | 5.658 | 87 | Netherlands | Elsevier | 2009-2021 | 49 | 33.3%  (1) | 33.3%  (1) | 32.7%  (16) | NA | NA | 32%  (16) | 0%  (0) | 6.1%  (3) | 18.4%  (9) | NA | 4.1%  (2) | 71.4%  (35) | NA | 73.5%  (36) | 18.4%  (9) | 8.2%  (4) | NA | NA |
| Disasters | 1.937 | 70 | United Kingdom | Wiley-Blackwell Publishing Ltd | 1977-2020 | 25 | 50%  (2) | 50%  (2) | 45.8%  (11) | 0%  (0) | NA | 44%  (11) | 0%  (0) | NA | 4%  (1) | NA | 4%  (1) | 92%  (23) | NA | 88%  (22) | 12%  (3) | NA | NA | NA |
| Earths Future | 6.141 | 39 | United States | John Wiley and Sons Inc. | 2014-2020 | 11 | 0%  (0) | 0%  (0) | 54.5%  (6) | NA | NA | 54%  (6) | 0%  (0) | NA | 9.1%  (1) | NA | NA | 90.9%  (10) | NA | 90.9%  (10) | 9.1%  (1) | NA | NA | NA |
| Ecohealth | 2.153 | 58 | United States | Springer New York | 2004-2020 | 90 | 0%  (0) | 30%  (3) | 38.1%  (24) | 16%  (4) | NA | 32%  (28) | 2%  (2) | 1.1%  (1) | 4.4%  (4) | NA | 5.6%  (5) | 88.9%  (80) | NA | 88.9%  (80) | 7.8%  (7) | 2.2%  (2) | NA | 1.1%  (1) |
| Ecohydrology | 2.767 | 54 | United Kingdom | John Wiley and Sons Ltd | 2009-2020 | 37 | 0%  (0) | 0%  (0) | 19.4%  (7) | NA | NA | 20%  (7) | 3%  (1) | NA | 10.8%  (4) | NA | 2.7%  (1) | 86.5%  (32) | NA | 89.2%  (33) | 10.8%  (4) | NA | NA | NA |
| Ecological Applications | 4.248 | 213 | United States | Wiley-Blackwell | 1991-2020 | 37 | 0%  (0) | 0%  (0) | 32.4%  (12) | NA | NA | 32%  (12) | 0%  (0) | NA | NA | 2.7%  (1) | 8.1%  (3) | 89.2%  (33) | NA | 91.9%  (34) | 8.1%  (3) | NA | NA | NA |
| Ecological Chemistry and Engineering S-Chemia I Inzynieria Ekologiczna S | 1.488 | 21 | Germany | De Gruyter Open Ltd. | 2008-2020 | 21 | 100%  (1) | 100%  (1) | 42.9%  (6) | 0%  (0) | NA | 28%  (6) | 0%  (0) | NA | 4.8%  (1) | 66.7%  (14) | NA | 28.6%  (6) | NA | 85.7%  (18) | 9.5%  (2) | 4.8%  (1) | NA | NA |
| Ecological Economics | 4.482 | 202 | Netherlands | Elsevier | 1989-2021 | 63 | 0%  (0) | 28.6%  (4) | 17.7%  (11) | NA | NA | 18%  (11) | 2%  (1) | 6.3%  (4) | 9.5%  (6) | NA | 4.8%  (3) | 79.4%  (50) | NA | 82.5%  (52) | 9.5%  (6) | 7.9%  (5) | NA | NA |
| Ecological Engineering | 3.512 | 128 | Netherlands | Elsevier | 1992-2020 | 20 | 0%  (0) | 20%  (1) | 26.3%  (5) | NA | NA | 26%  (5) | 5%  (1) | NA | 15%  (3) | 15%  (3) | 5%  (1) | 65%  (13) | NA | 85%  (17) | 15%  (3) | NA | NA | NA |
| Ecological Indicators | 4.229 | 127 | Netherlands | Elsevier | 2001-2021 | 80 | 0%  (0) | 50%  (4) | 14.6%  (7) | 11.5%  (3) | NA | 14%  (10) | 8%  (6) | 1.2%  (1) | 27.5%  (22) | 3.8%  (3) | 2.5%  (2) | 65%  (52) | NA | 70%  (56) | 15%  (12) | 15%  (12) | NA | NA |
| Ecological Processes | 1.642 | 22 | Switzerland | Springer International Publishing AG | 2012-2020 | 79 | 0%  (0) | 0%  (0) | 30.6%  (22) | 0%  (0) | NA | 28%  (22) | 0%  (0) | NA | 32.9%  (26) | 3.8%  (3) | 6.3%  (5) | 57%  (45) | NA | 58.2%  (46) | 34.2%  (27) | 7.6%  (6) | NA | NA |
| Ecology and Society | 3.89 | 141 | Canada | The Resilience Alliance | 1997-2020 | 106 | 0%  (0) | 0%  (0) | 40.6%  (43) | NA | NA | 40%  (43) | 0%  (0) | 2.8%  (3) | 2.8%  (3) | NA | 2.8%  (3) | 91.5%  (97) | NA | 94.3%  (100) | 5.7%  (6) | NA | NA | NA |
| Economics of Energy & Environmental Policy | 3.217 | 23 | United States | International Association for Energy Economics | 2012-2020 | 36 | 0%  (0) | 33.3%  (1) | 33.3%  (12) | NA | NA | 34%  (12) | 0%  (0) | 2.8%  (1) | 8.3%  (3) | NA | 11.1%  (4) | 77.8%  (28) | NA | 86.1%  (31) | 11.1%  (4) | 2.8%  (1) | NA | NA |
| Ecosystem Health and Sustainability | 2.315 | 21 | United Kingdom | Taylor and Francis Ltd. | 2015-2020 | 105 | 0%  (0) | 0%  (0) | 16.9%  (14) | 13.6%  (3) | NA | 16%  (17) | 0%  (0) | 1.9%  (2) | 32.4%  (34) | 4.8%  (5) | 6.7%  (7) | 54.3%  (57) | NA | 61.9%  (65) | 33.3%  (35) | 4.8%  (5) | NA | NA |
| Ecosystem Services | 6.33 | 69 | Netherlands | Elsevier BV | 2012-2020 | 52 | 0%  (0) | 37.5%  (6) | 25.5%  (13) | NA | NA | 26%  (13) | 2%  (1) | 1.9%  (1) | 11.5%  (6) | 1.9%  (1) | 5.8%  (3) | 78.8%  (41) | NA | 88.5%  (46) | 9.6%  (5) | 1.9%  (1) | NA | NA |
| Ecotoxicology | 2.535 | 90 | Netherlands | Springer Netherlands | 1992-2020 | 61 | 0%  (0) | 26.8%  (15) | 24.6%  (15) | NA | NA | 24%  (15) | 0%  (0) | NA | 13.1%  (8) | 3.3%  (2) | 6.6%  (4) | 77%  (47) | NA | 82%  (50) | 14.8%  (9) | 3.3%  (2) | NA | NA |
| Ecotoxicology and Environmental Safety | 4.872 | 129 | United States | Academic Press Inc. | 1977-2021 | 50 | 0%  (0) | 18.2%  (2) | 18.8%  (9) | 0%  (0) | NA | 18%  (9) | 0%  (0) | 4%  (2) | 60%  (30) | 4%  (2) | 8%  (4) | 24%  (12) | NA | 50%  (25) | 38%  (19) | 12%  (6) | NA | NA |
| Elementa-Science of The Anthropocene | 4.212 | 34 | United States | University of California Press | 2013-2020 | 14 | 0%  (0) | 0%  (0) | 64.3%  (9) | NA | NA | 64%  (9) | 0%  (0) | NA | NA | NA | NA | 100%  (14) | NA | 100%  (14) | NA | NA | NA | NA |
| Energy & Environment | 1.775 | 29 | United States | SAGE Publications Inc. | 1981, 1995-2020 | 57 | 0%  (0) | 11.8%  (2) | 14.3%  (7) | 0%  (0) | NA | 12%  (7) | 0%  (0) | 3.5%  (2) | 52.6%  (30) | 1.8%  (1) | NA | 42.1%  (24) | NA | 57.9%  (33) | 28.1%  (16) | 14%  (8) | NA | NA |
| Energy & Environmental Science | 30.289 | 343 | United Kingdom | Royal Society of Chemistry | 2008-2020 | 53 | 0%  (0) | 0%  (0) | 50%  (4) | 8.9%  (4) | NA | 16%  (8) | 0%  (0) | NA | 26.4%  (14) | 1.9%  (1) | 1.9%  (1) | 69.8%  (37) | NA | 86.8%  (46) | 9.4%  (5) | 3.8%  (2) | NA | NA |
| Energy Efficiency | 1.81 | 41 | Netherlands | Springer Netherlands | 2008-2020 | 54 | 0%  (0) | 44.4%  (4) | 24.1%  (13) | NA | NA | 24%  (13) | 0%  (0) | NA | 11.1%  (6) | 1.9%  (1) | 1.9%  (1) | 85.2%  (46) | NA | 90.7%  (49) | 7.4%  (4) | 1.9%  (1) | NA | NA |
| Energy Journal | 2.394 | 77 | United States | International Association for Energy Economics | 1994-2021 | 37 | 0%  (0) | 20%  (1) | 21.6%  (8) | NA | NA | 22%  (8) | 0%  (0) | 2.7%  (1) | 18.9%  (7) | NA | 5.4%  (2) | 73%  (27) | NA | 89.2%  (33) | 8.1%  (3) | 2.7%  (1) | NA | NA |
| Energy Policy | 5.042 | 217 | United Kingdom | Elsevier BV | 1973-2020 | 46 | 0%  (0) | 33.3%  (2) | 37.5%  (3) | 23.7%  (9) | NA | 26%  (12) | 0%  (0) | NA | 23.9%  (11) | 2.2%  (1) | 2.2%  (1) | 71.7%  (33) | NA | 76.1%  (35) | 23.9%  (11) | NA | NA | NA |
| Energy Research & Social Science | 4.771 | 63 | United Kingdom | Elsevier Ltd. | 2014-2020 | 61 | 0%  (0) | 16.7%  (1) | 39.3%  (24) | NA | NA | 40%  (24) | 0%  (0) | NA | 4.9%  (3) | 1.6%  (1) | NA | 93.4%  (57) | NA | 96.7%  (59) | 1.6%  (1) | 1.6%  (1) | NA | NA |
| Enviromental Reviews | 4.176 | 68 | Canada | National Research Council of Canada | 1993-2020 | 22 | 0%  (0) | 0%  (0) | 0%  (0) | 38.1%  (8) | NA | 36%  (8) | 0%  (0) | NA | NA | NA | NA | 100%  (22) | NA | 100%  (22) | NA | NA | NA | NA |
| Environment and Behavior | 5.141 | 114 | United Kingdom | SAGE Publications Ltd | 1969-2020 | 81 | 0%  (0) | 33.3%  (5) | 33.8%  (27) | NA | NA | 34%  (27) | 1%  (1) | NA | NA | NA | 1.2%  (1) | 98.8%  (80) | NA | 98.8%  (80) | 1.2%  (1) | NA | NA | NA |
| Environment and Development Economics | 1.429 | 62 | United Kingdom | Cambridge University Press | 1996-2020 | 41 | 25%  (1) | 36.4%  (8) | 34.1%  (14) | NA | NA | 34%  (14) | 0%  (0) | 7.3%  (3) | 12.2%  (5) | NA | 12.2%  (5) | 68.3%  (28) | NA | 70.7%  (29) | 22%  (9) | 2.4%  (1) | 4.9%  (2) | NA |
| Environment and History | 0.698 | 25 | United Kingdom | White Horse Press | 1995-2020 | 29 | 100%  (1) | 100%  (1) | 44%  (11) | 50%  (2) | NA | 44%  (13) | 0%  (0) | 3.4%  (1) | 6.9%  (2) | 10.3%  (3) | 6.9%  (2) | 72.4%  (21) | NA | 86.2%  (25) | 13.8%  (4) | NA | NA | NA |
| Environment and Planning A-Economy and Space | 3.033 | 129 | United Kingdom | SAGE Publications Ltd | 1973-2020 | 38 | NA | NA | 50%  (4) | 40%  (12) | NA | 42%  (16) | 0%  (0) | NA | 7.9%  (3) | NA | NA | 92.1%  (35) | NA | 100%  (38) | NA | NA | NA | NA |
| Environment and Planning B-Urban Analytics and City Science | 2.822 | 90 | United Kingdom | SAGE Publications Ltd | 2017-2020 | 25 | NA | NA | 20%  (1) | 60%  (12) | NA | 52%  (13) | 0%  (0) | NA | 8%  (2) | NA | NA | 92%  (23) | NA | 96%  (24) | 4%  (1) | NA | NA | NA |
| Environment and Planning C-Politics and Space | 2.601 | 69 | United Kingdom | SAGE Publications Ltd | 2017-2020 | 38 | 60%  (3) | 60%  (3) | 52.6%  (20) | NA | NA | 52%  (20) | 0%  (0) | 7.9%  (3) | 7.9%  (3) | NA | 7.9%  (3) | 76.3%  (29) | NA | 81.6%  (31) | 15.8%  (6) | 2.6%  (1) | NA | NA |
| Environment and Planning D-Society & Space | 3.681 | 105 | United Kingdom | SAGE Publications Inc. | 1983-2020 | 32 | 33.3%  (1) | 33.3%  (1) | 33.3%  (1) | 72.4%  (21) | NA | 68%  (22) | 0%  (0) | 3.1%  (1) | 9.4%  (3) | NA | 3.1%  (1) | 84.4%  (27) | NA | 90.6%  (29) | 6.2%  (2) | 3.1%  (1) | NA | NA |
| Environment and Urbanization | 3.273 | 73 | United Kingdom | SAGE Publications Ltd | 1989-2020 | 29 | 66.7%  (2) | 80%  (4) | 55.6%  (5) | 45%  (9) | NA | 48%  (14) | 0%  (0) | 17.2%  (5) | 13.8%  (4) | NA | 10.3%  (3) | 58.6%  (17) | NA | 62.1%  (18) | 13.8%  (4) | 20.7%  (6) | 3.4%  (1) | NA |
| Environment Development and Sustainability | 2.191 | 56 | Netherlands | Springer Netherlands | 1999-2020 | 58 | 0%  (0) | 29.4%  (10) | 22.4%  (13) | NA | NA | 22%  (13) | 0%  (0) | 5.2%  (3) | 32.8%  (19) | 1.7%  (1) | 3.4%  (2) | 56.9%  (33) | NA | 60.3%  (35) | 19%  (11) | 20.7%  (12) | NA | NA |
| Environment International | 7.577 | 191 | United Kingdom | Elsevier Ltd. | 1976-2020 | 83 | 25%  (1) | 35.3%  (6) | 33.7%  (28) | NA | NA | 34%  (28) | 0%  (0) | NA | 25.3%  (21) | 1.2%  (1) | 2.4%  (2) | 71.1%  (59) | NA | 79.5%  (66) | 20.5%  (17) | NA | NA | NA |
| Environmental & Resource Economics | 2.286 | 92 | Netherlands | Springer Netherlands | 1991-2020 | 99 | 0%  (0) | 0%  (0) | 32.4%  (11) | 10.8%  (7) | NA | 18%  (18) | 0%  (0) | NA | 4%  (4) | 1%  (1) | NA | 94.9%  (94) | NA | 97%  (96) | 2%  (2) | 1%  (1) | NA | NA |
| Environmental and Ecological Statistics | 0.981 | 46 | Netherlands | Springer Netherlands | 1994-2020 | 25 | 0%  (0) | 0%  (0) | 16%  (4) | NA | NA | 16%  (4) | 0%  (0) | NA | 8%  (2) | NA | 12%  (3) | 80%  (20) | NA | 88%  (22) | 12%  (3) | NA | NA | NA |
| Environmental and Experimental Botany | 4.027 | 131 | Netherlands | Elsevier | 1976-2020 | 62 | 0%  (0) | 0%  (0) | 16.7%  (1) | 10.7%  (6) | NA | 12%  (7) | 0%  (0) | 1.6%  (1) | 19.4%  (12) | 6.5%  (4) | 6.5%  (4) | 66.1%  (41) | NA | 72.6%  (45) | 22.6%  (14) | 4.8%  (3) | NA | NA |
| Environmental and Molecular Mutagenesis | 3.131 | 87 | United States | Wiley-Liss Inc. | 1987-2020 | 42 | 0%  (0) | 33.3%  (1) | 35.7%  (15) | NA | NA | 36%  (15) | 0%  (0) | NA | 2.4%  (1) | NA | 4.8%  (2) | 92.9%  (39) | NA | 95.2%  (40) | 4.8%  (2) | NA | NA | NA |
| Environmental Chemistry | 1.91 | 62 | Australia | CSIRO | 2004-2020 | 28 | 0%  (0) | 0%  (0) | 30%  (3) | 5.6%  (1) | NA | 14%  (4) | 0%  (0) | NA | 10.7%  (3) | NA | NA | 89.3%  (25) | NA | 92.9%  (26) | 7.1%  (2) | NA | NA | NA |
| Environmental Chemistry Letters | 5.922 | 64 | Germany | Springer Verlag | 2003-2020 | 46 | 0%  (0) | 0%  (0) | 23.9%  (11) | NA | NA | 24%  (11) | 0%  (0) | 4.3%  (2) | 43.5%  (20) | 6.5%  (3) | NA | 45.7%  (21) | NA | 54.3%  (25) | 30.4%  (14) | 15.2%  (7) | NA | NA |
| Environmental Communication-A Journal of Nature and Culture | 1.787 | 30 | United Kingdom | Taylor and Francis Ltd. | 2010-2020 | 70 | 100%  (1) | 50%  (4) | 52.9%  (37) | NA | NA | 52%  (37) | 0%  (0) | NA | 7.1%  (5) | NA | NA | 92.9%  (65) | NA | 95.7%  (67) | 4.3%  (3) | NA | NA | NA |
| Environmental Conservation | 2.434 | 87 | United Kingdom | Cambridge University Press | 1974-2020 | 15 | 0%  (0) | 0%  (0) | 14.3%  (1) | 0%  (0) | NA | 6%  (1) | 0%  (0) | 13.3%  (2) | NA | NA | NA | 86.7%  (13) | NA | 86.7%  (13) | 6.7%  (1) | 6.7%  (1) | NA | NA |
| Environmental Development | 2.4 | 31 | Netherlands | Elsevier BV | 2012-2020 | 35 | 0%  (0) | 22.2%  (2) | 22.9%  (8) | NA | NA | 22%  (8) | 0%  (0) | 20%  (7) | 14.3%  (5) | 2.9%  (1) | 8.6%  (3) | 54.3%  (19) | NA | 60%  (21) | 20%  (7) | 17.1%  (6) | 2.9%  (1) | NA |
| Environmental Earth Sciences | 2.18 | 118 | Germany | Springer Verlag | 2009-2020 | 54 | 0%  (0) | 8.3%  (1) | 9.3%  (5) | NA | NA | 10%  (5) | 0%  (0) | NA | 11.1%  (6) | 7.4%  (4) | 1.9%  (1) | 79.6%  (43) | NA | 85.2%  (46) | 14.8%  (8) | NA | NA | NA |
| Environmental Education Research | 2.266 | 71 | United Kingdom | Carfax Publishing Ltd. | 1995-2020 | 35 | 0%  (0) | 50%  (2) | 50%  (2) | 58.1%  (18) | NA | 58%  (20) | 0%  (0) | 2.9%  (1) | 5.7%  (2) | NA | 2.9%  (1) | 88.6%  (31) | NA | 94.3%  (33) | 5.7%  (2) | NA | NA | NA |
| Environmental Engineering Research | 1.438 | 21 | South Korea | Korean Society of Environmental Engineers | 2011-2020 | 35 | 0%  (0) | 15.8%  (3) | 15.8%  (3) | 0%  (0) | NA | 8%  (3) | 0%  (0) | NA | 57.1%  (20) | NA | NA | 42.9%  (15) | NA | 88.6%  (31) | 8.6%  (3) | 2.9%  (1) | NA | NA |
| Environmental Engineering Science | 1.681 | 65 | United States | Mary Ann Liebert Inc. | 1997-2020 | 27 | 100%  (1) | 33.3%  (2) | 25.9%  (7) | NA | NA | 26%  (7) | 0%  (0) | NA | 22.2%  (6) | 3.7%  (1) | 3.7%  (1) | 70.4%  (19) | NA | 92.6%  (25) | 3.7%  (1) | 3.7%  (1) | NA | NA |
| Environmental Ethics | 0.275 | 29 | United States | Environmental Philosophy Inc | 1979-1981, 1984, 1988, 1990, 1996-2019 | 42 | 0%  (0) | 0%  (0) | 0%  (0) | 25%  (10) | NA | 24%  (10) | 0%  (0) | 2.4%  (1) | 7.1%  (3) | NA | 2.4%  (1) | 88.1%  (37) | NA | 92.9%  (39) | 4.8%  (2) | NA | 2.4%  (1) | NA |
| Environmental Evidence | 3.708 | 28 | United Kingdom | BioMed Central Ltd. | 2011-2020 | 26 | 0%  (0) | 0%  (0) | 50%  (13) | NA | NA | 50%  (13) | 0%  (0) | 3.8%  (1) | NA | 3.8%  (1) | NA | 92.3%  (24) | NA | 96.2%  (25) | 3.8%  (1) | NA | NA | NA |
| Environmental Fluid Mechanics | 1.512 | 44 | Netherlands | Springer Netherlands | 2001-2020 | 40 | 0%  (0) | 27.3%  (3) | 15%  (6) | NA | NA | 16%  (6) | 0%  (0) | NA | 17.5%  (7) | NA | NA | 82.5%  (33) | NA | 90%  (36) | 2.5%  (1) | 7.5%  (3) | NA | NA |
| Environmental Forensics | 0.726 | 37 | United Kingdom | Taylor and Francis Ltd. | 2000-2020 | 71 | 100%  (1) | 12.5%  (1) | 12.7%  (9) | NA | NA | 12%  (9) | 0%  (0) | NA | 4.2%  (3) | NA | 1.4%  (1) | 94.4%  (67) | NA | 95.8%  (68) | 4.2%  (3) | NA | NA | NA |
| Environmental Hazards-Human and Policy Dimensions | 1.133 | 46 | United Kingdom | Taylor and Francis Ltd. | 1983, 1999-2003, 2005, 2007, 2009-2020 | 45 | 0%  (0) | 0%  (0) | 40%  (18) | NA | NA | 40%  (18) | 0%  (0) | 8.9%  (4) | 6.7%  (3) | 4.4%  (2) | 11.1%  (5) | 68.9%  (31) | NA | 71.1%  (32) | 17.8%  (8) | 8.9%  (4) | 2.2%  (1) | NA |
| Environmental History | 0.593 | 36 | United Kingdom | Oxford University Press | 1996-2020 | 23 | 0%  (0) | 0%  (0) | 56.5%  (13) | NA | NA | 56%  (13) | 0%  (0) | NA | 8.7%  (2) | NA | 4.3%  (1) | 87%  (20) | NA | 91.3%  (21) | 8.7%  (2) | NA | NA | NA |
| Environmental Impact Assessment Review | 4.135 | 92 | United States | Elsevier Inc. | 1980-1983, 1985-2020 | 31 | 0%  (0) | 0%  (0) | 0%  (0) | 25.9%  (7) | NA | 22%  (7) | 0%  (0) | 3.2%  (1) | 12.9%  (4) | NA | 3.2%  (1) | 80.6%  (25) | NA | 83.9%  (26) | 16.1%  (5) | NA | NA | NA |
| Environmental Innovation and Societal Transitions | 8.4 | 52 | Netherlands | Elsevier BV | 2011-2020 | 40 | 0%  (0) | 16.7%  (1) | 12.5%  (5) | NA | NA | 12%  (5) | 0%  (0) | NA | 7.5%  (3) | NA | NA | 92.5%  (37) | NA | 95%  (38) | 2.5%  (1) | 2.5%  (1) | NA | NA |
| Environmental Management | 2.561 | 118 | United States | Springer New York | 1976-2020 | 56 | 0%  (0) | 28.6%  (4) | 23.2%  (13) | NA | NA | 24%  (13) | 0%  (0) | 1.8%  (1) | 19.6%  (11) | 1.8%  (1) | 1.8%  (1) | 75%  (42) | NA | 82.1%  (46) | 8.9%  (5) | 8.9%  (5) | NA | NA |
| Environmental Microbiology Reports | 2.975 | 69 | United States | Wiley-Blackwell | 2009-2020 | 227 | 27.3%  (3) | 27.3%  (3) | 25.1%  (57) | NA | NA | 26%  (57) | 0%  (0) | 0.4%  (1) | 4.4%  (10) | 0.4%  (1) | 0.4%  (1) | 94.3%  (214) | NA | 96.5%  (219) | 3.1%  (7) | 0.4%  (1) | NA | NA |
| Environmental Modeling & Assessment | 1.634 | 49 | Netherlands | Springer Netherlands | 1997-2020 | 30 | 0%  (0) | 0%  (0) | 20%  (6) | NA | NA | 20%  (6) | 0%  (0) | 3.3%  (1) | 6.7%  (2) | 6.7%  (2) | 3.3%  (1) | 80%  (24) | NA | 93.3%  (28) | 6.7%  (2) | NA | NA | NA |
| Environmental Modelling & Software | 4.807 | 136 | Netherlands | Elsevier BV | 1997-2020 | 56 | 0%  (0) | 29.4%  (5) | 23.2%  (13) | NA | NA | 24%  (13) | 0%  (0) | NA | 10.7%  (6) | NA | NA | 89.3%  (50) | NA | 91.1%  (51) | 8.9%  (5) | NA | NA | NA |
| Environmental Monitoring and Assessment | 1.903 | 109 | Netherlands | Springer Netherlands | 1981-2020 | 32 | 0%  (0) | 28.6%  (2) | 18.8%  (6) | NA | NA | 18%  (6) | 0%  (0) | 3.1%  (1) | 25%  (8) | 6.2%  (2) | 9.4%  (3) | 56.2%  (18) | NA | 59.4%  (19) | 21.9%  (7) | 18.8%  (6) | NA | NA |
| Environmental Policy and Governance | 2.618 | 48 | United Kingdom | John Wiley and Sons Ltd | 2009-2020 | 42 | 0%  (0) | 33.3%  (1) | 21.4%  (9) | NA | NA | 22%  (9) | 0%  (0) | 7.1%  (3) | 11.9%  (5) | 7.1%  (3) | 7.1%  (3) | 66.7%  (28) | NA | 78.6%  (33) | 11.9%  (5) | 9.5%  (4) | NA | NA |
| Environmental Politics | 4.32 | 71 | United Kingdom | Routledge | 1992-2020 | 52 | 0%  (0) | 0%  (0) | 28.6%  (2) | 40%  (18) | NA | 38%  (20) | 0%  (0) | NA | 1.9%  (1) | NA | 3.8%  (2) | 94.2%  (49) | NA | 96.2%  (50) | 1.9%  (1) | 1.9%  (1) | NA | NA |
| Environmental Pollutants and Bioavailability | Not Available | 30 | United Kingdom | Taylor and Francis Ltd. | 2019-2020 | 14 | 0%  (0) | 0%  (0) | 7.1%  (1) | NA | NA | 8%  (1) | 0%  (0) | NA | 57.1%  (8) | NA | NA | 42.9%  (6) | NA | 50%  (7) | 50%  (7) | NA | NA | NA |
| Environmental Pollution | 6.793 | 227 | United Kingdom | Elsevier Ltd. | 1970-1980, 1986-2020 | 70 | 0%  (0) | 16.7%  (3) | 18.6%  (13) | NA | NA | 18%  (13) | 0%  (0) | 1.4%  (1) | 28.6%  (20) | NA | 7.1%  (5) | 62.9%  (44) | NA | 65.7%  (46) | 31.4%  (22) | 1.4%  (1) | 1.4%  (1) | NA |
| Environmental Progress & Sustainable Energy | 1.989 | 66 | United States | John Wiley and Sons Inc. | 2009-2020 | 20 | 0%  (0) | 0%  (0) | 33.3%  (1) | 23.5%  (4) | NA | 24%  (5) | 0%  (0) | NA | NA | NA | NA | 100%  (20) | NA | 100%  (20) | NA | NA | NA | NA |
| Environmental Research Letters | 6.096 | 124 | United Kingdom | IOP Publishing Ltd. | 2006-2020 | 31 | 0%  (0) | 60%  (12) | 48.4%  (15) | NA | NA | 48%  (15) | 0%  (0) | 3.2%  (1) | 12.9%  (4) | NA | NA | 83.9%  (26) | NA | 87.1%  (27) | 9.7%  (3) | 3.2%  (1) | NA | NA |
| Environmental Science & Policy | 4.767 | 115 | Netherlands | Elsevier BV | 1998-2020 | 58 | 100%  (2) | 53.8%  (7) | 17.2%  (10) | NA | NA | 18%  (10) | 0%  (0) | NA | 12.1%  (7) | NA | 1.7%  (1) | 86.2%  (50) | NA | 89.7%  (52) | 6.9%  (4) | 3.4%  (2) | NA | NA |
| Environmental Science & Technology | 7.864 | 397 | United States | American Chemical Society | 1958, 1967-2020 | 103 | 100%  (1) | 40%  (2) | 35.7%  (10) | 30.7%  (23) | NA | 32%  (33) | 0%  (0) | NA | 22.3%  (23) | NA | 3.9%  (4) | 73.8%  (76) | NA | 77.7%  (80) | 19.4%  (20) | 2.9%  (3) | NA | NA |
| Environmental Science & Technology Letters | 7.678 | 58 | United States | American Chemical Society | 2013-2020 | 58 | 0%  (0) | 0%  (0) | 28.6%  (2) | 37.3%  (19) | NA | 36%  (21) | 0%  (0) | NA | 22.4%  (13) | NA | 1.7%  (1) | 75.9%  (44) | NA | 82.8%  (48) | 15.5%  (9) | 1.7%  (1) | NA | NA |
| Environmental Science and Pollution Research | 3.056 | 113 | Germany | Springer Science + Business Media | 1994-2020 | 105 | 0%  (0) | 18.9%  (10) | 21%  (22) | NA | NA | 20%  (22) | 0%  (0) | 1%  (1) | 25.7%  (27) | 5.7%  (6) | 2.9%  (3) | 64.8%  (68) | NA | 76.2%  (80) | 21%  (22) | 2.9%  (3) | NA | NA |
| Environmental Science-Nano | 7.683 | 67 | United Kingdom | Royal Society of Chemistry | 2014-2020 | 39 | 0%  (0) | 42.9%  (3) | 50%  (5) | 37.9%  (11) | NA | 42%  (16) | 0%  (0) | NA | 17.9%  (7) | 2.6%  (1) | NA | 79.5%  (31) | NA | 84.6%  (33) | 15.4%  (6) | NA | NA | NA |
| Environmental Science-Processes & Impacts | 3.238 | 98 | United Kingdom | Royal Society of Chemistry | 2012-2020 | 40 | 0%  (0) | 40%  (2) | 47.5%  (19) | NA | NA | 48%  (19) | 0%  (0) | NA | 20%  (8) | NA | NA | 80%  (32) | NA | 82.5%  (33) | 15%  (6) | 2.5%  (1) | NA | NA |
| Environmental Science-Water Research & Technology | 3.449 | 37 | United Kingdom | Royal Society of Chemistry | 2015-2020 | 43 | 100%  (1) | 42.9%  (3) | 41.9%  (18) | NA | NA | 42%  (18) | 0%  (0) | NA | 32.6%  (14) | NA | NA | 67.4%  (29) | NA | 74.4%  (32) | 20.9%  (9) | 4.7%  (2) | NA | NA |
| Environmental Sciences Europe | 5.394 | 35 | Germany | Springer Verlag | 2011-2020 | 75 | 0%  (0) | 0%  (0) | 22.6%  (7) | 38.6%  (17) | NA | 32%  (24) | 0%  (0) | NA | 10.7%  (8) | 5.3%  (4) | 2.7%  (2) | 81.3%  (61) | NA | 85.3%  (64) | 13.3%  (10) | 1.3%  (1) | NA | NA |
| Environmental Technology | 2.213 | 73 | United Kingdom | Taylor and Francis Ltd. | 1990-2020 | 48 | NA | 28.6%  (2) | 17.8%  (8) | NA | NA | 18%  (8) | 6%  (3) | NA | 45.8%  (22) | 2.1%  (1) | 8.3%  (4) | 43.8%  (21) | NA | 60.4%  (29) | 31.2%  (15) | 8.3%  (4) | NA | NA |
| Environmental Technology & Innovation | 3.356 | 28 | Netherlands | Elsevier BV | 2014-2020 | 27 | 0%  (0) | 11.1%  (1) | 18.5%  (5) | NA | NA | 18%  (5) | 0%  (0) | 7.4%  (2) | 51.9%  (14) | NA | 3.7%  (1) | 37%  (10) | NA | 48.1%  (13) | 33.3%  (9) | 18.5%  (5) | NA | NA |
| Environmental Toxicology | 3.118 | 77 | United States | John Wiley and Sons Inc. | 1980, 1999-2020 | 50 | 0%  (0) | 22.2%  (2) | 20.4%  (10) | NA | NA | 20%  (10) | 2%  (1) | 2%  (1) | 10%  (5) | NA | 16%  (8) | 72%  (36) | NA | 86%  (43) | 14%  (7) | NA | NA | NA |
| Environmental Toxicology and Chemistry | 3.152 | 171 | United States | Wiley-Blackwell | 1982-2020 | 126 | 0%  (0) | 16.7%  (9) | 34.1%  (43) | NA | NA | 34%  (43) | 0%  (0) | 3.2%  (4) | 7.9%  (10) | NA | 3.2%  (4) | 85.7%  (108) | NA | 86.5%  (109) | 11.9%  (15) | 0.8%  (1) | 0.8%  (1) | NA |
| Environmental Toxicology and Pharmacology | 3.292 | 82 | Netherlands | Elsevier | 1996-2020 | 45 | 0%  (0) | 40%  (2) | 24.4%  (11) | NA | NA | 24%  (11) | 0%  (0) | NA | 4.4%  (2) | 2.2%  (1) | NA | 93.3%  (42) | NA | 93.3%  (42) | 6.7%  (3) | NA | NA | NA |
| Environmental Values | 2.158 | 47 | United Kingdom | White Horse Press | 1994-2020 | 28 | 0%  (0) | 20%  (1) | 42.9%  (12) | NA | NA | 42%  (12) | 0%  (0) | NA | NA | 3.6%  (1) | NA | 96.4%  (27) | NA | 96.4%  (27) | 3.6%  (1) | NA | NA | NA |
| Environmetrics | 1.039 | 58 | United Kingdom | John Wiley and Sons Ltd | 1990-2020 | 56 | 0%  (0) | 0%  (0) | 32.1%  (18) | NA | NA | 32%  (18) | 0%  (0) | NA | 7.1%  (4) | NA | 8.9%  (5) | 83.9%  (47) | NA | 89.3%  (50) | 8.9%  (5) | NA | NA | 1.8%  (1) |
| Estuaries and Coasts | 2.319 | 105 | United States | Springer New York | 1978-2001, 2006-2020 | 51 | 0%  (0) | 0%  (0) | 13.7%  (7) | NA | NA | 14%  (7) | 0%  (0) | NA | 11.8%  (6) | NA | 2%  (1) | 86.3%  (44) | NA | 90.2%  (46) | 9.8%  (5) | NA | NA | NA |
| European Planning Studies | 2.226 | 81 | United Kingdom | Routledge | 1993-2020 | 25 | 0%  (0) | 0%  (0) | 20%  (5) | NA | NA | 20%  (5) | 0%  (0) | NA | NA | 4%  (1) | NA | 96%  (24) | NA | 100%  (25) | NA | NA | NA | NA |
| European Urban and Regional Studies | 2.855 | 62 | United Kingdom | SAGE Publications Ltd | 1994-2020 | 33 | 0%  (0) | 40%  (2) | 27.3%  (9) | NA | NA | 28%  (9) | 0%  (0) | NA | 6.1%  (2) | 9.1%  (3) | NA | 84.8%  (28) | NA | 100%  (33) | NA | NA | NA | NA |
| Extractive Industries and Society-An International Journal | 2.528 | 29 | Netherlands | Elsevier BV | 2014-2020 | 30 | 0%  (0) | 0%  (0) | 30%  (9) | NA | NA | 30%  (9) | 0%  (0) | 3.3%  (1) | 3.3%  (1) | NA | 3.3%  (1) | 90%  (27) | NA | 93.3%  (28) | 3.3%  (1) | 3.3%  (1) | NA | NA |
| Food and Environmental Virology | 2.819 | 32 | United States | Springer New York | 2009-2020 | 33 | 0%  (0) | 28.6%  (2) | 30.3%  (10) | NA | NA | 30%  (10) | 0%  (0) | 6.1%  (2) | 15.2%  (5) | 6.1%  (2) | 3%  (1) | 69.7%  (23) | NA | 87.9%  (29) | 6.1%  (2) | 6.1%  (2) | NA | NA |
| Forest Policy and Economics | 3.139 | 68 | Netherlands | Elsevier | 2000-2020 | 68 | 0%  (0) | 0%  (0) | 36.4%  (4) | 24.6%  (14) | NA | 26%  (18) | 0%  (0) | NA | 14.7%  (10) | NA | 2.9%  (2) | 82.4%  (56) | NA | 88.2%  (60) | 8.8%  (6) | 2.9%  (2) | NA | NA |
| Fresenius Environmental Bulletin | 0.553 | 38 | Germany | Parlar Scientific Publications | 1993-2020 | 15 | 0%  (0) | 7.1%  (1) | 7.1%  (1) | NA | NA | 8%  (1) | 7%  (1) | NA | NA | 6.7%  (1) | NA | 93.3%  (14) | NA | 80%  (12) | 20%  (3) | NA | NA | NA |
| Frontiers In Ecology and The Environment | 9.295 | 164 | United States | Wiley-Blackwell | 2003-2020 | 70 | NA | NA | 32.9%  (23) | NA | NA | 32%  (23) | 0%  (0) | 2.9%  (2) | 5.7%  (4) | NA | 4.3%  (3) | 87.1%  (61) | NA | 88.6%  (62) | 8.6%  (6) | 2.9%  (2) | NA | NA |
| Frontiers In Environmental Science | 2.749 | 37 | Switzerland | Frontiers Media S.A. | 2013-2020 | 16 | 0%  (0) | 0%  (0) | 50%  (8) | NA | NA | 50%  (8) | 0%  (0) | NA | 12.5%  (2) | 6.2%  (1) | NA | 81.2%  (13) | NA | 87.5%  (14) | 6.2%  (1) | 6.2%  (1) | NA | NA |
| Frontiers of Environmental Science & Engineering | 4.053 | 43 | United States | Springer Science + Business Media | 2012-2021 | 73 | 0%  (0) | 20%  (1) | 10.4%  (5) | 0%  (0) | NA | 6%  (5) | 0%  (0) | NA | 58.9%  (43) | NA | NA | 41.1%  (30) | NA | 49.3%  (36) | 50.7%  (37) | NA | NA | NA |
| GAIA-Ecological Perspectives For Science and Society | 1.87 | 28 | Germany | Oekom Verlag | 2005-2020 | 66 | 100%  (1) | 100%  (1) | 36.4%  (4) | 21.8%  (12) | NA | 24%  (16) | 0%  (0) | NA | NA | 1.5%  (1) | NA | 98.5%  (65) | NA | 100%  (66) | NA | NA | NA | NA |
| Geobiology | 4.385 | 72 | United Kingdom | Wiley-Blackwell Publishing Ltd | 2003-2020 | 77 | 0%  (0) | 0%  (0) | 25%  (3) | 40%  (26) | NA | 38%  (29) | 0%  (0) | 1.3%  (1) | 2.6%  (2) | NA | 1.3%  (1) | 94.8%  (73) | NA | 96.1%  (74) | 3.9%  (3) | NA | NA | NA |
| Geocarto International | 3.789 | 40 | United Kingdom | Taylor and Francis Ltd. | 1986-2020 | 5 | NA | NA | 0%  (0) | NA | NA | 0%  (0) | 0%  (0) | NA | 20%  (1) | 20%  (1) | NA | 60%  (3) | NA | 80%  (4) | NA | 20%  (1) | NA | NA |
| Geografisk Tidsskrift-Danish Journal of Geography | 1.577 | 25 | United Kingdom | Taylor and Francis Ltd. | 1972-2020 | 24 | 0%  (0) | 12.5%  (1) | 8.3%  (2) | NA | NA | 8%  (2) | 0%  (0) | NA | NA | NA | NA | 100%  (24) | NA | 100%  (24) | NA | NA | NA | NA |
| Geomicrobiology Journal | 1.989 | 77 | United Kingdom | Taylor and Francis Ltd. | 1978-1981, 1983-2020 | 27 | 0%  (0) | 0%  (0) | 14.8%  (4) | NA | NA | 14%  (4) | 0%  (0) | NA | 14.8%  (4) | NA | NA | 85.2%  (23) | NA | 88.9%  (24) | 7.4%  (2) | 3.7%  (1) | NA | NA |
| Global Biogeochemical Cycles | 4.608 | 47 | United Kingdom | Taylor and Francis Ltd. | 2009-2020 | 37 | 0%  (0) | 25%  (1) | 27%  (10) | NA | NA | 28%  (10) | 0%  (0) | NA | 5.4%  (2) | NA | 2.7%  (1) | 91.9%  (34) | NA | 91.9%  (34) | 8.1%  (3) | NA | NA | NA |
| Global Change Biology | 8.555 | 255 | United Kingdom | Wiley-Blackwell Publishing Ltd | 1995-2020 | 79 | 0%  (0) | 50%  (1) | 28%  (7) | 20.4%  (11) | NA | 22%  (18) | 0%  (0) | NA | 13.9%  (11) | NA | NA | 86.1%  (68) | NA | 89.9%  (71) | 10.1%  (8) | NA | NA | NA |
| Global Environmental Change-Human and Policy Dimensions | 10.466 | 177 | United Kingdom | Elsevier Ltd. | 1990-2020 | 52 | 0%  (0) | 33.3%  (2) | 44.2%  (23) | NA | NA | 44%  (23) | 0%  (0) | 5.8%  (3) | 9.6%  (5) | NA | 1.9%  (1) | 82.7%  (43) | NA | 84.6%  (44) | 9.6%  (5) | 5.8%  (3) | NA | NA |
| Global Environmental Politics | 3.091 | 56 | United States | MIT Press Journals | 2006-2020 | 55 | 33.3%  (1) | 57.1%  (4) | 40%  (22) | NA | NA | 40%  (22) | 0%  (0) | NA | 3.6%  (2) | NA | 3.6%  (2) | 92.7%  (51) | NA | 94.5%  (52) | 3.6%  (2) | 1.8%  (1) | NA | NA |
| Global Nest Journal | 0.983 | 43 | United Kingdom | Routledge | 2006-2020 | 16 | 0%  (0) | 0%  (0) | 18.8%  (3) | NA | NA | 18%  (3) | 0%  (0) | NA | 6.2%  (1) | NA | NA | 93.8%  (15) | NA | 93.8%  (15) | NA | 6.2%  (1) | NA | NA |
| Greenhouse Gases-Science and Technology | 1.979 | 32 | United Kingdom | John Wiley and Sons Ltd | 2011-2020 | 26 | 50%  (1) | 50%  (1) | 50%  (1) | 20.8%  (5) | NA | 24%  (6) | 0%  (0) | NA | 30.8%  (8) | NA | NA | 69.2%  (18) | NA | 73.1%  (19) | 19.2%  (5) | 7.7%  (2) | NA | NA |
| Grundwasser | 0.595 | 21 | Germany | Springer Verlag | 1997-2020 | 27 | 0%  (0) | 0%  (0) | 25.9%  (7) | NA | NA | 26%  (7) | 0%  (0) | NA | 3.7%  (1) | 7.4%  (2) | NA | 88.9%  (24) | NA | 96.3%  (26) | 3.7%  (1) | NA | NA | NA |
| Habitat International | 4.31 | 78 | United Kingdom | Elsevier Ltd. | 1970, 1976-2020 | 24 | 0%  (0) | 0%  (0) | 20.8%  (5) | NA | NA | 20%  (5) | 0%  (0) | 8.3%  (2) | 33.3%  (8) | NA | NA | 58.3%  (14) | NA | 70.8%  (17) | 25%  (6) | 4.2%  (1) | NA | NA |
| Housing Studies | 2.255 | 75 | United Kingdom | Routledge | 1986-2020 | 60 | NA | NA | 50%  (4) | 40.4%  (21) | NA | 42%  (25) | 0%  (0) | 1.7%  (1) | 11.7%  (7) | 1.7%  (1) | NA | 85%  (51) | NA | 93.3%  (56) | 6.7%  (4) | NA | NA | NA |
| Housing Theory & Society | 2.317 | 42 | United Kingdom | Taylor and Francis Ltd. | 1996-2020 | 30 | 0%  (0) | 25%  (1) | 28.6%  (2) | 43.5%  (10) | NA | 40%  (12) | 0%  (0) | NA | 6.7%  (2) | 3.3%  (1) | NA | 90%  (27) | NA | 93.3%  (28) | 6.7%  (2) | NA | NA | NA |
| Human and Ecological Risk Assessment | 2.3 | 67 | United Kingdom | Taylor and Francis Ltd. | 1995-2020 | 73 | 0%  (0) | 35.7%  (5) | 37%  (27) | NA | NA | 36%  (27) | 0%  (0) | 1.4%  (1) | 9.6%  (7) | 4.1%  (3) | NA | 84.9%  (62) | NA | 89%  (65) | 6.8%  (5) | 4.1%  (3) | NA | NA |
| Human Dimensions of Wildlife | 1.723 | 51 | United Kingdom | Taylor and Francis Ltd. | 1996-2020 | 32 | 0%  (0) | 0%  (0) | 25%  (8) | NA | NA | 24%  (8) | 0%  (0) | NA | 3.1%  (1) | NA | NA | 96.9%  (31) | NA | 100%  (32) | NA | NA | NA | NA |
| Human Ecology | 1.683 | 71 | United States | Springer Science + Business Media | 1972-2020 | 57 | 0%  (0) | 0%  (0) | 35.1%  (20) | NA | NA | 36%  (20) | 0%  (0) | NA | NA | NA | 3.5%  (2) | 96.5%  (55) | NA | 96.5%  (55) | 3.5%  (2) | NA | NA | NA |
| Human Ecology Review | 0.459 | 40 | United States | Society for Human Ecology | 1998-2019 | 37 | 50%  (1) | 50%  (1) | 27%  (10) | NA | NA | 28%  (10) | 0%  (0) | NA | NA | NA | 2.7%  (1) | 97.3%  (36) | NA | 97.3%  (36) | 2.7%  (1) | NA | NA | NA |
| Impact Assessment and Project Appraisal | 1.551 | 52 | United Kingdom | Taylor and Francis Ltd. | 1998-2020 | 28 | 0%  (0) | 0%  (0) | 46.4%  (13) | NA | NA | 46%  (13) | 0%  (0) | 3.6%  (1) | NA | NA | 7.1%  (2) | 89.3%  (25) | NA | 89.3%  (25) | 10.7%  (3) | NA | NA | NA |
| Integrated Environmental Assessment and Management | 3.44 | 57 | United States | Wiley-Blackwell | 2005-2020 | 67 | 0%  (0) | 47.4%  (9) | 51.5%  (34) | NA | NA | 52%  (34) | 1%  (1) | 3%  (2) | 7.5%  (5) | NA | 6%  (4) | 83.6%  (56) | NA | 82.1%  (55) | 14.9%  (10) | 3%  (2) | NA | NA |
| International Biodeterioration & Biodegradation | 4.074 | 103 | United Kingdom | Elsevier Ltd. | 1992-2020 | 51 | 0%  (0) | 14.3%  (1) | 28%  (14) | NA | NA | 28%  (14) | 2%  (1) | NA | 25.5%  (13) | 5.9%  (3) | 3.9%  (2) | 64.7%  (33) | NA | 70.6%  (36) | 27.5%  (14) | 2%  (1) | NA | NA |
| International Environmental Agreements-Politics Law and Economics | 2.511 | 37 | Netherlands | Springer Netherlands | 2003-2020 | 43 | 100%  (1) | 100%  (1) | 25%  (1) | 39.5%  (15) | NA | 38%  (16) | 2%  (1) | 2.3%  (1) | NA | NA | 2.3%  (1) | 95.3%  (41) | NA | 97.7%  (42) | NA | 2.3%  (1) | NA | NA |
| International Journal of Biometeorology | 2.68 | 92 | Germany | Springer New York | 1961-2020 | 79 | 0%  (0) | 0%  (0) | 23.8%  (5) | 36.2%  (21) | NA | 32%  (26) | 0%  (0) | 1.3%  (1) | 20.3%  (16) | 19%  (15) | 6.3%  (5) | 53.2%  (42) | NA | 78.5%  (62) | 19%  (15) | 2.5%  (2) | NA | NA |
| International Journal of Climate Change Strategies and Management | 1.791 | 21 | United Kingdom | Emerald Group Publishing Ltd. | 2009-2020 | 67 | 0%  (0) | 14.3%  (1) | 21.4%  (9) | 45.8%  (11) | NA | 30%  (20) | 1%  (1) | 25.4%  (17) | 17.9%  (12) | 9%  (6) | 9%  (6) | 38.8%  (26) | NA | 43.3%  (29) | 35.8%  (24) | 13.4%  (9) | 6%  (4) | 1.5%  (1) |
| International Journal of Environment and Pollution | 0.54 | 46 | United Kingdom | Inderscience Enterprises Ltd. | 1991-2014, 2020 | 41 | 0%  (0) | 0%  (0) | 7.7%  (3) | NA | NA | 8%  (3) | 5%  (2) | NA | 7.3%  (3) | NA | 7.3%  (3) | 85.4%  (35) | NA | 87.8%  (36) | 9.8%  (4) | 2.4%  (1) | NA | NA |
| International Journal of Environmental Analytical Chemistry | 1.431 | 42 | United Kingdom | Taylor and Francis Ltd. | 1971-2020 | 29 | 0%  (0) | 0%  (0) | 6.9%  (2) | NA | NA | 6%  (2) | 0%  (0) | NA | 17.2%  (5) | 6.9%  (2) | 3.4%  (1) | 72.4%  (21) | NA | 79.3%  (23) | 20.7%  (6) | NA | NA | NA |
| International Journal of Environmental Research | 2.007 | 38 | Switzerland | Springer International Publishing AG | 2007-2020 | 16 | 0%  (0) | 0%  (0) | 0%  (0) | NA | NA | 0%  (0) | 0%  (0) | NA | 81.2%  (13) | 6.2%  (1) | NA | 12.5%  (2) | NA | 18.8%  (3) | 6.2%  (1) | 75%  (12) | NA | NA |
| International Journal of Environmental Science and Technology | 2.54 | 75 | Iran | CEERS | 2005-2020 | 50 | 0%  (0) | 20.6%  (7) | 14.6%  (7) | NA | NA | 14%  (7) | 4%  (2) | 6%  (3) | 50%  (25) | 4%  (2) | 4%  (2) | 36%  (18) | NA | 50%  (25) | 22%  (11) | 26%  (13) | 2%  (1) | NA |
| International Journal of Global Warming | 0.782 | 19 | Switzerland | Inderscience Publishers | 2009, 2011-2014 | 26 | 0%  (0) | 0%  (0) | 11.5%  (3) | NA | NA | 12%  (3) | 0%  (0) | NA | 23.1%  (6) | 7.7%  (2) | 3.8%  (1) | 65.4%  (17) | NA | 65.4%  (17) | 30.8%  (8) | 3.8%  (1) | NA | NA |
| International Journal of Life Cycle Assessment | 4.307 | 105 | Germany | Springer Science + Business Media | 1996-2020 | 72 | 0%  (0) | 27.1%  (13) | 25.4%  (18) | NA | NA | 26%  (18) | 1%  (1) | NA | 9.7%  (7) | 1.4%  (1) | 4.2%  (3) | 84.7%  (61) | NA | 88.9%  (64) | 8.3%  (6) | 2.8%  (2) | NA | NA |
| International Journal of Mining Reclamation and Environment | 1.917 | 23 | United Kingdom | Taylor and Francis Ltd. | 2006-2020 | 38 | 0%  (0) | 0%  (0) | 10.8%  (4) | NA | NA | 10%  (4) | 3%  (1) | NA | 15.8%  (6) | 10.5%  (4) | NA | 73.7%  (28) | NA | 86.8%  (33) | 7.9%  (3) | 5.3%  (2) | NA | NA |
| International Journal of Phytoremediation | 2.528 | 84 | United Kingdom | Taylor and Francis Ltd. | 1994, 1996-2020 | 67 | 100%  (1) | 31.6%  (6) | 33.8%  (22) | NA | NA | 34%  (22) | 3%  (2) | 1.5%  (1) | 17.9%  (12) | 4.5%  (3) | 3%  (2) | 73.1%  (49) | NA | 76.1%  (51) | 14.9%  (10) | 9%  (6) | NA | NA |
| International Journal of Sediment Research | 2.577 | 37 | Netherlands | Elsevier BV | 1981-1987, 1991, 2000-2020 | 92 | 0%  (0) | 8%  (2) | 12.6%  (11) | NA | NA | 12%  (11) | 5%  (5) | 1.1%  (1) | 55.4%  (51) | 2.2%  (2) | 2.2%  (2) | 39.1%  (36) | NA | 51.1%  (47) | 41.3%  (38) | 7.6%  (7) | NA | NA |
| International Journal of Sustainable Transportation | 2.709 | 41 | United Kingdom | Taylor and Francis Ltd. | 2007-2020 | 45 | 0%  (0) | 0%  (0) | 23.3%  (10) | NA | NA | 24%  (10) | 4%  (2) | NA | 33.3%  (15) | NA | NA | 66.7%  (30) | NA | 95.6%  (43) | 4.4%  (2) | NA | NA | NA |
| International Journal of The Commons | 1.423 | 26 | United States | International Association for the Study of the Commons | 2011-2020 | 33 | 0%  (0) | 0%  (0) | 45.5%  (15) | NA | NA | 46%  (15) | 0%  (0) | 6.1%  (2) | 15.2%  (5) | NA | 3%  (1) | 75.8%  (25) | NA | 81.8%  (27) | 12.1%  (4) | 6.1%  (2) | NA | NA |
| International Journal of Urban Sciences | 1.831 | 20 | United Kingdom | Taylor and Francis Ltd. | 1998-2020 | 38 | 0%  (0) | 0%  (0) | 10.5%  (4) | NA | NA | 10%  (4) | 0%  (0) | NA | 44.7%  (17) | NA | NA | 55.3%  (21) | NA | 94.7%  (36) | 5.3%  (2) | NA | NA | NA |
| International Regional Science Review | 2.213 | 54 | United States | SAGE Publications Inc. | 1975-1984, 1986-2020 | 40 | 0%  (0) | 0%  (0) | 40%  (16) | NA | NA | 40%  (16) | 0%  (0) | NA | 5%  (2) | NA | 2.5%  (1) | 92.5%  (37) | NA | 97.5%  (39) | 2.5%  (1) | NA | NA | NA |
| International Soil and Water Conservation Research | 3.77 | 29 | Netherlands | NA | 2013-2020 | 78 | 0%  (0) | 23.4%  (11) | 18.4%  (14) | NA | NA | 18%  (14) | 3%  (2) | 1.3%  (1) | 44.9%  (35) | 3.8%  (3) | 6.4%  (5) | 43.6%  (34) | NA | 46.2%  (36) | 48.7%  (38) | 5.1%  (4) | NA | NA |
| Isotopes In Environmental and Health Studies | 1.652 | 41 | United Kingdom | Taylor and Francis Ltd. | 1995-2020 | 25 | 0%  (0) | 0%  (0) | 4%  (1) | NA | NA | 4%  (1) | 0%  (0) | NA | 8%  (2) | 8%  (2) | NA | 84%  (21) | NA | 92%  (23) | 8%  (2) | NA | NA | NA |
| Italian Journal of Agrometeorology-Rivista Italiana Di Agrometeorologia | 1.182 | 12 | Italy | Firenze University Press | 2008-2020 | 38 | NA | 25%  (4) | 18.4%  (7) | NA | NA | 18%  (7) | 0%  (0) | NA | 5.3%  (2) | 5.3%  (2) | 5.3%  (2) | 84.2%  (32) | NA | 89.5%  (34) | 7.9%  (3) | 2.6%  (1) | NA | NA |
| Journal of Aerosol Science | 2.649 | 110 | United Kingdom | Elsevier Ltd. | 1970-2021 | 30 | 0%  (0) | 0%  (0) | 30%  (9) | NA | NA | 30%  (9) | 0%  (0) | NA | 26.7%  (8) | NA | NA | 73.3%  (22) | NA | 80%  (24) | 16.7%  (5) | 3.3%  (1) | NA | NA |
| Journal of Agricultural & Environmental Ethics | 1.464 | 47 | Netherlands | Springer Netherlands | 1991-2020 | 14 | 100%  (1) | 100%  (1) | 35.7%  (5) | NA | NA | 36%  (5) | 0%  (0) | NA | NA | NA | NA | 100%  (14) | NA | 100%  (14) | NA | NA | NA | NA |
| Journal of Agricultural Education & Extension | 1.52 | 25 | United Kingdom | Taylor and Francis Ltd. | 2010-2020 | 25 | 50%  (1) | 63.6%  (7) | 44%  (11) | NA | NA | 44%  (11) | 0%  (0) | 12%  (3) | 8%  (2) | NA | 4%  (1) | 76%  (19) | NA | 76%  (19) | 12%  (3) | 12%  (3) | NA | NA |
| Journal of Applied Remote Sensing | 1.36 | 45 | United States | SPIE | 2007-2020 | 31 | 0%  (0) | 0%  (0) | 16.1%  (5) | NA | NA | 16%  (5) | 0%  (0) | 3.2%  (1) | 9.7%  (3) | NA | NA | 87.1%  (27) | NA | 83.9%  (26) | 12.9%  (4) | 3.2%  (1) | NA | NA |
| Journal of Architectural and Planning Research | 0.439 | 26 | United States | Locke Science Publishing Company Inc. | 1984-2019 | 157 | 0%  (0) | 30%  (3) | 24.8%  (39) | NA | NA | 24%  (39) | 0%  (0) | 0.6%  (1) | 2.5%  (4) | NA | NA | 96.8%  (152) | NA | 99.4%  (156) | 0.6%  (1) | NA | NA | NA |
| Journal of Arid Environments | 1.83 | 115 | United States | Academic Press Inc. | 1980-2021 | 45 | 0%  (0) | 30%  (3) | 25.6%  (11) | NA | NA | 26%  (11) | 4%  (2) | 17.8%  (8) | 8.9%  (4) | NA | 17.8%  (8) | 55.6%  (25) | NA | 60%  (27) | 40%  (18) | NA | NA | NA |
| Journal of Arid Land | 1.899 | 27 | China | Science Press | 2009-2020 | 69 | 0%  (0) | 0%  (0) | 7.5%  (5) | NA | NA | 8%  (5) | 3%  (2) | 2.9%  (2) | 68.1%  (47) | 5.8%  (4) | NA | 23.2%  (16) | NA | 24.6%  (17) | 62.3%  (43) | 13%  (9) | NA | NA |
| Journal of Atmospheric Chemistry | 1.521 | 70 | Netherlands | Springer Netherlands | 1983-2020 | 31 | 0%  (0) | 0%  (0) | 10.3%  (3) | NA | NA | 10%  (3) | 6%  (2) | NA | 25.8%  (8) | 3.2%  (1) | 3.2%  (1) | 67.7%  (21) | NA | 80.6%  (25) | 12.9%  (4) | 6.5%  (2) | NA | NA |
| Journal of Cleaner Production | 7.246 | 200 | United Kingdom | Elsevier Ltd. | 1993-2021 | 105 | 33.3%  (1) | 30%  (3) | 19%  (20) | NA | 25%  (4) | 20%  (20) | 0%  (0) | 1%  (1) | 39%  (41) | 9.5%  (10) | 5.7%  (6) | 44.8%  (47) | NA | 54.3%  (57) | 41.9%  (44) | 3.8%  (4) | NA | NA |
| Journal of Coastal Conservation | 1.374 | 39 | Netherlands | Springer Netherlands | 1995-2004, 2007-2020 | 16 | 0%  (0) | 0%  (0) | 12.5%  (2) | NA | NA | 12%  (2) | 0%  (0) | NA | 6.2%  (1) | 6.2%  (1) | NA | 87.5%  (14) | NA | 87.5%  (14) | 6.2%  (1) | 6.2%  (1) | NA | NA |
| Journal of Contaminant Hydrology | 2.347 | 100 | Netherlands | Elsevier | 1986-2020 | 73 | 25%  (1) | 25%  (1) | 9%  (6) | NA | NA | 8%  (6) | 8%  (6) | NA | 11%  (8) | NA | NA | 89%  (65) | NA | 94.5%  (69) | 5.5%  (4) | NA | NA | NA |
| Journal of Elementology | 0.71 | 23 | Poland | NA | 2008-2020 | 54 | 0%  (0) | 31.8%  (7) | 47.1%  (24) | NA | NA | 48%  (24) | 6%  (3) | NA | 3.7%  (2) | 74.1%  (40) | NA | 22.2%  (12) | NA | 96.3%  (52) | NA | 3.7%  (2) | NA | NA |
| Journal of Environment & Development | 1.844 | 42 | United States | SAGE Publications Inc. | 1992-2020 | 37 | 0%  (0) | 50%  (4) | 29.7%  (11) | NA | NA | 30%  (11) | 0%  (0) | 8.1%  (3) | 13.5%  (5) | NA | 8.1%  (3) | 70.3%  (26) | NA | 75.7%  (28) | 13.5%  (5) | 10.8%  (4) | NA | NA |
| Journal of Environmental Biology | 0.781 | 48 | India | Triveni Enterprises | 1988-2020 | 40 | NA  (0) | NA  (0) | 22.2%  (6) | 0%  (0) | NA | 20%  (6) | 25%  (10) | NA | 77.5%  (31) | 2.5%  (1) | 5%  (2) | 15%  (6) | NA | 15%  (6) | 12.5%  (5) | 72.5%  (29) | NA | NA |
| Journal of Environmental Economics and Management | 3.449 | 119 | United States | Academic Press Inc. | 1974-2020 | 65 | 0%  (0) | 20%  (2) | 20.3%  (13) | NA | NA | 20%  (13) | 2%  (1) | NA | 3.1%  (2) | NA | 1.5%  (1) | 95.4%  (62) | NA | 96.9%  (63) | 1.5%  (1) | 1.5%  (1) | NA | NA |
| Journal of Environmental Education | 2.103 | 56 | United Kingdom | Taylor and Francis Ltd. | 1971-2020 | 35 | 0%  (0) | 60%  (3) | 65.7%  (23) | NA | NA | 66%  (23) | 0%  (0) | NA | 8.6%  (3) | NA | 2.9%  (1) | 88.6%  (31) | NA | 94.3%  (33) | 2.9%  (1) | 2.9%  (1) | NA | NA |
| Journal of Environmental Engineering | 1.264 | 8 | Japan | Architectural Institute of Japan | 2008-2020 | 44 | 25%  (1) | 22.2%  (2) | 27.9%  (12) | NA | 30%  (3) | 28%  (12) | 2%  (1) | NA | 43.2%  (19) | NA | 18.2%  (8) | 38.6%  (17) | NA | 65.9%  (29) | 34.1%  (15) | NA | NA | NA |
| Journal of Environmental Engineering and Landscape Management | 2.733 | 28 | Lithuania | Vilnius Gediminas Technical University | 2004-2020 | 53 | 0%  (0) | 0%  (0) | 31.1%  (14) | 16.7%  (1) | NA | 30%  (15) | 4%  (2) | NA | 11.3%  (6) | 47.2%  (25) | NA | 41.5%  (22) | NA | 75.5%  (40) | 18.9%  (10) | 5.7%  (3) | NA | NA |
| Journal of Environmental Health Science and Engineering | 2.179 | 45 | Switzerland | Springer International Publishing AG | 2012-2020 | 26 | 100%  (1) | 40%  (4) | 16.7%  (4) | NA | NA | 16%  (4) | 8%  (2) | 3.8%  (1) | 84.6%  (22) | NA | NA | 11.5%  (3) | NA | 11.5%  (3) | 3.8%  (1) | 84.6%  (22) | NA | NA |
| Journal of Environmental Informatics | 4.604 | 31 | Canada | International Society for Environmental Information Sciences | 2007-2020 | 29 | 0%  (0) | 0%  (0) | 13.8%  (4) | NA | NA | 14%  (4) | 0%  (0) | NA | 27.6%  (8) | NA | NA | 72.4%  (21) | NA | 79.3%  (23) | 20.7%  (6) | NA | NA | NA |
| Journal of Environmental Law | 1.633 | 28 | United Kingdom | Oxford University Press | 1989-2020 | 33 | 100%  (1) | 100%  (1) | 63.2%  (12) | 35.7%  (5) | NA | 52%  (17) | 0%  (0) | 3%  (1) | 9.1%  (3) | NA | NA | 87.9%  (29) | NA | 93.9%  (31) | 6.1%  (2) | NA | NA | NA |
| Journal of Environmental Management | 5.647 | 179 | United States | Academic Press Inc. | 1970, 1973, 1975, 1977-2021 | 61 | 0%  (0) | 28.6%  (6) | 16.7%  (9) | NA | NA | 16%  (9) | 11%  (7) | 6.6%  (4) | 32.8%  (20) | NA | 1.6%  (1) | 59%  (36) | NA | 67.2%  (41) | 31.1%  (19) | 1.6%  (1) | NA | NA |
| Journal of Environmental Protection and Ecology | 0.692 | 20 | Bulgaria | Scibulcom Ltd. | 2008-2020 | 45 | 0%  (0) | 8.3%  (1) | 21.1%  (8) | NA | NA | 22%  (8) | 16%  (7) | NA | NA | 44.4%  (20) | NA | 55.6%  (25) | NA | 53.3%  (24) | 44.4%  (20) | 2.2%  (1) | NA | NA |
| Journal of Environmental Psychology | 3.301 | 137 | United States | Academic Press Inc. | 1981-2020 | 72 | 0%  (0) | 0%  (0) | 40%  (2) | 28.4%  (19) | NA | 30%  (21) | 0%  (0) | NA | 2.8%  (2) | NA | 1.4%  (1) | 95.8%  (69) | NA | 98.6%  (71) | 1.4%  (1) | NA | NA | NA |
| Journal of Environmental Quality | 2.142 | 171 | United States | ASA/CSSA/SSSA | 1972-2020 | 62 | 0%  (0) | 0%  (0) | 26.2%  (16) | NA | NA | 26%  (16) | 2%  (1) | NA | 8.1%  (5) | 3.2%  (2) | NA | 88.7%  (55) | NA | 93.5%  (58) | 6.5%  (4) | NA | NA | NA |
| Journal of Environmental Radioactivity | 2.161 | 92 | United Kingdom | Elsevier Ltd. | 1984-2020 | 30 | 0%  (0) | 16.7%  (2) | 26.7%  (8) | NA | NA | 26%  (8) | 0%  (0) | 3.3%  (1) | 13.3%  (4) | 6.7%  (2) | 3.3%  (1) | 73.3%  (22) | NA | 83.3%  (25) | 10%  (3) | 6.7%  (2) | NA | NA |
| Journal of Environmental Science and Health Part A-Toxic/Hazardous Substances & Environmental Engineering | 1.724 | 71 | United States | Taylor and Francis Ltd. | 1978-1979, 1987, 1996-2020 | 25 | 0%  (0) | 0%  (0) | 8.3%  (2) | NA | NA | 8%  (2) | 4%  (1) | 4%  (1) | 8%  (2) | 4%  (1) | 8%  (2) | 76%  (19) | NA | 84%  (21) | 16%  (4) | NA | NA | NA |
| Journal of Environmental Science and Health Part C-Environmental Carcinogenesis & Ecotoxicology Reviews | 2.552 | 51 | United States | Taylor and Francis Inc. | 2020 | 31 | 0%  (0) | 50%  (1) | 14.3%  (4) | NA | NA | 14%  (4) | 10%  (3) | 3.2%  (1) | 19.4%  (6) | NA | NA | 77.4%  (24) | NA | 80.6%  (25) | 16.1%  (5) | 3.2%  (1) | NA | NA |
| Journal of Environmental Science and Management | 0.42 | 9 | Philippines | University of the Philippines Los Banos | 2011-2020 | 11 | 0%  (0) | 0%  (0) | 27.3%  (3) | NA | NA | 28%  (3) | 0%  (0) | NA | 81.8%  (9) | NA | NA | 18.2%  (2) | NA | 27.3%  (3) | 18.2%  (2) | 54.5%  (6) | NA | NA |
| Journal of Environmental Sciences | 4.302 | 99 | China | Chinese Academy of Sciences | 1970, 1972-1973, 1978-1985, 1993, 1995-2021 | 76 | 0%  (0) | 0%  (0) | 7.6%  (5) | 14.3%  (1) | NA | 8%  (6) | 4%  (3) | NA | 65.8%  (50) | 1.3%  (1) | NA | 32.9%  (25) | NA | 43.4%  (33) | 56.6%  (43) | NA | NA | NA |
| Journal of Flood Risk Management | 3.066 | 36 | Denmark | Blackwell Publishing | 2009-2020 | 52 | 0%  (0) | 66.7%  (4) | 25.5%  (13) | NA | NA | 26%  (13) | 2%  (1) | NA | 15.4%  (8) | 1.9%  (1) | NA | 82.7%  (43) | NA | 88.5%  (46) | 9.6%  (5) | 1.9%  (1) | NA | NA |
| Journal of Geophysical Research-Biogeosciences | 3.408 | 298 | United States | Wiley-Blackwell | 1956, 1959-2020 | 42 | 100%  (1) | 50%  (2) | 39%  (16) | NA | NA | 40%  (16) | 2%  (1) | 2.4%  (1) | 4.8%  (2) | NA | NA | 92.9%  (39) | NA | 92.9%  (39) | 7.1%  (3) | NA | NA | NA |
| Journal of Great Lakes Research | 1.933 | 78 | United States | International Association of Great Lakes Research | 1970, 1975-2020 | 34 | 0%  (0) | 0%  (0) | 22.6%  (7) | NA | NA | 22%  (7) | 9%  (3) | 2.9%  (1) | 2.9%  (1) | NA | NA | 94.1%  (32) | NA | 94.1%  (32) | NA | 2.9%  (1) | 2.9%  (1) | NA |
| Journal of Hazardous Materials | 9.038 | 284 | Netherlands | Elsevier | 1975-2021 | 93 | 25%  (1) | 36.4%  (8) | 24.1%  (20) | NA | 40%  (4) | 24%  (20) | 11%  (10) | NA | 41.9%  (39) | 2.2%  (2) | 2.2%  (2) | 53.8%  (50) | NA | 64.5%  (60) | 30.1%  (28) | 5.4%  (5) | NA | NA |
| Journal of Housing and The Built Environment | 1.442 | 44 | Netherlands | Springer Netherlands | 1996-2020 | 35 | 0%  (0) | 50%  (1) | 47.1%  (8) | 16.7%  (3) | NA | 32%  (11) | 0%  (0) | 5.7%  (2) | 11.4%  (4) | 8.6%  (3) | NA | 74.3%  (26) | NA | 85.7%  (30) | 11.4%  (4) | 2.9%  (1) | NA | NA |
| Journal of Hydro-Environment Research | 2.099 | 38 | Netherlands | Elsevier | 2007-2020 | 53 | 0%  (0) | 0%  (0) | 6.5%  (2) | 0%  (0) | NA | 4%  (2) | 15%  (8) | NA | 77.4%  (41) | NA | NA | 22.6%  (12) | NA | 56.6%  (30) | 30.2%  (16) | 13.2%  (7) | NA | NA |
| Journal of Hydroinformatics | 1.728 | 50 | United Kingdom | IWA Publishing | 1999-2020 | 44 | 0%  (0) | 21.4%  (3) | 11.4%  (5) | NA | NA | 12%  (5) | 0%  (0) | NA | 20.5%  (9) | 2.3%  (1) | 2.3%  (1) | 75%  (33) | NA | 88.6%  (39) | 11.4%  (5) | NA | NA | NA |
| Journal of Hydrologic Engineering | 1.594 | 89 | United States | American Society of Civil Engineers  (ASCE) | 1996-2020 | 82 | 0%  (0) | 0%  (0) | 9.6%  (5) | 4%  (1) | 25%  (1) | 8%  (6) | 6%  (5) | 1.2%  (1) | 19.5%  (16) | 3.7%  (3) | 1.2%  (1) | 74.4%  (61) | NA | 80.5%  (66) | 14.6%  (12) | 4.9%  (4) | NA | NA |
| Journal of Industrial Ecology | 6.539 | 102 | United States | Wiley-Blackwell | 1997-2020 | 73 | 0%  (0) | 28.6%  (10) | 20.8%  (15) | NA | NA | 20%  (15) | 1%  (1) | 1.4%  (1) | 13.7%  (10) | NA | NA | 83.6%  (61) | 1.4%  (1) | 89%  (65) | 9.6%  (7) | NA | NA | 1.4%  (1) |
| Journal of Integrative Environmental Sciences | 2.667 | 20 | United Kingdom | Taylor and Francis Ltd. | 2010-2020 | 18 | 0%  (0) | 0%  (0) | 11.1%  (2) | NA | NA | 12%  (2) | 0%  (0) | 5.6%  (1) | 22.2%  (4) | 11.1%  (2) | NA | 61.1%  (11) | NA | 77.8%  (14) | 11.1%  (2) | 11.1%  (2) | NA | NA |
| Journal of Material Cycles and Waste Management | 1.974 | 41 | Japan | Springer Japan | 2002, 2005-2020 | 83 | 0%  (0) | 0%  (0) | 11%  (8) | NA | NA | 10%  (8) | 12%  (10) | NA | 77.1%  (64) | NA | NA | 22.9%  (19) | NA | 84.3%  (70) | 9.6%  (8) | 6%  (5) | NA | NA |
| Journal of Mountain Science | 1.55 | 34 | China | Science Press | 2007-2020 | 128 | 50%  (1) | 40%  (2) | 7.8%  (8) | 0%  (0) | NA | 8%  (8) | 16%  (21) | 0.8%  (1) | 57.8%  (74) | 3.1%  (4) | 1.6%  (2) | 36.7%  (47) | NA | 41.4%  (53) | 52.3%  (67) | 5.5%  (7) | 0.8%  (1) | NA |
| Journal of Paleolimnology | 1.639 | 84 | Netherlands | Springer Netherlands | 1988-2020 | 37 | 0%  (0) | 0%  (0) | 26.3%  (5) | 16.7%  (3) | NA | 22%  (8) | 0%  (0) | NA | 5.4%  (2) | NA | 5.4%  (2) | 89.2%  (33) | NA | 89.2%  (33) | 10.8%  (4) | NA | NA | NA |
| Journal of Regional Science | 2.088 | 79 | United Kingdom | Wiley-Blackwell Publishing Ltd | 1958-2020 | 66 | 25%  (1) | 25%  (1) | 17.9%  (10) | 0%  (0) | NA | 16%  (10) | 0%  (0) | NA | 7.6%  (5) | NA | NA | 92.4%  (61) | NA | 98.5%  (65) | 1.5%  (1) | NA | NA | NA |
| Journal of Soil Science and Plant Nutrition | 2.156 | 39 | Chile | Sociedad Chilena de la Ciencia del Suelo | 2010-2020 | 32 | 100%  (1) | 50%  (4) | 25%  (8) | NA | NA | 24%  (8) | 0%  (0) | NA | 9.4%  (3) | 6.2%  (2) | 37.5%  (12) | 46.9%  (15) | NA | 87.5%  (28) | 6.2%  (2) | 6.2%  (2) | NA | NA |
| Journal of Soils and Sediments | 2.763 | 73 | Germany | Springer Science + Business Media | 2001-2020 | 101 | 0%  (0) | 22.7%  (22) | 22.7%  (22) | NA | NA | 22%  (22) | 4%  (4) | NA | 29.7%  (30) | 4%  (4) | 3%  (3) | 63.4%  (64) | NA | 69.3%  (70) | 28.7%  (29) | 2%  (2) | NA | NA |
| Journal of The Air & Waste Management Association | 2.245 | 89 | United Kingdom | Taylor and Francis Ltd. | 1989-2020 | 41 | NA  (0) | 36%  (9) | 35.9%  (14) | NA | NA | 36%  (14) | 5%  (2) | NA | 17.1%  (7) | NA | NA | 82.9%  (34) | NA | 90.2%  (37) | 4.9%  (2) | 4.9%  (2) | NA | NA |
| Journal of The Association of Environmental and Resource Economists | 5.167 | 22 | United States | University of Chicago Press | 2015-2020 | 61 | 50%  (2) | 27.8%  (5) | 18%  (11) | NA | NA | 18%  (11) | 0%  (0) | NA | 1.6%  (1) | NA | NA | 98.4%  (60) | NA | 98.4%  (60) | 1.6%  (1) | NA | NA | NA |
| Journal of The Indian Society of Remote Sensing | 0.997 | 38 | India | Springer India | 1973-2020 | 20 | 0%  (0) | 10.5%  (2) | 10.5%  (2) | NA | NA | 10%  (2) | 5%  (1) | NA | 100%  (20) | NA | NA | NA | NA | NA | 5%  (1) | 95%  (19) | NA | NA |
| Journal of Water and Health | 1.349 | 59 | United Kingdom | IWA Publishing | 2003-2020 | 37 | 0%  (0) | 33.3%  (3) | 18.9%  (7) | NA | NA | 18%  (7) | 0%  (0) | 2.7%  (1) | 10.8%  (4) | 2.7%  (1) | NA | 83.8%  (31) | NA | 97.3%  (36) | 2.7%  (1) | NA | NA | NA |
| Land | 2.429 | 23 | Switzerland | MDPI Multidisciplinary Digital Publishing Institute | 2012-2020 | 234 | 0%  (0) | 50%  (4) | 26.2%  (59) | 14.3%  (1) | NA | 26%  (60) | 1%  (2) | 1.7%  (4) | 13.2%  (31) | 4.3%  (10) | 2.6%  (6) | 78.2%  (183) | NA | 85.5%  (200) | 12.8%  (30) | 1.7%  (4) | NA | NA |
| Land Degradation & Development | 3.775 | 81 | United Kingdom | John Wiley and Sons Ltd | 1989-1990, 1992-2020 | 67 | 100%  (1) | 25%  (2) | 25%  (16) | NA | NA | 24%  (16) | 4%  (3) | 3%  (2) | 35.8%  (24) | 7.5%  (5) | 3%  (2) | 50.7%  (34) | NA | 58.2%  (39) | 34.3%  (23) | 7.5%  (5) | NA | NA |
| Land Economics | 1.62 | 86 | United States | University of Wisconsin Press | 1964, 1973-1974, 1976-2020 | 32 | 0%  (0) | 0%  (0) | 37.5%  (12) | NA | NA | 38%  (12) | 0%  (0) | NA | NA | 3.1%  (1) | NA | 96.9%  (31) | NA | 100%  (32) | NA | NA | NA | NA |
| Land Use Policy | 3.682 | 115 | United Kingdom | Elsevier Ltd. | 1984-2021 | 54 | 0%  (0) | 40%  (2) | 21.2%  (11) | NA | NA | 22%  (11) | 4%  (2) | 3.7%  (2) | 9.3%  (5) | NA | 5.6%  (3) | 81.5%  (44) | NA | 85.2%  (46) | 14.8%  (8) | NA | NA | NA |
| Landscape and Urban Planning | 5.441 | 161 | Netherlands | Elsevier | 1986-2020 | 69 | 50%  (1) | 50%  (1) | 40%  (4) | 22.2%  (12) | NA | 24%  (16) | 7%  (5) | 1.4%  (1) | 11.6%  (8) | NA | 1.4%  (1) | 85.5%  (59) | NA | 89.9%  (62) | 10.1%  (7) | NA | NA | NA |
| Landscape Research | 1.806 | 45 | United Kingdom | Routledge | 1970, 1972-2020 | 53 | 100%  (1) | 100%  (1) | 62.5%  (5) | 51.1%  (23) | NA | 52%  (28) | 0%  (0) | NA | 13.2%  (7) | NA | 3.8%  (2) | 83%  (44) | NA | 90.6%  (48) | 9.4%  (5) | NA | NA | NA |
| Local Environment | 1.856 | 62 | United Kingdom | Routledge | 1996-2020 | 49 | 0%  (0) | 0%  (0) | 25%  (1) | 48.9%  (22) | NA | 46%  (23) | 0%  (0) | 4.1%  (2) | 6.1%  (3) | NA | 2%  (1) | 87.8%  (43) | NA | 91.8%  (45) | 6.1%  (3) | 2%  (1) | NA | NA |
| Marine Environmental Research | 2.727 | 96 | Netherlands | Elsevier BV | 1978-2020 | 30 | 50%  (1) | 50%  (1) | 20%  (6) | NA | NA | 20%  (6) | 0%  (0) | NA | 10%  (3) | 3.3%  (1) | NA | 86.7%  (26) | NA | 90%  (27) | 6.7%  (2) | 3.3%  (1) | NA | NA |
| Marine Policy | 3.228 | 95 | United Kingdom | Elsevier Ltd. | 1977-2020 | 61 | 0%  (0) | 50%  (2) | 26.7%  (16) | NA | NA | 26%  (16) | 2%  (1) | 1.6%  (1) | 14.8%  (9) | 1.6%  (1) | 4.9%  (3) | 77%  (47) | NA | 86.9%  (53) | 11.5%  (7) | 1.6%  (1) | NA | NA |
| Marine Pollution Bulletin | 4.049 | 179 | United Kingdom | Elsevier Inc. | 1970-2020 | 26 | 33.3%  (1) | 21.4%  (3) | 12%  (3) | NA | NA | 12%  (3) | 4%  (1) | 3.8%  (1) | 23.1%  (6) | NA | 3.8%  (1) | 69.2%  (18) | NA | 69.2%  (18) | 26.9%  (7) | 3.8%  (1) | NA | NA |
| Marine Resource Economics | 2.868 | 45 | United States | University of Chicago Press | 1987, 1989, 1993-2020 | 17 | 0%  (0) | 0%  (0) | 11.1%  (1) | 0%  (0) | NA | 6%  (1) | 6%  (1) | NA | NA | NA | 5.9%  (1) | 94.1%  (16) | NA | 100%  (17) | NA | NA | NA | NA |
| Microbial Risk Analysis | 2.182 | 12 | Netherlands | Elsevier BV | 2016-2020 | 26 | 0%  (0) | 0%  (0) | 36%  (9) | NA | NA | 36%  (9) | 4%  (1) | NA | NA | 3.8%  (1) | NA | 96.2%  (25) | NA | 96.2%  (25) | 3.8%  (1) | NA | NA | NA |
| Mires and Peat | 1.328 | 13 | Germany | The International Mire Conservation Group  (IMCG) | 2015-2020 | 43 | 100%  (2) | 100%  (2) | 30.2%  (13) | NA | NA | 30%  (13) | 0%  (0) | 4.7%  (2) | 9.3%  (4) | 9.3%  (4) | NA | 76.7%  (33) | NA | 86%  (37) | 9.3%  (4) | 4.7%  (2) | NA | NA |
| Mitigation and Adaptation Strategies For Global Change | 3.23 | 71 | Netherlands | Springer Netherlands | 1996-2020 | 29 | 0%  (0) | 0%  (0) | 0%  (0) | 21.4%  (6) | NA | 20%  (6) | 0%  (0) | NA | 10.3%  (3) | 6.9%  (2) | NA | 82.8%  (24) | NA | 86.2%  (25) | 6.9%  (2) | 6.9%  (2) | NA | NA |
| Mountain Research and Development | 1.494 | 57 | Switzerland | International Mountain Society | 1981-2019 | 48 | 0%  (0) | 60%  (3) | 40.4%  (19) | NA | NA | 40%  (19) | 2%  (1) | 8.3%  (4) | 22.9%  (11) | NA | 4.2%  (2) | 64.6%  (31) | NA | 64.6%  (31) | 18.8%  (9) | 14.6%  (7) | 2.1%  (1) | NA |
| Nanoimpact | 5.478 | 25 | Netherlands | Elsevier BV | 2016-2020 | 20 | 33.3%  (1) | 8.3%  (1) | 5%  (1) | NA | NA | 4%  (1) | 0%  (0) | NA | 15%  (3) | NA | NA | 85%  (17) | NA | 90%  (18) | 10%  (2) | NA | NA | NA |
| Natural Hazards Review | 1.667 | 57 | United States | American Society of Civil Engineers  (ASCE) | 2000-2020 | 59 | 50%  (1) | 50%  (1) | 35.1%  (20) | NA | 33.3%  (3) | 36%  (20) | 3%  (2) | NA | 13.6%  (8) | NA | NA | 86.4%  (51) | NA | 86.4%  (51) | 11.9%  (7) | 1.7%  (1) | NA | NA |
| Natural Resource Modeling | 0.915 | 32 | United States | Wiley-Blackwell | 1990, 1992-1995, 1997-2020 | 29 | 100%  (1) | 33.3%  (6) | 33.3%  (6) | 30%  (3) | NA | 32%  (9) | 3%  (1) | NA | 6.9%  (2) | NA | 3.4%  (1) | 89.7%  (26) | NA | 89.7%  (26) | 6.9%  (2) | 3.4%  (1) | NA | NA |
| Natural Resources Forum | 1.436 | 49 | United Kingdom | Wiley-Blackwell Publishing Ltd | 1976-2020 | 15 | 0%  (0) | 50%  (1) | 42.9%  (3) | 42.9%  (3) | NA | 42%  (6) | 7%  (1) | NA | NA | NA | NA | 93.3%  (14) | 6.7%  (1) | 93.3%  (14) | NA | NA | NA | 6.7%  (1) |
| Nature and Culture | 0.613 | 19 | United States | Berghahn Journals | 2008-2020 | 23 | 0%  (0) | 0%  (0) | 21.7%  (5) | NA | NA | 22%  (5) | 0%  (0) | 4.3%  (1) | 13%  (3) | NA | NA | 82.6%  (19) | NA | 87%  (20) | 4.3%  (1) | 8.7%  (2) | NA | NA |
| Nature Climate Change | 20.893 | 189 | United Kingdom | Nature Publishing Group | 2011-2020 | 14 | 100%  (1) | 100%  (1) | 75%  (3) | 30%  (3) | NA | 42%  (6) | 0%  (0) | NA | NA | NA | 7.1%  (1) | 92.9%  (13) | NA | 92.9%  (13) | 7.1%  (1) | NA | NA | NA |
| Nature Sustainability | 12.08 | 37 | United Kingdom | Nature Publishing Group | 2018-2020 | 31 | 100%  (1) | 50%  (2) | 40%  (2) | 42.3%  (11) | NA | 42%  (13) | 0%  (0) | NA | 6.5%  (2) | NA | NA | 93.5%  (29) | NA | 96.8%  (30) | 3.2%  (1) | NA | NA | NA |
| Npj Clean Water | 4.87 | 15 | United States | Springer Nature | 2018-2020 | 48 | 0%  (0) | 28.6%  (2) | 31.2%  (15) | NA | NA | 32%  (15) | 0%  (0) | 8.3%  (4) | 43.8%  (21) | NA | 2.1%  (1) | 45.8%  (22) | NA | 81.2%  (39) | 10.4%  (5) | 8.3%  (4) | NA | NA |
| Open House International | 0.183 | 11 | United Kingdom | Open House International Association | 2007-2020 | 42 | 50%  (1) | 50%  (1) | 54.8%  (23) | NA | NA | 54%  (23) | 0%  (0) | 4.8%  (2) | 31%  (13) | 4.8%  (2) | 4.8%  (2) | 54.8%  (23) | NA | 66.7%  (28) | 26.2%  (11) | 7.1%  (3) | NA | NA |
| Organization & Environment | 3.333 | 60 | United States | SAGE Publications Inc. | 1987-2020 | 89 | 0%  (0) | 31.2%  (5) | 41.6%  (37) | NA | NA | 42%  (37) | 0%  (0) | NA | 7.9%  (7) | NA | NA | 92.1%  (82) | NA | 97.8%  (87) | 2.2%  (2) | NA | NA | NA |
| Ozone-Science & Engineering | 2.082 | 49 | United Kingdom | Taylor and Francis Ltd. | 1979-2020 | 37 | 0%  (0) | 0%  (0) | 8.6%  (3) | NA | NA | 8%  (3) | 5%  (2) | NA | 27%  (10) | 8.1%  (3) | 5.4%  (2) | 59.5%  (22) | NA | 86.5%  (32) | 10.8%  (4) | 2.7%  (1) | NA | NA |
| Papers In Regional Science | 2.22 | 64 | United States | Wiley-Blackwell | 1955-2020 | 50 | 100%  (1) | 50%  (3) | 20%  (10) | NA | NA | 20%  (10) | 0%  (0) | NA | 12%  (6) | NA | NA | 88%  (44) | NA | 100%  (50) | NA | NA | NA | NA |
| Physical Geography | 1.435 | 40 | United Kingdom | Taylor and Francis Ltd. | 1975, 1980-2020 | 25 | 0%  (0) | 0%  (0) | 20%  (5) | NA | NA | 20%  (5) | 0%  (0) | NA | 8%  (2) | NA | NA | 92%  (23) | NA | 96%  (24) | 4%  (1) | NA | NA | NA |
| Polar Record | 0.84 | 32 | United Kingdom | Cambridge University Press | 1931-2020 | 25 | 0%  (0) | 0%  (0) | 0%  (0) | 39.1%  (9) | NA | 36%  (9) | 0%  (0) | 8%  (2) | 8%  (2) | NA | NA | 84%  (21) | NA | 88%  (22) | 8%  (2) | 4%  (1) | NA | NA |
| Polish Journal of Environmental Studies | 1.383 | 54 | Poland | HARD | 1996-2020 | 54 | NA | NA | 30.2%  (16) | NA | NA | 30%  (16) | 2%  (1) | NA | 14.8%  (8) | 51.9%  (28) | 9.3%  (5) | 24.1%  (13) | NA | 72.2%  (39) | 20.4%  (11) | 7.4%  (4) | NA | NA |
| Population and Environment | 2.439 | 50 | Netherlands | Springer Netherlands | 1980-1985, 1987-2020 | 17 | 100%  (1) | 100%  (1) | 100%  (1) | 31.2%  (5) | NA | 36%  (6) | 0%  (0) | 5.9%  (1) | 5.9%  (1) | 5.9%  (1) | NA | 82.4%  (14) | NA | 94.1%  (16) | NA | 5.9%  (1) | NA | NA |
| Problemy Ekorozwoju | 1.177 | 20 | Poland | Politechnika Lubelska | 2008-2020 | 40 | 0%  (0) | 0%  (0) | 7.9%  (3) | NA | NA | 8%  (3) | 5%  (2) | NA | 10%  (4) | 62.5%  (25) | 2.5%  (1) | 25%  (10) | NA | 87.5%  (35) | 10%  (4) | 2.5%  (1) | NA | NA |
| Progress In Planning | 2.563 | 48 | United Kingdom | Elsevier Ltd. | 1969, 1973-2020 | 28 | 0%  (0) | 0%  (0) | 36.4%  (8) | NA | NA | 36%  (8) | 21%  (6) | 3.6%  (1) | 7.1%  (2) | NA | 3.6%  (1) | 78.6%  (22) | 7.1%  (2) | 82.1%  (23) | 10.7%  (3) | NA | NA | 7.1%  (2) |
| Radiation and Environmental Biophysics | 1.321 | 54 | Germany | Springer New York | 1974-2020 | 22 | 33.3%  (1) | 14.3%  (1) | 22.7%  (5) | NA | NA | 22%  (5) | 0%  (0) | NA | 18.2%  (4) | 9.1%  (2) | NA | 72.7%  (16) | NA | 81.8%  (18) | 13.6%  (3) | 4.5%  (1) | NA | NA |
| Rangeland Ecology & Management | 2.095 | 70 | United States | Elsevier Inc. | 2005-2020 | 26 | 0%  (0) | 0%  (0) | 30.8%  (8) | NA | NA | 30%  (8) | 0%  (0) | NA | NA | 3.8%  (1) | NA | 96.2%  (25) | NA | 96.2%  (25) | 3.8%  (1) | NA | NA | NA |
| Regional Environmental Change | 3.481 | 62 | Germany | Springer Verlag | 2005-2020 | 30 | 0%  (0) | 0%  (0) | 40%  (12) | NA | NA | 40%  (12) | 0%  (0) | 6.7%  (2) | 16.7%  (5) | 3.3%  (1) | 3.3%  (1) | 70%  (21) | NA | 76.7%  (23) | 16.7%  (5) | 6.7%  (2) | NA | NA |
| Regional Science and Urban Economics | 1.667 | 79 | Netherlands | Elsevier | 1973-2020 | 63 | 0%  (0) | 12.5%  (1) | 11.9%  (7) | NA | NA | 12%  (7) | 6%  (4) | NA | 9.5%  (6) | NA | NA | 90.5%  (57) | NA | 96.8%  (61) | 3.2%  (2) | NA | NA | NA |
| Regional Studies | 3.312 | 120 | United Kingdom | Routledge | 1967-2020 | 36 | 0%  (0) | 35.3%  (6) | 33.3%  (6) | 11.1%  (2) | 0%  (0) | 22%  (8) | 0%  (0) | NA | 5.6%  (2) | NA | 2.8%  (1) | 91.7%  (33) | NA | 94.4%  (34) | 5.6%  (2) | NA | NA | NA |
| Remote Sensing of Environment | 9.085 | 281 | United States | Elsevier Inc. | 1969-2020 | 75 | 33.3%  (1) | 19.2%  (5) | 9.7%  (7) | NA | NA | 10%  (7) | 4%  (3) | NA | 20%  (15) | 1.3%  (1) | 1.3%  (1) | 77.3%  (58) | NA | 82.7%  (62) | 17.3%  (13) | NA | NA | NA |
| Resource and Energy Economics | 1.829 | 70 | Netherlands | Elsevier | 1993-2020 | 28 | 0%  (0) | 0%  (0) | 21.4%  (6) | NA | NA | 22%  (6) | 0%  (0) | NA | 3.6%  (1) | NA | NA | 96.4%  (27) | NA | 96.4%  (27) | NA | 3.6%  (1) | NA | NA |
| Resources Conservation and Recycling | 8.086 | 130 | Netherlands | Elsevier | 1988-2021 | 73 | 0%  (0) | 32%  (8) | 19.2%  (14) | NA | NA | 20%  (14) | 0%  (0) | NA | 30.1%  (22) | 2.7%  (2) | 4.1%  (3) | 63%  (46) | NA | 72.6%  (53) | 23.3%  (17) | 4.1%  (3) | NA | NA |
| Resources Policy | 3.986 | 69 | United Kingdom | Elsevier Ltd. | 1974-2020 | 34 | 0%  (0) | 9.1%  (1) | 12.1%  (4) | NA | NA | 12%  (4) | 3%  (1) | 8.8%  (3) | 11.8%  (4) | NA | 5.9%  (2) | 73.5%  (25) | NA | 79.4%  (27) | 14.7%  (5) | 2.9%  (1) | 2.9%  (1) | NA |
| Review of Environmental Economics and Policy | 6.487 | 53 | United States | Oxford University Press | 2008-2019 | 38 | 100%  (1) | 50%  (2) | 44.7%  (17) | NA | NA | 44%  (17) | 0%  (0) | NA | NA | NA | 2.6%  (1) | 97.4%  (37) | NA | 97.4%  (37) | 2.6%  (1) | NA | NA | NA |
| Review of European Comparative & International Environmental Law | 1.054 | 18 | United Kingdom | John Wiley and Sons Ltd | 2013-2020 | 34 | 0%  (0) | 66.7%  (2) | 29.4%  (10) | NA | NA | 30%  (10) | 0%  (0) | 2.9%  (1) | 5.9%  (2) | NA | NA | 91.2%  (31) | NA | 91.2%  (31) | 8.8%  (3) | NA | NA | NA |
| Reviews In Environmental Science and Bio-Technology | 4.957 | 79 | Netherlands | Springer Netherlands | 2002-2020 | 21 | 0%  (0) | 0%  (0) | 4.8%  (1) | NA | NA | 4%  (1) | 0%  (0) | 14.3%  (3) | 19%  (4) | NA | 19%  (4) | 47.6%  (10) | NA | 57.1%  (12) | 33.3%  (7) | 4.8%  (1) | 4.8%  (1) | NA |
| Reviews of Environmental Contamination and Toxicology | 5.767 | 80 | United States | Springer New York | 1987-2020 | 6 | 0%  (0) | 0%  (0) | 33.3%  (2) | NA | NA | 34%  (2) | 0%  (0) | NA | NA | NA | 16.7%  (1) | 83.3%  (5) | NA | 100%  (6) | NA | NA | NA | NA |
| Revista Internacional De Contaminacion Ambiental | 0.521 | 19 | Mexico | Centro de Ciencias de la Atmosfera, UNAM | 1996-2020 | 37 | 100%  (1) | 44.4%  (12) | 43.2%  (16) | NA | NA | 44%  (16) | 0%  (0) | NA | NA | 2.7%  (1) | 86.5%  (32) | 10.8%  (4) | NA | 10.8%  (4) | 89.2%  (33) | NA | NA | NA |
| River Research and Applications | 1.916 | 94 | United Kingdom | John Wiley and Sons Ltd | 1996-2020 | 34 | 0%  (0) | 25%  (1) | 17.6%  (6) | NA | 100%  (2) | 18%  (6) | 0%  (0) | NA | 17.6%  (6) | NA | 2.9%  (1) | 79.4%  (27) | NA | 85.3%  (29) | 11.8%  (4) | 2.9%  (1) | NA | NA |
| Rocznik Ochrona Srodowiska | 0.804 | 16 | Poland | Middle Pomeranian Scientific Society | 2007-2019 | 28 | 0%  (0) | 0%  (0) | 21.4%  (6) | NA | NA | 22%  (6) | 0%  (0) | NA | NA | 96.4%  (27) | NA | 3.6%  (1) | NA | 92.9%  (26) | 7.1%  (2) | NA | NA | NA |
| Sar and Qsar In Environmental Research | 2.053 | 48 | United Kingdom | Taylor and Francis Ltd. | 1993-2020 | 26 | 0%  (0) | 0%  (0) | 0%  (0) | 37.5%  (9) | NA | 36%  (9) | 4%  (1) | NA | 11.5%  (3) | 23.1%  (6) | NA | 65.4%  (17) | NA | 73.1%  (19) | 19.2%  (5) | 7.7%  (2) | NA | NA |
| Science and Public Policy | 1.73 | 65 | United Kingdom | Oxford University Press | 1974, 1976-1982, 1984-2020 | 39 | 33.3%  (1) | 33.3%  (1) | 33.3%  (1) | 30.6%  (11) | NA | 30%  (12) | 0%  (0) | 2.6%  (1) | 17.9%  (7) | 2.6%  (1) | 7.7%  (3) | 69.2%  (27) | NA | 84.6%  (33) | 15.4%  (6) | NA | NA | NA |
| Science of The Total Environment | 6.551 | 244 | Netherlands | Elsevier | 1970, 1972-2021 | 219 | 0%  (0) | 30%  (15) | 23.4%  (51) | NA | NA | 24%  (51) | 0%  (1) | NA | 31.1%  (68) | 3.2%  (7) | 3.2%  (7) | 62.6%  (137) | NA | 70.8%  (155) | 27.4%  (60) | 1.8%  (4) | NA | NA |
| Society & Natural Resources | 1.813 | 87 | United Kingdom | Taylor and Francis Ltd. | 1988-2020 | 106 | 100%  (2) | 56.6%  (30) | 46.7%  (49) | NA | NA | 46%  (49) | 1%  (1) | 3.8%  (4) | 5.7%  (6) | 0.9%  (1) | 1.9%  (2) | 87.7%  (93) | NA | 91.5%  (97) | 5.7%  (6) | 2.8%  (3) | NA | NA |
| Soil & Sediment Contamination | 1.25 | 47 | United Kingdom | Taylor and Francis Ltd. | 1996-2020 | 48 | 0%  (0) | 0%  (0) | 12.8%  (6) | NA | NA | 12%  (6) | 2%  (1) | NA | NA | NA | NA | 100%  (48) | NA | 100%  (48) | NA | NA | NA | NA |
| Soil Science and Plant Nutrition | 1.432 | 61 | United Kingdom | Taylor and Francis Ltd. | 1955-2020 | 24 | 100%  (1) | 40%  (2) | 25%  (6) | NA | NA | 24%  (6) | 0%  (0) | NA | NA | NA | 33.3%  (8) | 66.7%  (16) | NA | 100%  (24) | NA | NA | NA | NA |
| Stochastic Environmental Research and Risk Assessment | 2.351 | 67 | United States | Springer New York | 1999-2020 | 41 | 0%  (0) | 0%  (0) | 0%  (0) | 13.2%  (5) | NA | 12%  (5) | 5%  (2) | NA | 26.8%  (11) | NA | 4.9%  (2) | 68.3%  (28) | NA | 82.9%  (34) | 14.6%  (6) | 2.4%  (1) | NA | NA |
| Sustainability | 2.576 | 85 | Switzerland | MDPI AG | 2009-2020 | 12 | 0%  (0) | 8.3%  (1) | 8.3%  (1) | NA | NA | 8%  (1) | 0%  (0) | NA | 8.3%  (1) | NA | NA | 91.7%  (11) | NA | 100%  (12) | NA | NA | NA | NA |
| Sustainability Accounting Management and Policy Journal | 2.056 | 29 | United Kingdom | Emarald Group Publishing Ltd | 2010-2020 | 96 | 100%  (1) | 37.5%  (3) | 40.9%  (9) | 29.7%  (22) | NA | 32%  (31) | 0%  (0) | 1%  (1) | 10.4%  (10) | NA | NA | 88.5%  (85) | NA | 91.7%  (88) | 8.3%  (8) | NA | NA | NA |
| Sustainability Science | 5.301 | 54 | Japan | Springer Japan | 2006-2020 | 129 | 0%  (0) | 0%  (0) | 27.2%  (28) | 15.4%  (4) | NA | 24%  (32) | 0%  (0) | 7.8%  (10) | 31%  (40) | 0.8%  (1) | NA | 60.5%  (78) | NA | 87.6%  (113) | 5.4%  (7) | 6.2%  (8) | 0.8%  (1) | NA |
| Sustainable Chemistry and Pharmacy | 3.294 | 19 | Netherlands | Elsevier BV | 2015-2020 | 39 | 0%  (0) | 0%  (0) | 25%  (1) | 20%  (7) | NA | 20%  (8) | 0%  (0) | 5.1%  (2) | 15.4%  (6) | NA | 5.1%  (2) | 74.4%  (29) | NA | 82.1%  (32) | 10.3%  (4) | 7.7%  (3) | NA | NA |
| Sustainable Production and Consumption | 3.66 | 26 | Netherlands | Elsevier BV | 2015-2021 | 27 | 100%  (1) | 100%  (1) | 25.9%  (7) | NA | NA | 26%  (7) | 0%  (0) | NA | 40.7%  (11) | 3.7%  (1) | NA | 55.6%  (15) | NA | 66.7%  (18) | 29.6%  (8) | 3.7%  (1) | NA | NA |
| Tourism Management | 7.432 | 199 | United Kingdom | Elsevier Ltd. | 1982-2021 | 87 | 100%  (1) | 50%  (2) | 26.4%  (23) | NA | NA | 26%  (23) | 0%  (0) | NA | 24.1%  (21) | NA | 1.1%  (1) | 74.7%  (65) | NA | 93.1%  (81) | 6.9%  (6) | NA | NA | NA |
| Toxicological and Environmental Chemistry | 1.05 | 38 | United Kingdom | Taylor and Francis Ltd. | 1979-2020 | 130 | 0%  (0) | 18.4%  (9) | 33.9%  (40) | 0%  (0) | NA | 32%  (40) | 5%  (7) | 3.1%  (4) | 9.2%  (12) | NA | 3.1%  (4) | 84.6%  (110) | NA | 86.2%  (112) | 12.3%  (16) | 0.8%  (1) | 0.8%  (1) | NA |
| Toxics | 3.271 | 25 | Switzerland | MDPI AG | 2013-2020 | 158 | 0%  (0) | 0%  (0) | 22.6%  (35) | NA | NA | 22%  (35) | 2%  (3) | NA | 15.2%  (24) | 1.3%  (2) | NA | 83.5%  (132) | NA | 91.8%  (145) | 8.2%  (13) | NA | NA | NA |
| Transnational Environmental Law | 2.641 | 18 | United Kingdom | Cambridge University Press | 2012-2020 | 31 | 50%  (1) | 50%  (1) | 66.7%  (4) | 40%  (10) | NA | 46%  (14) | 0%  (0) | 3.2%  (1) | 9.7%  (3) | NA | NA | 87.1%  (27) | NA | 90.3%  (28) | 6.5%  (2) | 3.2%  (1) | NA | NA |
| Transportation Research Part D-Transport and Environment | 4.577 | 99 | United Kingdom | Elsevier Ltd. | 1996-2020 | 74 | 0%  (0) | 18.8%  (3) | 17.6%  (12) | NA | NA | 18%  (12) | 8%  (6) | NA | 18.9%  (14) | NA | NA | 81.1%  (60) | NA | 85.1%  (63) | 14.9%  (11) | NA | NA | NA |
| Trends In Environmental Analytical Chemistry | 7.059 | 25 | Netherlands | Elsevier BV | 2014-2020 | 6 | 100%  (1) | 100%  (1) | 66.7%  (4) | NA | NA | 66%  (4) | 0%  (0) | NA | 33.3%  (2) | NA | 16.7%  (1) | 50%  (3) | NA | 66.7%  (4) | 33.3%  (2) | NA | NA | NA |
| Urban Climate | 3.834 | 43 | Netherlands | Elsevier BV | 2012-2020 | 47 | 0%  (0) | 27.3%  (3) | 23.4%  (11) | NA | NA | 24%  (11) | 0%  (0) | NA | 21.3%  (10) | 4.3%  (2) | 6.4%  (3) | 68.1%  (32) | NA | 78.7%  (37) | 19.1%  (9) | 2.1%  (1) | NA | NA |
| Urban Forestry & Urban Greening | 4.021 | 74 | Germany | Urban und Fischer Verlag GmbH und Co. KG | 2002-2020 | 54 | 100%  (1) | 38.9%  (7) | 35.2%  (19) | NA | NA | 36%  (19) | 0%  (0) | 1.9%  (1) | 7.4%  (4) | 3.7%  (2) | 3.7%  (2) | 83.3%  (45) | NA | 90.7%  (49) | 7.4%  (4) | 1.9%  (1) | NA | NA |
| Urban Policy and Research | 2 | 40 | United Kingdom | Routledge | 1982-2020 | 26 | 0%  (0) | 0%  (0) | 83.3%  (5) | 35%  (7) | NA | 46%  (12) | 0%  (0) | NA | 7.7%  (2) | NA | NA | 92.3%  (24) | NA | 96.2%  (25) | 3.8%  (1) | NA | NA | NA |
| Urban Studies | 2.828 | 147 | United Kingdom | SAGE Publications Ltd | 1964-2020 | 27 | 0%  (0) | 0%  (0) | 51.9%  (14) | NA | NA | 52%  (14) | 0%  (0) | 7.4%  (2) | 25.9%  (7) | NA | NA | 66.7%  (18) | NA | 77.8%  (21) | 11.1%  (3) | 11.1%  (3) | NA | NA |
| Utilities Policy | 1.835 | 51 | United Kingdom | Elsevier BV | 1990-1995, 1997-2001, 2003-2020 | 14 | 100%  (1) | 100%  (1) | 28.6%  (4) | NA | NA | 28%  (4) | 0%  (0) | NA | 7.1%  (1) | NA | NA | 92.9%  (13) | NA | 100%  (14) | NA | NA | NA | NA |
| Vadose Zone Journal | 2.504 | 81 | United States | Soil Science Society of America | 2002-2020 | 67 | 0%  (0) | 20%  (1) | 19.4%  (12) | NA | NA | 20%  (12) | 7%  (5) | NA | 14.9%  (10) | 1.5%  (1) | 1.5%  (1) | 80.6%  (54) | 1.5%  (1) | 83.6%  (56) | 14.9%  (10) | NA | NA | 1.5%  (1) |
| Waste and Biomass Valorization | 2.851 | 41 | Netherlands | Springer Netherlands | 2010-2020 | 43 | 0%  (0) | 0%  (0) | 0%  (0) | 17.6%  (6) | NA | 14%  (6) | 5%  (2) | NA | 18.6%  (8) | 2.3%  (1) | NA | 79.1%  (34) | NA | 86%  (37) | 11.6%  (5) | 2.3%  (1) | NA | NA |
| Waste Management | 5.448 | 161 | United Kingdom | Elsevier Ltd. | 1983-2020 | 48 | 0%  (0) | 33.3%  (9) | 30.4%  (14) | NA | NA | 30%  (14) | 4%  (2) | 2.1%  (1) | 29.2%  (14) | NA | NA | 68.8%  (33) | NA | 77.1%  (37) | 20.8%  (10) | NA | 2.1%  (1) | NA |
| Waste Management & Research | 2.771 | 80 | United Kingdom | SAGE Publications Ltd | 1983-2020 | 31 | 0%  (0) | 0%  (0) | 7.7%  (2) | 0%  (0) | NA | 6%  (2) | 3%  (1) | 3.2%  (1) | 12.9%  (4) | 6.5%  (2) | 12.9%  (4) | 64.5%  (20) | NA | 71%  (22) | 25.8%  (8) | NA | 3.2%  (1) | NA |
| Water Air and Soil Pollution | 1.9 | 111 | Netherlands | Springer Netherlands | 1971-2020 | 36 | 0%  (0) | 40%  (2) | 25%  (8) | NA | NA | 24%  (8) | 11%  (4) | NA | 11.1%  (4) | 2.8%  (1) | 2.8%  (1) | 83.3%  (30) | NA | 86.1%  (31) | 13.9%  (5) | NA | NA | NA |
| Water Alternatives-An Interdisciplinary Journal On Water Politics and Development | 1.979 | 42 | France | NA | 2008-2020 | 33 | 0%  (0) | 11.1%  (1) | 18.2%  (6) | NA | NA | 18%  (6) | 0%  (0) | 6.1%  (2) | 18.2%  (6) | NA | 3%  (1) | 72.7%  (24) | NA | 78.8%  (26) | 9.1%  (3) | 12.1%  (4) | NA | NA |
| Water and Environment Journal | 1.426 | 37 | United States | Wiley-Blackwell | 1987-2020 | 22 | 100%  (1) | 50%  (2) | 16.7%  (3) | NA | NA | 16%  (3) | 18%  (4) | NA | 18.2%  (4) | NA | NA | 81.8%  (18) | NA | 90.9%  (20) | 9.1%  (2) | NA | NA | NA |
| Water Environment Research | 1.369 | 73 | United States | Water Environment Federation | 1992-2020 | 41 | 0%  (0) | 30.8%  (4) | 22%  (9) | NA | NA | 22%  (9) | 0%  (0) | NA | 19.5%  (8) | NA | NA | 80.5%  (33) | NA | 85.4%  (35) | 12.2%  (5) | 2.4%  (1) | NA | NA |
| Water Research | 9.13 | 303 | United Kingdom | Elsevier Ltd. | 1967-2020 | 54 | 0%  (0) | 23.5%  (4) | 20.8%  (11) | NA | NA | 20%  (11) | 2%  (1) | NA | 37%  (20) | NA | NA | 63%  (34) | NA | 90.7%  (49) | 7.4%  (4) | 1.9%  (1) | NA | NA |
| Water Resources and Economics | 1.875 | 19 | Netherlands | Elsevier BV | 2013-2020 | 12 | 0%  (0) | 0%  (0) | 37.5%  (3) | 0%  (0) | NA | 24%  (3) | 0%  (0) | NA | NA | NA | NA | 100%  (12) | NA | 100%  (12) | NA | NA | NA | NA |
| Water Resources Research | 4.309 | 217 | United States | Wiley-Blackwell | 1965-2020 | 145 | 100%  (1) | 27.3%  (3) | 22.4%  (32) | NA | NA | 22%  (32) | 1%  (2) | NA | 4.8%  (7) | NA | NA | 95.2%  (138) | NA | 96.6%  (140) | 3.4%  (5) | NA | NA | NA |
| Water Science and Technology | 1.638 | 137 | United Kingdom | IWA Publishing | 1970, 1980-2020 | 83 | 0%  (0) | 17.6%  (3) | 19.8%  (16) | NA | NA | 20%  (16) | 2%  (2) | 3.6%  (3) | 25.3%  (21) | 4.8%  (4) | 9.6%  (8) | 56.6%  (47) | NA | 66.3%  (55) | 28.9%  (24) | 4.8%  (4) | NA | NA |
| Water Science and Technology-Water Supply | 0.9 | 39 | United Kingdom | IWA Publishing | 2001-2020 | 68 | 0%  (0) | 0%  (0) | 21.2%  (14) | NA | NA | 22%  (14) | 3%  (2) | NA | 23.5%  (16) | 1.5%  (1) | 5.9%  (4) | 69.1%  (47) | NA | 79.4%  (54) | 19.1%  (13) | 1.5%  (1) | NA | NA |
| Weather Climate and Society | 1.958 | 35 | United States | American Meteorological Society | 2009-2020 | 30 | 0%  (0) | 50%  (3) | 43.3%  (13) | NA | NA | 44%  (13) | 0%  (0) | 3.3%  (1) | 10%  (3) | 3.3%  (1) | 3.3%  (1) | 80%  (24) | NA | 90%  (27) | 10%  (3) | NA | NA | NA |
| Web Ecology | 1.56 | 17 | Germany | Copernicus GmbH | 2000-2003, 2005-2020 | 17 | 0%  (0) | 0%  (0) | 23.1%  (3) | 25%  (1) | NA | 24%  (4) | 0%  (0) | NA | NA | NA | 5.9%  (1) | 94.1%  (16) | NA | 100%  (17) | NA | NA | NA | NA |
| Wetlands | 1.783 | 87 | Netherlands | Springer Netherlands | 1981-2020 | 57 | 0%  (0) | 0%  (0) | 28.3%  (15) | NA | NA | 28%  (15) | 7%  (4) | 1.8%  (1) | 22.8%  (13) | 5.3%  (3) | 1.8%  (1) | 68.4%  (39) | NA | 78.9%  (45) | 21.1%  (12) | NA | NA | NA |
| Wetlands Ecology and Management | 1.221 | 62 | Netherlands | Springer Netherlands | 1982, 1989-2020 | 33 | 0%  (0) | 0%  (0) | 25%  (8) | NA | NA | 24%  (8) | 3%  (1) | 6.1%  (2) | 15.2%  (5) | 6.1%  (2) | 3%  (1) | 69.7%  (23) | NA | 81.8%  (27) | 15.2%  (5) | 3%  (1) | NA | NA |
| Wiley Interdisciplinary Reviews-Climate Change | 6.099 | 75 | United States | John Wiley and Sons Inc. | 2010-2020 | 29 | 0%  (0) | 0%  (0) | 46.7%  (7) | 42.9%  (6) | NA | 44%  (13) | 0%  (0) | NA | 10.3%  (3) | NA | 3.4%  (1) | 86.2%  (25) | NA | 86.2%  (25) | 6.9%  (2) | 6.9%  (2) | NA | NA |
| Wiley Interdisciplinary Reviews-Water | 4.412 | 24 | United States | John Wiley and Sons Inc. | 2014, 2017-2018, 2020 | 47 | 0%  (0) | 0%  (0) | 50%  (9) | 31%  (9) | NA | 38%  (18) | 0%  (0) | NA | 2.1%  (1) | 2.1%  (1) | 2.1%  (1) | 93.6%  (44) | NA | 95.7%  (45) | 4.3%  (2) | NA | NA | NA |

**IF:** impact factor, **EiC**: editors-in-chief, **EL**: editorial leadership, **EB**: editorial board, **AB**: advisory board, **EC:** early career/young researchers, **Unkn:** unknown; **AF:** Africa, **AP:** Asia and Pacific,  **EE:** Eastern Europe, **LAC:** Latin America and the Caribbean, **WEO:** Western Europe and Other, **HIC:** high-income countries, **UMIC:** upper-middle-income countries, **LMIC:** lower-middle-income countries, **LIC:** low income countries
