# Supplementary material for: Challenging the “old boys club” in academia: Gender and geographic representation in editorial boards of journals publishing in environmental sciences and public health
Source: PLOS Glob Public Health. 2022 Jun 21;2(6):e0000541. doi: 10.1371/journal.pgph.0000541 (PMC10021803; doi:10.1371/journal.pgph.0000541)
Supplement: S5 Table — (DOCX) [file pgph.0000541.s006.docx]

## Supplement Table 5: Characteristics of journals categorised as Public, Environmental and Occupational Health AND Environmental Sciences and Environmental Studies following the JCR

| **Journal Title** | **IF** | **H** | **Country** | **Publisher** | **Coverage** | **Total** | **%**  **(N) Inferred women and gender minority** | | | | | | **%**  **(N) UN region of editors’ institutions** | | | | | | **%**  **(N) Income group of editors' institution** | | | | | | |
| --- | --- | --- | --- | --- | --- | --- | --- | --- | --- | --- | --- | --- | --- | --- | --- | --- | --- | --- | --- | --- | --- | --- | --- | --- | --- |
|  |  |  |  |  |  |  | **EiC** | **EL** | **EB** | **AB** | **EC** | **Tot** | **Unkn** | **AF** | **AP** | **EE** | **LAC** | **WEO** | **Unkn** | **HIC** | **UMIC** | **LMIC** | **LIC** | **Unkn** |  |
| Annals of Agricultural and Environmental Medicine | 0.982 | 54 | Poland | Institute of Agricultural Medicine | 1995-2020 | 9 | 0%  (0) | 16.7%  (1) | 22.2%  (2) | NA | NA | 22%  (2) | 0%  (0) | NA | NA | 100%  (9) | NA | NA | NA | 100%  (9) | NA | NA | NA | NA |  |
| Archives of Environmental & Occupational Health | 1.18 | 62 | United States | Heldref Publications | 2005-2020 | 116 | 100%  (2) | 53.6%  (15) | 36.5%  (42) | NA | NA | 36%  (42) | 1%  (1) | 1.7%  (2) | 10.3%  (12) | 1.7%  (2) | 3.4%  (4) | 82.8%  (96) | NA | 87.1%  (101) | 11.2%  (13) | 1.7%  (2) | NA | NA |  |
| Biomedical and Environmental Sciences | 2.656 | 54 | United Kingdom | Elsevier | 1988-2020 | 49 | 100%  (1) | 100%  (1) | 13.6%  (6) | NA | NA | 14%  (6) | 10%  (5) | 2%  (1) | 49%  (24) | 6.1%  (3) | 2%  (1) | 40.8%  (20) | NA | 49%  (24) | 49%  (24) | 2%  (1) | NA | NA |  |
| Current Pollution Reports | 3.286 | 23 | Switzerland | Springer International Publishing AG | 2015-2020 | 43 | NA | 28.6%  (4) | 11.6%  (5) | NA | NA | 12%  (5) | 0%  (0) | NA | 39.5%  (17) | NA | 4.7%  (2) | 55.8%  (24) | NA | 65.1%  (28) | 16.3%  (7) | 18.6%  (8) | NA | NA |  |
| Disaster Prevention and Management | 1 | 53 | United Kingdom | Emerald Group Publishing Ltd. | 1992-2020 | 23 | 0%  (0) | 0%  (0) | 0%  (0) | 28.6%  (6) | NA | 26%  (6) | 0%  (0) | 8.7%  (2) | 21.7%  (5) | NA | 17.4%  (4) | 52.2%  (12) | NA | 60.9%  (14) | 21.7%  (5) | 17.4%  (4) | NA | NA |  |
| Environmental Geochemistry and Health | 3.472 | 71 | Netherlands | Springer Netherlands | 1983-2020 | 77 | 0%  (0) | 0%  (0) | 24.2%  (16) | 0%  (0) | NA | 22%  (16) | 8%  (6) | NA | 36.4%  (28) | 2.6%  (2) | NA | 61%  (47) | NA | 76.6%  (59) | 18.2%  (14) | 5.2%  (4) | NA | NA |  |
| Environmental Health | 4.69 | 90 | United Kingdom | BioMed Central Ltd. | 2002-2020 | 51 | 0%  (0) | 0%  (0) | 31.9%  (15) | 50%  (2) | NA | 34%  (17) | 0%  (0) | 2%  (1) | 7.8%  (4) | NA | NA | 90.2%  (46) | NA | 96.1%  (49) | 3.9%  (2) | NA | NA | NA |  |
| Environmental Health Perspectives | 8.341 | 282 | United States | Public Health Services, US Dept of Health and Human Services | 1972-2020 | 163 | 0%  (0) | 54.7%  (29) | 53.4%  (87) | NA | NA | 54%  (87) | 0%  (0) | 0.6%  (1) | 4.3%  (7) | NA | 1.8%  (3) | 93.3%  (152) | NA | 95.1%  (155) | 3.7%  (6) | 1.2%  (2) | NA | NA |  |
| Environmental Research | 5.715 | 136 | United States | Academic Press Inc. | 1967-2020 | 90 | 33.3%  (1) | 35.7%  (5) | 30.7%  (27) | NA | NA | 30%  (27) | 2%  (2) | NA | 36.7%  (33) | 1.1%  (1) | 3.3%  (3) | 58.9%  (53) | NA | 66.7%  (60) | 31.1%  (28) | 2.2%  (2) | NA | NA |  |
| Geohealth | 3.66 | 12 | United States | John Wiley and Sons Inc. | 2017-2020 | 15 | 0%  (0) | 14.3%  (1) | 26.7%  (4) | NA | NA | 26%  (4) | 0%  (0) | NA | 6.7%  (1) | NA | NA | 93.3%  (14) | NA | 93.3%  (14) | 6.7%  (1) | NA | NA | NA |  |
| Health Physics | 0.853 | 73 | United States | Lippincott Williams and Wilkins Ltd. | 1958-2020 | 67 | 0%  (0) | 0%  (0) | 10.4%  (7) | NA | NA | 10%  (7) | 0%  (0) | NA | 3%  (2) | 1.5%  (1) | NA | 95.5%  (64) | NA | 97%  (65) | 3%  (2) | NA | NA | NA |  |
| Industrial Health | 1.471 | 59 | Japan | National Institute of Industrial Health | 1963-2020 | 57 | 0%  (0) | 0%  (0) | 19.4%  (7) | 5%  (1) | NA | 14%  (8) | 2%  (1) | NA | 75.4%  (43) | NA | NA | 24.6%  (14) | NA | 98.2%  (56) | 1.8%  (1) | NA | NA | NA |  |
| International Journal of Environmental Health Research | 1.916 | 49 | United Kingdom | Taylor and Francis Ltd. | 1991-2020 | 28 | NA  (0) | NA  (0) | 0%  (0) | 26.1%  (6) | NA | 24%  (6) | 11%  (3) | NA | 25%  (7) | 7.1%  (2) | 17.9%  (5) | 50%  (14) | NA | 60.7%  (17) | 28.6%  (8) | 10.7%  (3) | NA | NA |  |
| International Journal of Environmental Research and Public Health | 2.849 | 113 | Switzerland | MDPI Multidisciplinary Digital Publishing Institute | 2004-2020 | 22 | 0%  (0) | 31.6%  (6) | 31.6%  (6) | 33.3%  (1) | NA | 32%  (7) | 0%  (0) | NA | 9.1%  (2) | NA | NA | 90.9%  (20) | NA | 100%  (22) | NA | NA | NA | NA |  |
| Journal of Environmental Science and Health, Part B Pesticides, Food Contaminants, and Agricultural Wastes | 1.697 | 50 | United States | Taylor and Francis Ltd. | 1976-2020 | 16 | 0%  (0) | 0%  (0) | 31.2%  (5) | NA | NA | 32%  (5) | 0%  (0) | 6.2%  (1) | 12.5%  (2) | 12.5%  (2) | 6.2%  (1) | 62.5%  (10) | NA | 68.8%  (11) | 18.8%  (3) | 12.5%  (2) | NA | NA |  |
| Journal of Exposure Science and Environmental Epidemiology | 3.531 | 92 | United Kingdom | Nature Publishing Group | 2006-2020 | 17 | 100%  (1) | 50%  (1) | 58.8%  (10) | NA | NA | 58%  (10) | 0%  (0) | NA | 5.9%  (1) | NA | NA | 94.1%  (16) | NA | 100%  (17) | NA | NA | NA | NA |  |
| Journal of Health Population and Nutrition | 1.821 | 61 | United Kingdom | BioMed Central Ltd. | 2000-2020 | 36 | 0%  (0) | 36.8%  (7) | 36.8%  (7) | 23.5%  (4) | NA | 30%  (11) | 0%  (0) | 5.6%  (2) | 41.7%  (15) | NA | 2.8%  (1) | 50%  (18) | NA | 58.3%  (21) | 2.8%  (1) | 33.3%  (12) | 5.6%  (2) | NA |  |
| Journal of Occupational and Environmental Hygiene | 1.653 | 57 | United Kingdom | Taylor and Francis Ltd. | 2004-2020 | 43 | 100%  (1) | 50%  (1) | 25.6%  (11) | NA | NA | 26%  (11) | 0%  (0) | NA | NA | NA | NA | 100%  (43) | NA | 100%  (43) | NA | NA | NA | NA |  |
| Journal of Radiological Protection | 1.261 | 46 | United Kingdom | IOP Publishing Ltd. | 1988-2020 | 19 | 0%  (0) | 0%  (0) | 23.5%  (4) | NA | NA | 24%  (4) | 11%  (2) | NA | NA | NA | NA | 100%  (19) | NA | 100%  (19) | NA | NA | NA | NA |  |
| Journal of Toxicology and Environmental Health-Part A-Current Issues | 2.653 | 89 | United Kingdom | Taylor and Francis Ltd. | 1996-2020 | 48 | 0%  (0) | 0%  (0) | 39.6%  (19) | NA | NA | 40%  (19) | 0%  (0) | NA | 8.3%  (4) | NA | 12.5%  (6) | 79.2%  (38) | NA | 81.2%  (39) | 18.8%  (9) | NA | NA | NA |  |
| Journal of Toxicology and Environmental Health-Part B-Critical Reviews | 6.105 | 83 | United Kingdom | Taylor and Francis Ltd. | 1998-2020 | 23 | 0%  (0) | 16.7%  (1) | 30.4%  (7) | NA | NA | 30%  (7) | 0%  (0) | NA | 8.7%  (2) | NA | 13%  (3) | 78.3%  (18) | NA | 78.3%  (18) | 21.7%  (5) | NA | NA | NA |  |
| Radiation Protection Dosimetry | 0.773 | 72 | United Kingdom | Oxford University Press | 1981-2020 | 8 | 0%  (0) | 0%  (0) | 14.3%  (1) | NA | NA | 14%  (1) | 12%  (1) | NA | NA | NA | NA | 100%  (8) | NA | 100%  (8) | NA | NA | NA | NA |  |
| Radioprotection | 0.541 | 17 | France | EDP Sciences | 1977-1981, 1988-2020 | 38 | 0%  (0) | 60%  (3) | 33.3%  (11) | 0%  (0) | NA | 32%  (11) | 8%  (3) | 2.6%  (1) | 5.3%  (2) | 7.9%  (3) | 5.3%  (2) | 78.9%  (30) | NA | 86.8%  (33) | 7.9%  (3) | 5.3%  (2) | NA | NA |  |
| Reviews on Environmental Health | 2.429 | 54 | Germany | Walter de Gruyter GmbH | 1974-1975, 1977, 1979-1982, 1984-1987, 1989, 1991, 1994, 1996-2020 | 15 | 0%  (0) | 25%  (1) | 13.3%  (2) | NA | NA | 14%  (2) | 0%  (0) | NA | 13.3%  (2) | NA | NA | 86.7%  (13) | NA | 86.7%  (13) | 6.7%  (1) | 6.7%  (1) | NA | NA |  |

**IF:** impact factor, **EiC**: editors-in-chief, **EL**: editorial leadership, **EB**: editorial board, **AB**: advisory board, **EC:** early career/young researchers, **Unkn:** unknown; **AF:** Africa, **AP:** Asia and Pacific,  **EE:** Eastern Europe, **LAC:** Latin America and the Caribbean, **WEO:** Western Europe and Other, **HIC:** high-income countries, **UMIC:** upper-middle-income countries, **LMIC:** lower-middle-income countries, **LIC:** low income countries
