# Supplementary material for: Challenging the “old boys club” in academia: Gender and geographic representation in editorial boards of journals publishing in environmental sciences and public health
Source: PLOS Glob Public Health. 2022 Jun 21;2(6):e0000541. doi: 10.1371/journal.pgph.0000541 (PMC10021803; doi:10.1371/journal.pgph.0000541)
Supplement: S6 Table — (DOCX) [file pgph.0000541.s007.docx]

## Supplement Table 6: Inferred gender composition of editorial boards by World Bank income group and United Nations (UN) geographic region of editors’ institutions.

|  | **%**  **(N) Inferred women and gender minority** | | | | | | | | | | | |
| --- | --- | --- | --- | --- | --- | --- | --- | --- | --- | --- | --- | --- |
|  | **EiC** | | **EL** | | **EB** | | **AB** | | **EC** | | **Total** | |
| **World Bank National Income Group** | M | WGM | M | WGM | M | WGM | M | WGM | M | WGM | M | WGM |
| *High-income* | 74%  (540) | 26%  (181) | 64%  (2480) | 36%  (1426) | 66%  (13077) | 34%  (6954) | 68%  (2114) | 32%  (971) | 38%  (31) | 62%  (50) | 66%  (15191) | 34%  (7925) |
| *Upper-middle income* | 72%  (46) | 28%  (18) | 70%  (385) | 30%  (167) | 70%  (1953) | 30%  (825) | 72%  (275) | 28%  (107) | 74%  (19) | 26%  (7) | 70%  (2228) | 30%  (932) |
| *Lower-middle income* | 84%  (11) | 16%  (2) | 72%  (110) | 28%  (42) | 76%  (657) | 24%  (213) | 76%  (116) | 24%  (37) | 66%  (2) | 34%  (1) | 76%  (773) | 24%  (250) |
| *Low income* | 100%  (1) | 0%  (0) | 84%  (10) | 16%  (2) | 76%  (82) | 24%  (26) | 90%  (9) | 10%  (1) | NA | NA | 78%  (91) | 22%  (27) |
|  |  |  |  |  |  |  |  |  |  |  |  |  |
|  | **EiC** | | **EL** | | **EB** | | **AB** | | **EC** | | **Total** | |
| **United Nations Geographic Region** | M | WGM | M | WGM | M | WGM | M | WGM | M | WGM | M | WGM |
| *Africa* | 70%  (7) | 30%  (3) | 60%  (53) | 40%  (35) | 64%  (404) | 36%  (229) | 74%  (84) | 26%  (30) | 100%  (1) | 0%  (0) | 66%  (488) | 34%  (259) |
| *Asia and the Pacific* | 82%  (63) | 18%  (14) | 80%  (555) | 20%  (137) | 80%  (2656) | 20%  (660) | 78%  (348) | 22%  (100) | 72%  (20) | 28%  (8) | 80%  (3004) | 20%  (760) |
| *Eastern European* | 54%  (13) | 46%  (11) | 56%  (63) | 44%  (48) | 62%  (398) | 38%  (250) | 72%  (48) | 28%  (19) | 34%  (1) | 66%  (2) | 62%  (446) | 38%  (269) |
| *Latin American and Caribbean* | 52%  (12) | 48%  (11) | 58%  (115) | 42%  (82) | 62%  (545) | 38%  (340) | 62%  (72) | 38%  (44) | 100%  (1) | 0%  (0) | 62%  (617) | 38%  (384) |
| *Western European and Others* | 76%  (503) | 24%  (162) | 62%  (2199) | 38%  (1336) | 64%  (11771) | 36%  (6542) | 68%  (1963) | 32%  (924) | 38%  (29) | 62%  (48) | 64%  (13734) | 36%  (7466) |

**EiC**: editor-in-chiefs, **EL**: editorial leadership, **EB**: editorial board, **AB**: advisory board, **EC:** early career/young researchers, **M:** men, **WGM:** women and gender minorities
