# Supplementary material for: Challenging the “old boys club” in academia: Gender and geographic representation in editorial boards of journals publishing in environmental sciences and public health
Source: PLOS Glob Public Health. 2022 Jun 21;2(6):e0000541. doi: 10.1371/journal.pgph.0000541 (PMC10021803; doi:10.1371/journal.pgph.0000541)
Supplement: S7 Table — (DOCX) [file pgph.0000541.s008.docx]

## Supplement Table 7: Inferred women and gender minority composition of editorial boards categorised by UN region and World Bank income group of editors’ institutions

| **UN Region** | **Income group** | **Editors’ institution country** | **GII** | **# Journals** | **Total # of editors** | **%**  **(N) Inferred women and gender minority** | | | | | | |
| --- | --- | --- | --- | --- | --- | --- | --- | --- | --- | --- | --- | --- |
|  |  |  |  |  |  | **EiC** | **EL** | **EB** | **AB** | **EC** | **Tot** | **Unknown** |
| **Africa** | *High-income* | Senegal | 0.533 | 7 | 8 | NA | 0%  (0) | 24%  (1) | 0%  (0) | NA | 12%  (1) | 0%  (0) |
|  |  | South Africa | 0.406 | 163 | 339 | 34%  (2) | 46%  (21) | 44%  (131) | 40%  (18) | 0%  (0) | 44%  (149) | 1%  (2) |
|  |  | Tunisia | 0.296 | 5 | 5 | NA | NA | 24%  (1) | 0%  (0) | NA | 20%  (1) | 0%  (0) |
|  | *Upper-middle-income* | Botswana | 0.465 | 10 | 11 | 100%  (1) | 100%  (2) | 62%  (5) | 0%  (0) | NA | 46%  (5) | 0%  (0) |
|  |  | Libya | 0.252 | 1 | 1 | NA | NA | 0%  (0) | NA | NA | 0%  (0) | 0%  (0) |
|  |  | Mauritius | 0.347 | 3 | 4 | NA | NA | 100%  (2) | 0%  (0) | NA | 50%  (2) | 0%  (0) |
|  |  | Namibia | 0.440 | 5 | 6 | NA | NA | 66%  (4) | NA | NA | 66%  (4) | 0%  (0) |
|  |  | Sierra Leone | 0.644 | 4 | 4 | NA | NA | 34%  (1) | 0%  (0) | NA | 24%  (1) | 0%  (0) |
|  | *Lower-middle-income* | Algeria | 0.429 | 2 | 3 | NA | NA | 0%  (0) | 0%  (0) | NA | 0%  (0) | 0%  (0) |
|  |  | Angola | 0.536 | 1 | 1 | NA | NA | NA | 0%  (0) | NA | 0%  (0) | 0%  (0) |
|  |  | Benin | 0.612 | 3 | 3 | NA | 0%  (0) | 0%  (0) | NA | NA | 0%  (0) | 0%  (0) |
|  |  | Cameroon | 0.560 | 4 | 5 | NA | NA | 24%  (1) | 0%  (0) | NA | 20%  (1) | 0%  (0) |
|  |  | Côte d'Ivoire | 0.638 | 2 | 2 | NA | 0%  (0) | 0%  (0) | NA | NA | 0%  (0) | 0%  (0) |
|  |  | Egypt | 0.449 | 27 | 41 | 0%  (0) | 42%  (3) | 38%  (14) | 24%  (1) | NA | 36%  (15) | 0%  (0) |
|  |  | Eswatini | .. | 2 | 2 | NA | NA | 50%  (1) | NA | NA | 50%  (1) | 0%  (0) |
|  |  | Ghana | 0.538 | 25 | 43 | NA | 0%  (0) | 16%  (5) | 30%  (3) | NA | 20%  (8) | 2%  (1) |
|  |  | Kenya | 0.518 | 42 | 56 | NA | 50%  (3) | 44%  (19) | 30%  (4) | NA | 42%  (23) | 0%  (0) |
|  |  | Lesotho | 0.553 | 1 | 1 | NA | NA | 0%  (0) | NA | NA | 0%  (0) | 0%  (0) |
|  |  | Morocco | 0.454 | 7 | 7 | NA | 50%  (1) | 34%  (2) | 0%  (0) | NA | 28%  (2) | 0%  (0) |
|  |  | Nigeria | .. | 41 | 62 | 0%  (0) | 34%  (1) | 20%  (9) | 22%  (3) | NA | 20%  (12) | 2%  (1) |
|  |  | Republic of Congo | 0.570 | 3 | 3 | NA | NA | 66%  (2) | NA | NA | 66%  (2) | 0%  (0) |
|  |  | Tanzania | 0.556 | 14 | 17 | NA | 100%  (2) | 28%  (4) | 0%  (0) | NA | 24%  (4) | 0%  (0) |
|  |  | Zambia | 0.539 | 7 | 7 | NA | 0%  (0) | 14%  (1) | NA | NA | 14%  (1) | 0%  (0) |
|  |  | Zimbabwe | 0.527 | 5 | 6 | NA | 0%  (0) | 20%  (1) | 0%  (0) | NA | 16%  (1) | 0%  (0) |
|  | *Low-income* | Burkina Faso | 0.594 | 4 | 7 | NA | NA | 0%  (0) | 0%  (0) | NA | 0%  (0) | 0%  (0) |
|  |  | Central African Republic | 0.680 | 1 | 1 | NA | NA | 0%  (0) | NA | NA | 0%  (0) | 0%  (0) |
|  |  | DRC | 0.617 | 1 | 1 | NA | 0%  (0) | 0%  (0) | NA | NA | 0%  (0) | 0%  (0) |
|  |  | Ethiopia | 0.517 | 15 | 36 | NA | 16%  (1) | 12%  (4) | 0%  (0) | NA | 12%  (4) | 0%  (0) |
|  |  | Gambia | 0.612 | 1 | 1 | NA | NA | 100%  (1) | NA | NA | 100%  (1) | 0%  (0) |
|  |  | Madagascar | .. | 1 | 1 | NA | NA | 100%  (1) | NA | NA | 100%  (1) | 0%  (0) |
|  |  | Malawi | 0.565 | 9 | 25 | 0%  (0) | 50%  (1) | 42%  (10) | 100%  (1) | NA | 44%  (11) | 0%  (0) |
|  |  | Mali | 0.671 | 3 | 3 | NA | NA | 0%  (0) | NA | NA | 0%  (0) | 0%  (0) |
|  |  | Mozambique | 0.523 | 2 | 2 | NA | NA | 50%  (1) | NA | NA | 50%  (1) | 0%  (0) |
|  |  | Niger | 0.642 | 2 | 2 | NA | NA | 0%  (0) | NA | NA | 0%  (0) | 0%  (0) |
|  |  | Rwanda | 0.402 | 6 | 6 | NA | 0%  (0) | 50%  (2) | 0%  (0) | NA | 40%  (2) | 17%  (1) |
|  |  | Sudan | 0.545 | 2 | 2 | NA | NA | NA | 0%  (0) | NA | 0%  (0) | 0%  (0) |
|  |  | Togo | 0.573 | 1 | 1 | NA | NA | 0%  (0) | NA | NA | 0%  (0) | 0%  (0) |
|  |  | Uganda | 0.535 | 21 | 27 | NA | 0%  (0) | 24%  (6) | 0%  (0) | NA | 22%  (6) | 0%  (0) |
| **Asia and Pacific** | *High-income* | Bahrain | 0.212 | 2 | 3 | NA | NA | 66%  (2) | NA | NA | 66%  (2) | 0%  (0) |
|  |  | Brunei Darussalam | 0.255 | 6 | 6 | NA | 0%  (0) | 0%  (0) | 0%  (0) | NA | 0%  (0) | 0%  (0) |
|  |  | Cyprus | 0.086 | 22 | 26 | 100%  (2) | 60%  (3) | 34%  (8) | 50%  (1) | 100%  (1) | 34%  (9) | 0%  (0) |
|  |  | French Polynesia | .. | 1 | 1 | NA | NA | 0%  (0) | NA | NA | 0%  (0) | 0%  (0) |
|  |  | Hong Kong | .. | 84 | 135 | 20%  (1) | 12%  (3) | 28%  (32) | 42%  (8) | 50%  (1) | 30%  (40) | 1%  (1) |
|  |  | Japan | 0.094 | 208 | 535 | 0%  (0) | 16%  (17) | 14%  (64) | 16%  (10) | NA | 14%  (74) | 1%  (7) |
|  |  | Kuwait | 0.242 | 5 | 6 | NA | 0%  (0) | 16%  (1) | NA | NA | 16%  (1) | 0%  (0) |
|  |  | Macao | .. | 1 | 2 | NA | 100%  (1) | 50%  (1) | NA | NA | 50%  (1) | 0%  (0) |
|  |  | Oman | 0.306 | 11 | 11 | NA | 0%  (0) | 0%  (0) | NA | NA | 0%  (0) | 9%  (1) |
|  |  | Qatar | 0.185 | 9 | 11 | NA | NA | 30%  (3) | NA | NA | 30%  (3) | 9%  (1) |
|  |  | Sri Lanka | 0.401 | 10 | 11 | NA | 50%  (1) | 44%  (4) | 50%  (1) | NA | 46%  (5) | 0%  (0) |
|  |  | Taiwan | .. | 84 | 123 | NA | 20%  (6) | 22%  (24) | 30%  (3) | NA | 22%  (27) | 3%  (4) |
|  |  | The Cook Islands | .. | 1 | 1 | NA | NA | 0%  (0) | NA | NA | 0%  (0) | 0%  (0) |
|  |  | United Arab Emirates | 0.079 | 18 | 21 | NA | 0%  (0) | 16%  (3) | 0%  (0) | NA | 14%  (3) | 0%  (0) |
|  | *Upper-middle-income* | China | 0.168 | 323 | 1559 | 16%  (5) | 18%  (48) | 18%  (231) | 16%  (27) | 26%  (4) | 18%  (258) | 6%  (88) |
|  |  | Fiji | 0.370 | 3 | 5 | NA | NA | 50%  (2) | NA  (0) | NA | 50%  (2) | 20%  (1) |
|  |  | Iraq | 0.577 | 1 | 1 | NA | NA | 0%  (0) | NA | NA | 0%  (0) | 0%  (0) |
|  |  | Jordan | 0.450 | 8 | 10 | NA | NA | 38%  (3) | 0%  (0) | NA | 30%  (3) | 0%  (0) |
|  |  | Kazakhstan | 0.190 | 6 | 7 | NA | 0%  (0) | 28%  (2) | NA | NA | 28%  (2) | 0%  (0) |
|  |  | Lebanon | 0.411 | 24 | 28 | NA | 66%  (2) | 52%  (12) | 80%  (4) | NA | 58%  (16) | 0%  (0) |
|  |  | Malaysia | 0.253 | 42 | 89 | 100%  (1) | 14%  (1) | 36%  (27) | 24%  (3) | 16%  (1) | 34%  (30) | 2%  (2) |
|  |  | Republic of the Marshall Islands | .. | 1 | 1 | NA | NA | NA | 100%  (1) | NA | 100%  (1) | 0%  (0) |
|  |  | South Korea | 0.064 | 120 | 261 | 10%  (1) | 22%  (16) | 18%  (38) | 6%  (2) | 0%  (0) | 16%  (40) | 6%  (16) |
|  |  | Thailand | 0.359 | 59 | 83 | 0%  (0) | 24%  (3) | 30%  (21) | 62%  (8) | NA | 36%  (29) | 1%  (1) |
|  | *Lower-middle-income* | Bangladesh | 0.537 | 15 | 31 | NA | 20%  (1) | 30%  (7) | 62%  (5) | NA | 38%  (12) | 0%  (0) |
|  |  | Cambodia | 0.474 | 1 | 1 | NA | NA | 0%  (0) | NA | NA | 0%  (0) | 0%  (0) |
|  |  | India | 0.488 | 205 | 425 | 24%  (1) | 24%  (19) | 24%  (85) | 20%  (12) | 0%  (0) | 24%  (97) | 1%  (6) |
|  |  | Indonesia | 0.480 | 22 | 24 | NA | NA | 16%  (3) | 0%  (0) | NA | 14%  (3) | 4%  (1) |
|  |  | Iran | 0.459 | 39 | 107 | 24%  (1) | 30%  (7) | 20%  (19) | 0%  (0) | NA | 18%  (19) | 3%  (3) |
|  |  | Mongolia | 0.322 | 1 | 1 | NA | NA | 0%  (0) | NA | NA | 0%  (0) | 0%  (0) |
|  |  | Myanmar | 0.478 | 1 | 2 | NA | NA | 100%  (2) | NA | NA | 100%  (2) | 0%  (0) |
|  |  | Nepal | 0.452 | 11 | 34 | 0%  (0) | 0%  (0) | 10%  (3) | 0%  (0) | NA | 8%  (3) | 0%  (0) |
|  |  | Pakistan | 0.538 | 22 | 41 | NA | 0%  (0) | 18%  (7) | 66%  (2) | NA | 22%  (9) | 0%  (0) |
|  |  | Palestine | .. | 4 | 4 | NA | NA | 24%  (1) | NA | NA | 24%  (1) | 0%  (0) |
|  |  | Papua New Guinea | 0.725 | 1 | 1 | NA | NA | NA | 0%  (0) | NA | 0%  (0) | 0%  (0) |
|  |  | Philippines | 0.430 | 29 | 44 | 0%  (0) | 50%  (3) | 46%  (15) | 66%  (6) | 100%  (1) | 50%  (21) | 5%  (2) |
|  |  | Saudi Arabia | 0.252 | 35 | 80 | 50%  (1) | 18%  (2) | 16%  (11) | 34%  (1) | NA | 16%  (12) | 5%  (4) |
|  |  | Uzbekistan | 0.288 | 2 | 3 | NA | 0%  (0) | 0%  (0) | NA | NA | 0%  (0) | 0%  (0) |
|  |  | Vietnam | 0.296 | 20 | 24 | NA | 34%  (1) | 10%  (2) | 0%  (0) | NA | 8%  (2) | 4%  (1) |
|  | *Low-income* | Singapore | 0.065 | 110 | 146 | 34%  (1) | 14%  (3) | 22%  (27) | 36%  (6) | NA | 22%  (33) | 1%  (1) |
| **Eastern European** | *High-income* | Croatia | 0.116 | 17 | 38 | 100%  (2) | 76%  (3) | 54%  (20) | 100%  (1) | NA | 56%  (21) | 0%  (0) |
|  |  | Czech Republic | 0.136 | 49 | 65 | 0%  (0) | 8%  (1) | 26%  (15) | 0%  (0) | 100%  (1) | 24%  (15) | 0%  (0) |
|  |  | Estonia | 0.086 | 18 | 18 | NA | 0%  (0) | 18%  (3) | 50%  (1) | NA | 22%  (4) | 0%  (0) |
|  |  | Hungary | 0.233 | 37 | 63 | 0%  (0) | 50%  (2) | 34%  (19) | 38%  (3) | NA | 34%  (22) | 0%  (0) |
|  |  | Latvia | 0.176 | 4 | 4 | NA | NA | 100%  (4) | NA | NA | 100%  (4) | 0%  (0) |
|  |  | Lithuania | 0.124 | 10 | 30 | 0%  (0) | 0%  (0) | 56%  (15) | 50%  (1) | NA | 56%  (16) | 3%  (1) |
|  |  | Poland | 0.115 | 81 | 287 | 50%  (8) | 50%  (30) | 38%  (100) | 30%  (7) | NA | 38%  (107) | 1%  (3) |
|  |  | Slovakia | 0.191 | 15 | 19 | 100%  (1) | 100%  (1) | 48%  (8) | 0%  (0) | NA | 42%  (8) | 0%  (0) |
|  |  | Slovenia | 0.063 | 24 | 44 | 0%  (0) | 76%  (3) | 52%  (21) | 76%  (3) | NA | 54%  (24) | 0%  (0) |
|  | *Upper-middle-income* | Albania | 0.181 | 1 | 1 | NA | NA | 100%  (1) | NA | NA | 100%  (1) | 0%  (0) |
|  |  | Armenia | 0.245 | 1 | 1 | NA | 100%  (1) | 100%  (1) | NA | NA | 100%  (1) | 0%  (0) |
|  |  | Azerbaijan | 0.323 | 2 | 2 | NA | NA | 50%  (1) | NA | NA | 50%  (1) | 0%  (0) |
|  |  | Bulgaria | 0.206 | 8 | 10 | NA | 0%  (0) | 0%  (0) | 50%  (1) | 0%  (0) | 10%  (1) | 0%  (0) |
|  |  | Georgia | 0.331 | 3 | 3 | NA | NA | 34%  (1) | NA | NA | 34%  (1) | 0%  (0) |
|  |  | North Macedonia | 0.143 | 4 | 4 | NA | NA | 66%  (2) | NA | NA | 66%  (2) | 25%  (1) |
|  |  | Romania | 0.276 | 20 | 25 | NA | 76%  (3) | 48%  (11) | 0%  (0) | 100%  (1) | 46%  (11) | 4%  (1) |
|  |  | Russia | 0.225 | 46 | 61 | NA | 50%  (2) | 26%  (14) | 12%  (1) | NA | 24%  (15) | 0%  (0) |
|  | *Lower-middle-income* | Kyrgyzstan | 0.369 | 1 | 1 | NA | NA | 0%  (0) | NA | NA | 0%  (0) | 0%  (0) |
|  |  | Serbia | 0.132 | 29 | 35 | 0%  (0) | 22%  (2) | 38%  (12) | 34%  (1) | NA | 38%  (13) | 0%  (0) |
|  |  | Ukraine | 0.234 | 13 | 13 | NA | 0%  (0) | 30%  (3) | 0%  (0) | NA | 24%  (3) | 8%  (1) |
| **Latin America and Caribbean** | *High-income* | Barbados | 0.252 | 4 | 4 | NA | NA | 24%  (1) | NA | NA | 24%  (1) | 0%  (0) |
|  |  | Bermuda | .. | 1 | 1 | NA | NA | 0%  (0) | NA | NA | 0%  (0) | 0%  (0) |
|  |  | Chile | 0.247 | 73 | 100 | 66%  (2) | 36%  (5) | 30%  (26) | 24%  (3) | NA | 30%  (29) | 1%  (1) |
|  |  | Trinidad and Tobago | 0.323 | 10 | 10 | NA | 0%  (0) | 28%  (2) | 100%  (3) | NA | 50%  (5) | 0%  (0) |
|  |  | Uruguay | 0.288 | 14 | 14 | NA | 0%  (0) | 24%  (3) | 50%  (1) | NA | 28%  (4) | 0%  (0) |
|  | *Upper-middle-income* | Argentina | 0.328 | 69 | 113 | 0%  (0) | 30%  (9) | 34%  (34) | 40%  (6) | NA | 36%  (40) | 0%  (0) |
|  |  | Brazil | 0.408 | 186 | 467 | 58%  (8) | 52%  (42) | 44%  (175) | 38%  (21) | NA | 42%  (196) | 1%  (6) |
|  |  | Colombia | 0.428 | 37 | 43 | 0%  (0) | 24%  (2) | 38%  (15) | 34%  (1) | NA | 38%  (16) | 2%  (1) |
|  |  | Costa Rica | 0.288 | 13 | 14 | NA | 0%  (0) | 34%  (3) | 0%  (0) | NA | 22%  (3) | 0%  (0) |
|  |  | Cuba | 0.304 | 6 | 14 | NA | 100%  (1) | 22%  (3) | NA | NA | 22%  (3) | 0%  (0) |
|  |  | Ecuador | 0.384 | 3 | 3 | NA | NA | 34%  (1) | NA | NA | 34%  (1) | 0%  (0) |
|  |  | Guatemala | 0.479 | 2 | 2 | NA | 0%  (0) | 50%  (1) | NA | NA | 50%  (1) | 0%  (0) |
|  |  | Jamaica | 0.396 | 10 | 11 | NA | NA | 50%  (5) | 100%  (1) | NA | 54%  (6) | 0%  (0) |
|  |  | Mexico | 0.322 | 93 | 170 | 34%  (1) | 42%  (21) | 38%  (59) | 58%  (7) | 0%  (0) | 38%  (66) | 0%  (0) |
|  |  | Panama | 0.407 | 2 | 2 | NA | NA | 50%  (1) | NA | NA | 50%  (1) | 0%  (0) |
|  |  | Peru | 0.395 | 25 | 26 | NA | 14%  (1) | 28%  (6) | 0%  (0) | NA | 24%  (6) | 0%  (0) |
|  | *Lower-middle-income* | Bolivia | 0.417 | 1 | 2 | NA | NA | 50%  (1) | NA | NA | 50%  (1) | 0%  (0) |
|  |  | El Salvador | 0.383 | 1 | 1 | NA | NA | 100%  (1) | NA | NA | 100%  (1) | 0%  (0) |
|  |  | Haiti | 0.636 | 1 | 1 | NA | NA  (0) | NA  (0) | NA | NA | NA  (0) | 100%  (1) |
|  |  | Honduras | 0.423 | 1 | 1 | NA | 0%  (0) | 0%  (0) | NA | NA | 0%  (0) | 0%  (0) |
|  |  | St. Lucia | 0.401 | 1 | 1 | NA | NA | 0%  (0) | NA | NA | 0%  (0) | 0%  (0) |
|  | *Unclassified* | Venezuela | 0.479 | 10 | 10 | NA | 100%  (1) | 38%  (3) | 50%  (1) | NA | 40%  (4) | 0%  (0) |
| **Western European and Other** | *High-income* | Australia | 0.097 | 388 | 1472 | 24%  (11) | 44%  (121) | 42%  (508) | 34%  (82) | 76%  (6) | 40%  (590) | 1%  (11) |
|  |  | Austria | 0.069 | 123 | 190 | 0%  (0) | 12%  (3) | 22%  (36) | 52%  (15) | NA | 26%  (51) | 1%  (1) |
|  |  | Belgium | 0.043 | 131 | 206 | 14%  (1) | 40%  (15) | 26%  (46) | 12%  (3) | NA | 24%  (49) | 1%  (3) |
|  |  | Canada | 0.080 | 389 | 1366 | 20%  (10) | 38%  (86) | 38%  (445) | 36%  (68) | 0%  (0) | 38%  (513) | 1%  (16) |
|  |  | Denmark | 0.038 | 146 | 271 | 0%  (0) | 24%  (13) | 30%  (69) | 32%  (14) | NA | 30%  (83) | 0%  (1) |
|  |  | Finland | 0.047 | 128 | 207 | 0%  (0) | 40%  (12) | 30%  (49) | 34%  (16) | NA | 32%  (65) | 0%  (0) |
|  |  | France | 0.049 | 239 | 530 | 20%  (4) | 34%  (36) | 32%  (148) | 28%  (18) | 100%  (1) | 32%  (166) | 1%  (6) |
|  |  | Germany | 0.084 | 302 | 985 | 14%  (4) | 14%  (22) | 24%  (189) | 20%  (35) | 100%  (2) | 22%  (224) | 1%  (5) |
|  |  | Greece | 0.116 | 93 | 157 | 0%  (0) | 32%  (10) | 26%  (36) | 62%  (8) | NA | 28%  (44) | 1%  (1) |
|  |  | Greenland | .. | 1 | 1 | NA | NA | NA | 100%  (1) | NA | 100%  (1) | 0%  (0) |
|  |  | Iceland | 0.058 | 15 | 18 | NA | 100%  (3) | 54%  (7) | 60%  (3) | NA | 56%  (10) | 0%  (0) |
|  |  | Ireland | 0.093 | 64 | 88 | 0%  (0) | 12%  (1) | 28%  (19) | 34%  (6) | 0%  (0) | 28%  (25) | 0%  (0) |
|  |  | Israel | 0.109 | 84 | 139 | 34%  (1) | 44%  (9) | 34%  (43) | 50%  (6) | NA | 36%  (49) | 0%  (0) |
|  |  | Italy | 0.069 | 252 | 844 | 34%  (5) | 42%  (73) | 34%  (246) | 24%  (26) | 0%  (0) | 32%  (272) | 0%  (3) |
|  |  | Liechtenstein | .. | 1 | 1 | NA | NA | NA | 0%  (0) | NA | 0%  (0) | 0%  (0) |
|  |  | Luxembourg | 0.065 | 11 | 11 | NA | 0%  (0) | 44%  (4) | 50%  (1) | NA | 46%  (5) | 0%  (0) |
|  |  | Malta | 0.175 | 6 | 6 | NA | 0%  (0) | 50%  (2) | 100%  (2) | NA | 66%  (4) | 0%  (0) |
|  |  | Monaco | .. | 1 | 2 | NA | 0%  (0) | 0%  (0) | NA | NA | 0%  (0) | 0%  (0) |
|  |  | Netherlands | 0.043 | 252 | 620 | 20%  (5) | 34%  (38) | 28%  (135) | 32%  (39) | 0%  (0) | 28%  (174) | 1%  (4) |
|  |  | New Zealand | 0.123 | 142 | 233 | 0%  (0) | 34%  (12) | 36%  (71) | 30%  (12) | NA | 36%  (83) | 0%  (1) |
|  |  | Norway | 0.045 | 133 | 209 | 0%  (0) | 20%  (5) | 30%  (49) | 50%  (21) | NA | 34%  (70) | 1%  (2) |
|  |  | Portugal | 0.075 | 81 | 135 | 0%  (0) | 32%  (7) | 40%  (47) | 62%  (10) | 100%  (1) | 44%  (57) | 3%  (4) |
|  |  | Spain | 0.070 | 223 | 550 | 24%  (3) | 38%  (36) | 32%  (156) | 18%  (9) | 50%  (1) | 30%  (165) | 0%  (0) |
|  |  | Sweden | 0.039 | 190 | 412 | 34%  (4) | 42%  (27) | 36%  (116) | 32%  (26) | 0%  (0) | 34%  (142) | 0%  (2) |
|  |  | Switzerland | 0.025 | 199 | 420 | 16%  (2) | 32%  (19) | 30%  (95) | 36%  (35) | 100%  (1) | 30%  (130) | 0%  (0) |
|  |  | United Kingdom | 0.118 | 449 | 2373 | 32%  (36) | 38%  (127) | 34%  (665) | 30%  (136) | 34%  (1) | 34%  (801) | 1%  (12) |
|  |  | United States | 0.204 | 566 | 9761 | 26%  (76) | 40%  (655) | 38%  (3320) | 32%  (325) | 68%  (34) | 38%  (3645) | 1%  (65) |
|  | *Upper-middle-income* | Turkey | 0.306 | 66 | 114 | 0%  (0) | 38%  (6) | 40%  (40) | 50%  (7) | 100%  (1) | 42%  (47) | 0%  (0) |

**EiC**: editors-in-chief, **EL**: editorial leadership, **EB**: editorial board, **AB**: advisory board, **EC:** early career/young researchers
