# Supplementary material for: Challenging the “old boys club” in academia: Gender and geographic representation in editorial boards of journals publishing in environmental sciences and public health
Source: PLOS Glob Public Health. 2022 Jun 21;2(6):e0000541. doi: 10.1371/journal.pgph.0000541 (PMC10021803; doi:10.1371/journal.pgph.0000541)
Supplement: S8 Table — (DOCX) [file pgph.0000541.s009.docx]

## Supplement Table 8: Representation of editors by country of journals

| **UN Region of Journal** | **Country** | **GII** | **# Journals** | **Total # of editors** | **%**  **(N) Inferred women and gender minority** | | | | | | **%**  **(N) UN region of editors’ institutions** | | | | | | **%**  **(N) Income group of editors' institution** | | | | | |
| --- | --- | --- | --- | --- | --- | --- | --- | --- | --- | --- | --- | --- | --- | --- | --- | --- | --- | --- | --- | --- | --- | --- |
|  |  |  |  |  | **EiC** | **EL** | **EB** | **AB** | **EC** | **Tot** | **Unkn** | **AF** | **AP** | **EE** | **LAC** | **WEO** | **Unkn** | **HIC** | **UMIC** | **LMIC** | **LIC** | **Unkn** |
| **Africa** | Ethiopia | 0.517 | 1 | 10 | NA | NA | 30%  (3) | NA | NA | 30%  (3) | 0%  (0) | 90%  (9) | 0%  (0) | 0%  (0) | 0%  (0) | 10%  (1) | 0%  (0) | 10%  (1) | 0%  (0) | 0%  (0) | 90%  (9) | 0%  (0) |
|  | Malawi | 0.565 | 1 | 17 | 0%  (0) | 0%  (0) | 36%  (6) | NA | NA | 36%  (6) | 0%  (0) | 100%  (17) | 0%  (0) | 0%  (0) | 0%  (0) | 0%  (0) | 0%  (0) | 0%  (0) | 0%  (0) | 0%  (0) | 100%  (17) | 0%  (0) |
|  | Nigeria | .. | 1 | 37 | 0%  (0) | 0%  (0) | 24%  (1) | 30%  (9) | NA | 28%  (10) | 5%  (2) | 54%  (20) | 0%  (0) | 0%  (0) | 0%  (0) | 46%  (17) | 0%  (0) | 46%  (17) | 16%  (6) | 38%  (14) | 0%  (0) | 0%  (0) |
| **Asia and Pacific** | China | 0.168 | 3 | 245 | 0%  (0) | 0%  (0) | 10%  (24) | 14%  (1) | 28%  (4) | 10%  (25) | 2%  (6) | 0%  (2) | 64%  (157) | 2%  (6) | 0%  (0) | 32%  (80) | 0%  (0) | 38%  (95) | 56%  (139) | 4%  (11) | 0%  (0) | 0%  (0) |
|  | India | 0.488 | 4 | 173 | 12%  (1) | 20%  (7) | 32%  (48) | 24%  (5) | NA | 30%  (53) | 1%  (1) | 6%  (10) | 36%  (61) | 6%  (10) | 4%  (8) | 48%  (84) | 0%  (0) | 56%  (97) | 18%  (32) | 26%  (44) | 0%  (0) | 0%  (0) |
|  | Iran | 0.459 | 2 | 71 | 0%  (0) | 20%  (7) | 14%  (8) | 22%  (2) | NA | 14%  (10) | 4%  (3) | 4%  (3) | 54%  (38) | 4%  (3) | 4%  (3) | 34%  (24) | 0%  (0) | 46%  (32) | 18%  (13) | 36%  (25) | 2%  (1) | 0%  (0) |
|  | Japan | 0.094 | 6 | 414 | 10%  (1) | 24%  (22) | 24%  (89) | 10%  (5) | 30%  (3) | 22%  (94) | 1%  (4) | 2%  (10) | 62%  (257) | 0%  (2) | 2%  (9) | 32%  (136) | 0%  (0) | 88%  (362) | 10%  (38) | 4%  (13) | 0%  (1) | 0%  (0) |
|  | Nepal | 0.452 | 1 | 34 | 0%  (0) | 0%  (0) | 14%  (5) | NA | NA | 14%  (5) | 0%  (0) | 0%  (0) | 56%  (19) | 0%  (0) | 0%  (0) | 44%  (15) | 0%  (0) | 44%  (15) | 0%  (0) | 56%  (19) | 0%  (0) | 0%  (0) |
|  | Philippines | 0.430 | 1 | 11 | 0%  (0) | 0%  (0) | 28%  (3) | NA | NA | 28%  (3) | 0%  (0) | 0%  (0) | 82%  (9) | 0%  (0) | 0%  (0) | 18%  (2) | 0%  (0) | 28%  (3) | 18%  (2) | 54%  (6) | 0%  (0) | 0%  (0) |
|  | Singapore | 0.065 | 1 | 11 | 0%  (0) | 0%  (0) | 0%  (0) | NA | NA | 0%  (0) | 0%  (0) | 10%  (1) | 10%  (1) | 0%  (0) | 0%  (0) | 82%  (9) | 0%  (0) | 90%  (10) | 10%  (1) | 0%  (0) | 0%  (0) | 0%  (0) |
|  | South Korea | 0.064 | 2 | 113 | 34%  (1) | 16%  (5) | 24%  (22) | 0%  (0) | NA | 20%  (22) | 2%  (2) | 0%  (1) | 56%  (64) | 0%  (0) | 2%  (3) | 40%  (45) | 0%  (0) | 90%  (102) | 8%  (8) | 2%  (3) | 0%  (0) | 0%  (0) |
|  | Thailand | 0.359 | 1 | 24 | 0%  (0) | 0%  (0) | 16%  (2) | 28%  (3) | NA | 22%  (5) | 4%  (1) | 0%  (0) | 80%  (19) | 0%  (0) | 0%  (0) | 20%  (5) | 0%  (0) | 30%  (7) | 50%  (12) | 20%  (5) | 0%  (0) | 0%  (0) |
| **Eastern European** | Croatia | 0.116 | 1 | 20 | 100%  (1) | 100%  (1) | 70%  (14) | NA | NA | 70%  (14) | 0%  (0) | 0%  (0) | 0%  (0) | 100%  (20) | 0%  (0) | 0%  (0) | 0%  (0) | 100%  (20) | 0%  (0) | 0%  (0) | 0%  (0) | 0%  (0) |
|  | Hungary | 0.233 | 1 | 74 | 0%  (0) | 24%  (1) | 30%  (21) | NA | NA | 30%  (21) | 3%  (2) | 6%  (5) | 16%  (12) | 52%  (38) | 2%  (2) | 22%  (17) | 0%  (0) | 66%  (49) | 22%  (17) | 10%  (7) | 2%  (1) | 0%  (0) |
|  | Lithuania | 0.124 | 1 | 53 | 0%  (0) | 0%  (0) | 32%  (14) | 16%  (1) | NA | 30%  (15) | 4%  (2) | 0%  (0) | 12%  (6) | 48%  (25) | 0%  (0) | 42%  (22) | 0%  (0) | 76%  (40) | 18%  (10) | 6%  (3) | 0%  (0) | 0%  (0) |
|  | Poland | 0.115 | 8 | 273 | 46%  (6) | 36%  (14) | 30%  (81) | 34%  (1) | NA | 30%  (82) | 3%  (7) | 0%  (1) | 8%  (22) | 62%  (167) | 2%  (6) | 28%  (77) | 0%  (0) | 90%  (243) | 8%  (22) | 2%  (8) | 0%  (0) | 0%  (0) |
| **Latin America and Caribbean** | Argentina | 0.328 | 1 | 40 | 0%  (0) | 42%  (9) | 44%  (18) | NA | NA | 44%  (18) | 0%  (0) | 0%  (0) | 0%  (0) | 0%  (0) | 84%  (34) | 16%  (6) | 0%  (0) | 16%  (6) | 84%  (34) | 0%  (0) | 0%  (0) | 0%  (0) |
|  | Brazil | 0.408 | 4 | 220 | 76%  (6) | 78%  (22) | 56%  (108) | 44%  (10) | NA | 54%  (118) | 1%  (2) | 0%  (2) | 0%  (0) | 0%  (0) | 84%  (185) | 16%  (33) | 0%  (0) | 16%  (33) | 84%  (185) | 0%  (0) | 0%  (1) | 0%  (1) |
|  | Chile | 0.247 | 1 | 32 | 100%  (1) | 50%  (4) | 24%  (8) | NA | NA | 24%  (8) | 0%  (0) | 0%  (0) | 10%  (3) | 6%  (2) | 38%  (12) | 46%  (15) | 0%  (0) | 88%  (28) | 6%  (2) | 6%  (2) | 0%  (0) | 0%  (0) |
|  | Mexico | 0.322 | 2 | 70 | 50%  (1) | 42%  (14) | 32%  (22) | NA | NA | 32%  (22) | 0%  (0) | 0%  (0) | 0%  (0) | 2%  (1) | 72%  (51) | 26%  (18) | 0%  (0) | 28%  (19) | 72%  (51) | 0%  (0) | 0%  (0) | 0%  (0) |
| **Western European and Other** | Australia | 0.097 | 6 | 182 | 14%  (1) | 48%  (12) | 46%  (62) | 44%  (19) | NA | 46%  (81) | 2%  (4) | 2%  (2) | 12%  (22) | 0%  (1) | 0%  (1) | 86%  (156) | 0%  (0) | 90%  (164) | 8%  (13) | 2%  (5) | 0%  (0) | 0%  (0) |
|  | Belgium | 0.043 | 1 | 32 | 0%  (0) | 0%  (0) | 44%  (4) | 60%  (14) | NA | 56%  (18) | 0%  (0) | 0%  (0) | 4%  (1) | 0%  (0) | 12%  (4) | 84%  (26) | 3%  (1) | 84%  (26) | 12%  (4) | 4%  (1) | 0%  (0) | 3%  (1) |
|  | Canada | 0.080 | 8 | 270 | 40%  (4) | 36%  (11) | 40%  (100) | 38%  (8) | NA | 40%  (108) | 0%  (1) | 2%  (3) | 4%  (11) | 0%  (0) | 2%  (3) | 94%  (253) | 0%  (0) | 96%  (258) | 4%  (12) | 0%  (0) | 0%  (0) | 0%  (0) |
|  | Denmark | 0.038 | 3 | 107 | 0%  (0) | 38%  (8) | 28%  (29) | NA | NA | 28%  (29) | 1%  (1) | 0%  (0) | 20%  (22) | 0%  (1) | 0%  (1) | 78%  (83) | 0%  (0) | 88%  (94) | 12%  (12) | 0%  (1) | 0%  (0) | 0%  (0) |
|  | Finland | 0.047 | 2 | 113 | 0%  (0) | 24%  (3) | 22%  (9) | 36%  (26) | NA | 30%  (35) | 0%  (0) | 0%  (0) | 2%  (2) | 0%  (1) | 0%  (0) | 98%  (110) | 0%  (0) | 100%  (113) | 0%  (0) | 0%  (0) | 0%  (0) | 0%  (0) |
|  | France | 0.049 | 6 | 200 | 24%  (2) | 34%  (14) | 32%  (60) | 0%  (0) | NA | 30%  (60) | 3%  (6) | 8%  (16) | 12%  (24) | 2%  (3) | 2%  (3) | 76%  (154) | 0%  (0) | 88%  (175) | 4%  (6) | 6%  (13) | 4%  (6) | 0%  (0) |
|  | Germany | 0.084 | 28 | 1400 | 20%  (8) | 22%  (70) | 26%  (311) | 26%  (57) | NA | 26%  (368) | 1%  (20) | 0%  (13) | 14%  (194) | 6%  (91) | 2%  (29) | 76%  (1073) | 0%  (0) | 86%  (1209) | 12%  (156) | 2%  (35) | 0%  (0) | 0%  (0) |
|  | Ireland | 0.093 | 2 | 52 | 0%  (0) | 54%  (8) | 52%  (27) | NA | NA | 52%  (27) | 2%  (1) | 2%  (1) | 0%  (0) | 0%  (0) | 0%  (0) | 98%  (51) | 0%  (0) | 98%  (51) | 2%  (1) | 0%  (0) | 0%  (0) | 0%  (0) |
|  | Italy | 0.069 | 6 | 262 | 0%  (0) | 44%  (26) | 28%  (56) | 16%  (10) | NA | 26%  (66) | 0%  (1) | 0%  (1) | 4%  (10) | 2%  (6) | 2%  (3) | 92%  (242) | 0%  (0) | 94%  (247) | 4%  (11) | 2%  (4) | 0%  (0) | 0%  (0) |
|  | Netherlands | 0.043 | 76 | 3559 | 22%  (26) | 32%  (220) | 26%  (732) | 24%  (157) | NA | 26%  (889) | 2%  (63) | 2%  (56) | 18%  (643) | 2%  (74) | 2%  (105) | 76%  (2681) | 0%  (0) | 82%  (2919) | 14%  (495) | 4%  (141) | 0%  (4) | 0%  (0) |
|  | New Zealand | 0.123 | 2 | 37 | 0%  (0) | 34%  (2) | 28%  (10) | NA | NA | 28%  (10) | 0%  (0) | 0%  (0) | 32%  (12) | 8%  (3) | 2%  (1) | 56%  (21) | 0%  (0) | 62%  (23) | 22%  (8) | 16%  (6) | 0%  (0) | 0%  (0) |
|  | Spain | 0.070 | 3 | 91 | 34%  (1) | 64%  (9) | 46%  (41) | NA | NA | 46%  (41) | 0%  (0) | 0%  (0) | 0%  (0) | 0%  (0) | 4%  (4) | 96%  (87) | 0%  (0) | 96%  (88) | 4%  (3) | 0%  (0) | 0%  (0) | 0%  (0) |
|  | Switzerland | 0.025 | 18 | 952 | 16%  (4) | 26%  (29) | 26%  (230) | 36%  (25) | NA | 28%  (255) | 1%  (12) | 2%  (21) | 20%  (192) | 2%  (25) | 2%  (22) | 72%  (691) | 0%  (1) | 78%  (750) | 12%  (112) | 8%  (85) | 0%  (4) | 0%  (1) |
|  | United Kingdom | 0.118 | 229 | 10685 | 28%  (86) | 38%  (661) | 36%  (3154) | 34%  (574) | 38%  (9) | 36%  (3728) | 1%  (96) | 4%  (435) | 14%  (1448) | 2%  (204) | 2%  (317) | 78%  (8276) | 0%  (5) | 84%  (8917) | 12%  (1245) | 4%  (453) | 0%  (60) | 0%  (10) |
|  | United States | 0.204 | 158 | 7838 | 26%  (51) | 38%  (459) | 38%  (2701) | 28%  (191) | 68%  (42) | 38%  (2892) | 1%  (64) | 2%  (123) | 8%  (655) | 0%  (38) | 2%  (204) | 86%  (6814) | 0%  (4) | 90%  (7067) | 8%  (612) | 2%  (136) | 0%  (15) | 0%  (8) |

**IF:** impact factor, **EiC**: editors-in-chief, **EL**: editorial leadership, **EB**: editorial board, **AB**: advisory board, **EC:** early career/young researchers, **Unkn:** unknown; **AF:** Africa, **AP:** Asia and Pacific,  **EE:** Eastern Europe, **LAC:** Latin America and the Caribbean, **WEO:** Western Europe and Other, **HIC:** high-income countries, **UMIC:** upper-middle-income countries, **LMIC:** lower-middle-income countries, **LIC:** low income countries
