# Supplementary material for: Challenging the “old boys club” in academia: Gender and geographic representation in editorial boards of journals publishing in environmental sciences and public health
Source: PLOS Glob Public Health. 2022 Jun 21;2(6):e0000541. doi: 10.1371/journal.pgph.0000541 (PMC10021803; doi:10.1371/journal.pgph.0000541)
Supplement: Attachment — Submitted filename: PLOSGPH Response to reviewer comments.docx [file pgph.0000541.s010.docx]

Dear Editorial Team,

Thank you for sharing the below reviewer comments on our manuscript entitled “**Challenging the “Old Boys Club” in Academia: Gender and Geographic Representation in Editorial Boards of Journals Publishing in Environmental Sciences and Public Health.**”

We appreciate the opportunity to strengthen our manuscript based on the constructive feedback of the reviewers.

After incorporating these constructive comments from the reviewers, we believe this article will be a valuable contribution to *PLOS Global Public Health* and its publication could contribute to meaningful conversations around knowledge creation and dissemination in academia, and specifically in the pertinent field of planetary health and climate change.

Finally, we would like to thank the reviewers for their incredibly helpful comments and feedback that we believe have strengthened the manuscript. We have likewise acknowledged the contribution of the reviewers in our acknowledgement section “The authors thank the peer-reviewers for their constructive feedback that enabled the improvement of the manuscript.”

Please see our responses to the journal and reviewers’ comments below.

Sara Dada and Kim van Daalen

**Journal Requirements:**

1. Your co-authors: Laura Jung [laura.kunignde@gmail.com](mailto:-laura.kunignde@gmail.com); Jiaqi Li [jl2006@cam.ac.uk](mailto:-jl2006@cam.ac.uk); Ebele R. I. Mogo [ebele@erimconsulting.org](mailto:ebele@erimconsulting.org), have not confirmed authorship of the manuscript. We have resent them the authorship confirmation email; however please check that the above email address for them is correct and follow up personally to ensure they confirm. Please note that we cannot proceed your manuscript  until we have received confirmations from all co-authors.

**RESPONSE:**  We apologize for the inconvenience. Our co-authors should now have confirmed their co-authorship of the manuscript.

2. Please amend your detailed Financial Disclosure statement. This is published with the article, therefore should be completed in full sentences and contain the exact wording you wish to be published. i) Please include all sources of funding (financial or material support) for your study. List the grants (with grant number) or organizations (with url) that supported your study, including funding received from your institution.  ii). State the initials, alongside each funding source, of each author to receive each grant. iii). State what role the funders took in the study. If the funders had no role in your study, please state: “The funders had no role in study design, data collection and analysis, decision to publish, or preparation of the manuscript.” If you did not receive any funding for this study, please simply state: “The authors received no specific funding for this work.”

**RESPONSE:** We have amended this statement accordingly.

*Adapted text:* “The authors received no specific funding for this work. KRvD receives funding by the Gates Cambridge Scholarship (OPP1144) for her PhD research and received funding for publication from the Bill and Melinda Gates Foundation.” (page 23)

3. Please provide  separate figure files in .tif or .eps format only and remove any figures embedded in your manuscript file.  Please ensure that all files are under our size limit of 20MB.

**RESPONSE:** We have uploaded these figure .tif files and removed them from the manuscript file.

4. In the online submission form, you indicated that [Insert text from online submission form here]. All PLOS journals now require all data underlying the findings described in their manuscript to be freely available to other researchers, either 1. In a public repository, 2. Within the manuscript itself, or 3. Uploaded as supplementary information. This policy applies to all data except where public deposition would breach compliance with the protocol approved by your research ethics board. If your data cannot be made publicly available for ethical or legal reasons (e.g., public availability would compromise patient privacy), please explain your reasons by return email and your exemption request will be escalated to the editor for approval. Your exemption request will be handled independently and will not hold up the peer review process, but will need to be resolved should your manuscript be accepted for publication. One of the Editorial team will then be in touch if there are any issues.

**RESPONSE:**  Our data is openly available in a Github repository. We have changed our data availability statement accordingly.

*Adapted text:* “The data that support these findings of this study are openly available in:<https://github.com/KimvanDaalen/EBrepresentation>. These data were derived from webpages of included journals in this study.” (page 22)

5. Please update the completed 'Competing Interests' statement, including any COIs declared by your co-authors. If you have no competing interests to declare, please state "The authors have declared that no competing interests exist"

**RESPONSE:** We have amended this statement accordingly.

*Adapted text: “*The authors have declared that no competing interests exist.” (page 23)

6. We have noticed that you have uploaded supporting information but you have not included a list of legends.  Please add a full list of legends for all supporting information files (including figures, table and data files) after the references list.

**RESPONSE:** We have included a list of legends for all figures, tables, and supplement tables/figures after the reference list (page 28-30).

7. Please review your reference list to ensure that it is complete and correct. If you have cited papers that have been retracted, please include the rationale for doing so in the manuscript text, or remove these references and replace them with relevant current references. Any changes to the reference list should be mentioned in the rebuttal letter that accompanies your revised manuscript. If you need to cite a retracted article, indicate the article’s retracted status in the References list and also include a citation and full reference for the retraction notice.

**RESPONSE:** We have reviewed the reference list and confirm that it is complete and correct.

**Reviewer #1:** I congratulate the authors on a well-researched and well-written article. The topic is timely given ongoing interest in the power dynamics surrounding the construction of knowledge, especially in the field of global public health. I offer two key areas for thought related to the methods and discussion.

8. How did the author's address individuals who had multiple affiliations--perhaps an affiliation at universities both in LMICs and HICs (as can be common among GH practitioners)? (Lines 200-203).

**RESPONSE:** We have clarified that we used the first institutional affiliation associated with the individual as listed on the editorial board pages (or in their publications), under the assumption that this is their primary affiliation.

*Adapted text:* “Where more than one institution was listed, we used the initial or primary affiliation provided.” (page 8, lines 204-205)

9. Why were student-run editorial boards excluded (lines 181-182)? Quick reflection on such data could reveal prospects for changes for the future or offer a data point for similar studies to be conducted in the future.

**RESPONSE:** We decided to exclude the exclusively student-run journals (n=3) that were also all specific-university-affiliated because we did not use a database that explicitly included all university-student-run publications. Therefore, including these three journals in the analysis may have been a biased subset of all student-run publications. Additionally, these publications were affiliated with specific universities and so all members involved in the journal were from the same institutional affiliation, which would influence any geographic analysis of these journals as well. We have clarified in the manuscript that these journals were specifically run by university students. We did not exclude students as part of broader editorial boards (e.g., “Trainee Editorial Board Member”).

*Adapted text:* “Journals with no publicly available information about their editorial boards and exclusively university-affiliated student-run journals were excluded.” (page 8, line 182)

10. It is important to discuss the role that women play in global public health, especially on-the ground. Community Health Workers/Representatives are overwhelmingly female (see Love 1997, and more recent numbers from WB). This means that we depend on women (and many women of color) to carry out policies and programming, but they are not equally represented in editorial boards. Inclusion of such information in your discussion only strengthens your argument on the production of knowledge.

**RESPONSE:** This is a great point made by the reviewer and we agree that it is important to highlight this disparity in labor force versus positions of leadership/power. We have incorporated this into the discussion.

*Adapted text: “*Additionally, the majority of community health workers/representatives are women, including women of color.[41, 42] Yet, while women conduct a significant portion of global health work on the ground, they are not equally represented in global health leadership positions nor in the editorial boards of scientific journals.[43, 44]” (pages 17-18, lines 467-471).

11. More discussion of the limitation of not considering ethnicity would also strengthen the piece. Though I completely agree with the decision not to include it in the present analysis, some discussion of how editorial staff from LMICs is only a first step. Those from LMICs likely do not represent ethnic/linguistic/racial minorities. This is another part of the ongoing legacy of the coloniality of global public health.

**RESPONSE:** We agree with the reviewer that there are additional minority populations that should not be ignored in considering the current disparities in journal editorial boards. We have incorporated further discussion of this limitation and suggested future research, and ‘journal interventions,’ must consider these populations.

*Adapted text: “*While this analysis provides insight into the representation of gender and geography, this is only the first step. It is important for future work (both research and journal editorial board policies) to also consider ethnic, linguistic, socioeconomic, and racial minorities and to ensure that these perspectives are not further marginalized in the processes of knowledge creation and dissemination.” (page 21, lines 5448-552).

12. Lines 138-139: "field's ability to...improved health for all" This conclusion seems to be a jump from the evidence presented in this paragraph. Add 1-2 sentences of evidence or consider moving to the next paragraph where it seems to more accurately reflect the information presented.

**RESPONSE:** We agree with the reviewer and have removed the sentence from the manuscript accordingly.

13. Line 152: Consider adding male to the White, Western lens

**RESPONSE:** We understand the reviewer’s point here but would like to respectfully keep the line as is. We feel that the overall decolonizing movement described in this sentence is focused on the ‘White, Western’ lens and that we do discuss the dominance of men (and White, Western men) in other aspects of the introduction and discussion.

14. Line 163: the phrase "contributes to shaping tomorrow" is accurate but feels trite, consider revision

**RESPONSE:** We agree with the reviewer and have rephrased this in the manuscript.

*Adapted text:* “The work published today contributes to scientific progress and future policy-making.” (page 7, lines 166-167)

15. Line 180: remove "would"

**RESPONSE:** We have removed this word (page 8, line 181).

16. Lines 194-196: Should these journal titles be italicized in text?

**RESPONSE:** We have italicized the titles of these journals (page 8, lines 195-197).

17. Lines 208-210: Remove GII index explanation- no other metrics are explained in this section

**RESPONSE:** We have removed the more detailed explanation but kept the line *“Higher GII values correspond to increased disparities between women and men in these areas”* in order to provide useful context to interpret the results for readers who are not familiar with GII (page 9, lines 210-211).

18. Line 255: Capitalize I in Confidence Interval abbreviation

**RESPONSE:** Thank you to the reviewer for catching this typo. We have adjusted it accordingly (page 10, line 261).

19. Line 494: Insert % (and number of individuals) for whom the gender-to-name algorithm was used

**RESPONSE:** Thank you to the reviewer for pointing out this oversight. We have included the correct percentage and number of individuals.

*Adapted text:* “a gender-to-name algorithm was used for 7.6% (n=2,170) of individuals” (page 21, line 557).

20. Throughout results & discussion: I am unsure of the style guide's preference for using % to mean percent when no number is offered. For example, % overall WGM (line 360) versus percent overall WGM.

**RESPONSE:** We have reviewed these sections and made this consistently *“percentage of”* throughout the text of the manuscript.

**Reviewer #2:** This is a compelling and clearly written analysis of editorial boards for journals in the environmental sciences and public health space. The multiple authors have detailed their methods and impressive efforts in compiling a unique database of editorial board members for the relevant journals. Writing is excellent overall. Tables and figures are compelling and emphasize important results.

21. Line 109 - 'published in as scientific' - should be "published as scientific"

**RESPONSE:** Thank you to the reviewer for catching this typo, which we have now fixed (page 5, line 109).

22. Line 494 - "for X% of individuals" - should be replaced with the final percentage (7.6%?)

**RESPONSE:** Thank you to the reviewer for pointing out this oversight. We have included the correct percentage and number of individuals.

*Adapted text:* “a gender-to-name algorithm was used for 7.6% (n=2,170) of individuals” (page 21, line 557).
